# Molecular regulatory mechanisms of dietary supplementation with *Allium mongolicum* Regel powder to improve muscle development and meat quality in Angus calves

**DOI:** 10.5713/ab.24.0809

**Published:** 2025-02-27

**Authors:** Wangjing Liu, Chenxu Sun, Huixia Gao, Jianjian He, Aihuan Yu, Yaodi Xie, Haibo Yao, Jiang Hu, Zhaomin Lei

**Affiliations:** 1 College of Animal Science and Technology, Gansu Agricultural University, Lanzhou, Gansu, China

**Keywords:** *Allium mongolicum* Regel, Meat Quality, Metabolic Pathway, Muscle Development, Signaling Pathway

## Abstract

**Objective:**

Supplementing animal feed with *Allium mongolicum* Regel powder (AMRP) additives can promote muscle production and improve meat quality. Here, we explored the effects of dietary AMRP supplementation on the performance, meat quality, and muscle transcriptome profile of Angus calves.

**Methods:**

Twelve healthy female black Angus calves (average body weight = 280.4±15.74 kg, average age = 14±0.6 months) of the same genetic background were randomly assigned to two feed groups: control (CON; basal diet without any supplementation) and AMRP (basal diet supplemented with 20 g of AMRP per calf per day).

**Results:**

In general, compared with the control group, dietary AMRP supplementation significantly increased the longissimus thoracis muscle area (p = 0.027) and pH_24h_ (p = 0.027) but significantly reduced Warner-Bratzler shear force (p = 0.009) and cooking loss (p<0.001). Moreover, 1,284 differentially expressed genes (DEGs) were identified in AMRP-supplemented Angus calves. Pathway analysis revealed that the DEGs were involved in multiple pathways related to muscle development and fat deposition, such as the focal adhesion and MAPK pathways.

**Conclusion:**

Dietary supplementation of AMRP improved muscle growth and development in Angus beef cattle. It also significantly modulated meat quality, possibly altering signaling pathways by influencing key gene expression. Our results provide novel insights into the development of the meat industry and indicate the mechanism through which AMRP regulates muscle development and improves meat quality at the molecular level.

## INTRODUCTION

In 2023, China was the second largest consumer of beef (10.27 million t) globally after the United States (12.18 million t) (United States Department of Agriculture). These data indicated that in China, traditional fiber-based diets are being replaced by meat-based Western diets not only because of rapid economic growth but also due to a major shift in nutritional perceptions among consumers [1]. According to a simulation of beef consumption based on household income in China, beef consumption is projected to increase by 12.0% to 38.8% over the next 10 years and by 18.6% to 70.5% over the next 15 years [2]. However, in China, beef production and its large consumer population demonstrate a severe imbalance. Therefore, breeders must improve muscle development in beef cattle to directly improve carcass muscle yield and thus increase productivity and profitability in the beef supply chain.

Animal growth is largely determined by the muscle and adipose tissue deposition rates in the body. Muscle growth involves several biological processes, such as coordinated expression of many transcription factors (myogenic regulators), genes, and metabolic pathways. In animals, the development period from the embryonic and fetal stages until maturity can be roughly divided into two processes. During the mid-to-late gestational stages, most changes in muscle weight occur due to an increase in secondary fiber growth. In contrast, postnatal muscle growth is mainly confined to myofibril size increase (or hypertrophy). Both these processes are regulated by genetic factors, growth factors (e.g., insulin-like growth factor), hormones, and feed nutrition, and they regulate animal growth positively or negatively. The protein synthesis and degradation process—often referred to as protein turnover—affects the muscle growth rate and thus alters the meat production characteristics of beef cattle [3]. Both lipogenesis (i.e., the process of lipid synthesis) and adipogenesis (i.e., the process of adipocyte development and lipid accumulation) function simultaneously to contribute to intramuscular fat (IMF) deposition in beef cattle [4]. Increases in cell number and size are associated with adipose tissue deposition. The results of a transcriptome analysis demonstrated a strong correlation between IMF deposition and meat quality, particularly tenderness [5]. Meat quality and carcass traits are influenced by a complex gene interaction network in muscles.

In 2020, China banned the addition of growth-promoting antibiotics to animal diets. This led to an increase in research on natural plant additives to promote animal growth and improve meat quality [6]. *Allium mongolicum* Regel, a perennial plant of the Amaryllidaceae family, is mainly found in semiarid regions of China, Mongolia, and Kazakhstan. The cold, semiarid growing environment in these areas imparts the plant with unique bioactive effects. We previously confirmed that *A. mongolicum* Regel improves growth performance, nutrient digestibility, meat quality, and meat flavor [7] in lambs; it even has antioxidant properties, which prolong the shelf-life of lamb meat [8]. These effects may be related to the main active ingredients in *A. mongolicum* Regel: flavonoids and polyphenolic compounds, all of which have anti-inflammatory and antioxidant properties, maintain gastrointestinal barrier integrity, and improve feed nutrient digestion and absorption in animals. However, to the best of our knowledge, the effects of dietary *A. mongolicum* Regel supplementation on calf growth performance, muscle development, and meat quality have not been reported thus far. In the current study, we hypothesized that as a polyphenolic-rich dietary supplement, *A. mongolicum* Regel powder (AMRP) improves muscle development and meat quality in Angus cattle. Therefore, in this study, we investigated the effects of dietary supplementation with AMRP on molecular mechanisms and metabolic pathways related to growth performance, carcass traits, and meat quality, particularly in terms of muscle growth and fat deposition, in beef cattle. Transcriptome analysis was used to screen differentially expressed genes (DEGs) associated with muscle development and fat deposition in Angus cattle and elucidate the underlying mechanisms based on key gene and pathway enrichment and protein interactions.

## MATERIALS AND METHODS

### *Allium mongolicum* Regel powder preparation

Fresh *A. mongolicum* Regel was collected over June to October 2022 from an *A. mongolicum* Regel plantation in Minqin County, Gansu, China, and transported to the laboratory in iceboxes at 4°C. Next, *A. mongolicum* Regel leaves were accurately separated, washed, and dried at <65°C in an electrothermal blast drying oven (DHG-9053A; Wuxi Marit Technology, Jiangsu, China). All leaves were weighed every 30 min to record weight loss. When the moisture content of all leaves decreased to <10%, the dried leaves were pulverized in PS-5 (Jiangsu Kewei Machinery, Jiangsu, China), passed through a 1-mm sieve, and stored at 4°C in the dark until use. As indicated in our previous work [9], the total contents of phenolics and flavonoids in AMRP were 21-milligram equivalents of gallic acid per gram dry weight and 42-milligram equivalents of rutin per gram dry weight, respectively.

### Animals and diets

This study was conducted in accordance with the recommendations of the National Institute of Animal Health of China’s Laboratory Animal-Requirements of Environment and Housing Facilities (GB 14925-2001) and approved by the Animal Ethics Committee of Gansu Agriculture University, Lanzhou, China (approval number: 2006-398). The study was conducted between April 3 and August 16, 2023, at the Longshengda Livestock Breeding, Tianjin, China (39°05′N, 116°88′E). Twelve healthy female black Angus calves (average body weight = 280.4±15.74 kg, average age = 14±0.6 months) of the same genetic background (Timaru, New Zealand) were randomly assigned to two feed groups: control (CON), basal diet without any supplementation, and AMRP, basal diet supplemented with 20 g of AMRP per calf per day. We selected the AMRP supplementation amount on the basis of the results of our previous experiments related to fattening Angus cattle: 20 g of AMRP per calf per day considerably improved meat quality [10]. AMRP was purchased from Ao Xiang Specialized Cooperative (Wuwei, China). We previously reported that the main active ingredients in this AMRP are flavonoids and polyphenols such as chrysoeriol, quercetin, and omoeriodictyol [8].

The calves were penned individually, and each pen (area = 2×5 m^2^) was equipped with a feed trough, as well as a water trough, allowing for free access to fresh water. During the entire feeding trial cycle, the basal diet composed, on a dry matter basis, of 38.31% rice straw, 19.54% guinea grass, 16.66% corn silage, 6.63% soybean meal, 6.29% corn, 5.56% corn germ meal, 5.51% wheat bran, and 1.5% vitamin premix. The dietary nutrient and chemical composition was as follows: net energy for maintenance, 5.63 kJ/kg; net energy for gain, 2.63 kJ/kg; ash, 12.60%; crude protein, 11.84%; and ether extract, 2.20%. The calves were adapted to the diet for 15 days before a 120-day growth performance trial. The weight of each calf was recorded on experimental days 1 (i.e., initial weight) and 120 (i.e., final weight). The average daily gain was determined as the difference between the initial and final body weights. During the entire experimental period, we recorded the temperature and humidity changes in the feeding shed in the morning (08:00 h), noon (12:00 h), and evening (20:00 h).

### Slaughter and carcass characteristics

One calf each in the AMRP and CON groups died due to diarrhea caused by extreme weather during the experimental period. One week after the end of the experimental feeding period, calves were weighed before loading; this weight was considered the preslaughter weight. All 18-month-old animals were fasted for 12 h, deprived of water for 2 h, and slaughtered immediately by professionals in a commercial slaughterhouse (117°14′N, 39°22′E; Tianjin, China), located 55 km from the study site. Eviscerated carcasses were then weighed to determine the hot carcass weight (HCW). Dressing percentage was calculated as follows: (HCW/preslaughter weight)×100. The left *longissimus thoracis* muscle (LT) area was calculated 24 h after slaughter in the 12th rib by using a planimeter (KP-90N; Koizumi Furnitech, Japan). HCW was used as a covariate in the statistical analysis of the LT area data to determine whether the diet affected the LT area independent of increases in HCW. The left LT was excised from each carcass for meat physicochemical analyses.

### *Longissimus thoracis* characteristic measurement

At 45 min and 24 h after slaughter, the LT pH was measured using a portable pH meter (S400-K; Mettler Toledo, Zurich, Switzerland). The pH meter was calibrated using standard pH 7.0 and 4.0 buffers at 25°C. Instrumental meat color (lightness [L*], redness [a*], and yellowness [b*]) were measured using an Opto-lab Matthaus (OPTO-LAB, Matthaus, Germany) with D65 as the illuminant, observer angle of 10°, and diameter aperture of 26 mm. Drip loss was determined using Honikel’s method [11] with minor modifications. The meat samples (size = 20×10×10 mm^3^, weight = 20 to 30 g) were hooked onto plastic bottles and placed in a refrigerator at 4°C for 24 h. Drip loss was calculated as follows: (weight loss/initial weight)×100. To assess cooking loss, all samples were weighed on a Secura electronic balance (M20S; Sartorius, Berlin, Germany), wrapped in plastic, and placed in a thermostatic water bath at approximately 80°C. Temperatures were monitored using a temperature probe (Hanna Instruments, Bellville, South Africa) along with a thermocouple thermometer (WRNR-12; Shanghai Wenhuang Automation Instrumentation, Shanghai, China). The process was terminated when the internal temperature of the meat sample reached 70°C. After the meat samples were cooled to 20°C, they were weighed, and the cooking loss was calculated as follows: [(weight after cooking–weight before cooking)/precooking weight before cooking]×100. The cooked samples were chilled at 4°C for 24 h, and a coring drill was used to take six random samples parallel to the muscle fibers on whole meat samples. Next, a Warner–Bratzler shear machine (RH-N50; Runhu, Guangzhou, China) was used with the blade cutting perpendicular to the direction of the muscle fibers at 200 mm/min to determine the Warner-Bratzler shear force (WBSF). Shear force blocks were randomly allocated to cook batches on an individual muscle basis and balanced for slaughter day and treatment.

### RNA extraction, library construction, transcriptome analysis, and differentially expressed gene identification

RNA was extracted from 10 LT samples by using TransZol (ER501-01; Takara, Shiga, Japan). In total, 1 μg RNA per sample was used as input material for RNA sample preparation. RNA was tested for integrity, purity, and concentration through agarose gel electrophoresis (AGE; AGE-102-1.0; Nordic-Mubio, Shanghai, China), spectrophotometry (Nanodrop 2000; Thermo Fisher Scientific, Waltham, MA, USA), and bioanalyzer-based analysis (Agilent 2100 Bioanalyzer; Agilent Technologies, Santa Clara, CA, USA), respectively. After enrichment of eukaryotic mRNAs with polyA tails by using magnetic beads with Oligo (dT), the mRNAs were fragmented through ultrasound. The fragmented mRNA was then used as a template and random oligonucleotides as primers to synthesize the first strand of cDNA in the M-MuLV reverse transcriptase system, followed by the degradation of the RNA strand with RNaseH and synthesis of the second strand of cDNA with dNTPs in the DNA polymerase I system. The purified double-stranded cDNA was end-repaired, A-tailed, ligated to the sequencing junction, and then screened for approximately 200-bp cDNAs by using AMPure XP beads. Polymerase chain reaction (PCR) amplification was performed, and the PCR products were purified again using AMPure XP beads. Finally, Gene Denovo Biotechnology (Guangzhou, China) constructed libraries with the paired-end sequencing strategy PE150. Quality control of the constructed libraries was accomplished using a high-sensitivity DNA assay Kit (5067-4626; Agilent Technologies). Raw reads were quality-controlled offline by using fastp (version 0.19.7), filtering out low-quality data until a sufficient number of clean reads were obtained; this was immediately followed by ribosome matching, removal of reads from the upper ribosome of the match when mismatches were not allowed, and retention of the unmapped reads for the subsequent transcriptome analyses. The short-read alignment tool Bowtie2 (version 2.2.8) was used for mapping reads to a ribosomal RNA (rRNA) database. The rRNA-mapped reads were then removed. The remaining clean reads were further used in assembly and gene abundance calculations. Finally, HISAT2 (version 2.1.7) [12] was used to paired-end clean map reads to the reference genome based on ARS-UCD 1.2 of *Bos taurus* (https://www.ncbi.nlm.nih.gov/search/all/?term=Bos+taurus&source=taxonomy) and other parameters set as a default. On the basis of the HISAT2 comparison results, we reconstructed the transcripts by using Stringtie [13]. To quantify the expression abundance and variation of each transcription region, we calculated its TPM (i.e., Transcripts Per Kilobase of exon model per Million mapped reads) by using the software program RSEM [14]. RNA differential expression analysis was performed by DESeq2 (version 1.20.0) between two groups. The criteria for defining DEGs were |log2(fold change)| of ≥1.00 and false discovery rate (FDR) of <0.05 [15]; the DEGs were visualized using volcano plots and through hierarchical clustering analysis in the R packages ggplot2 (version 3.3.6) and pheatmap (version 1.0.12).

### Functional annotation and enrichment analysis

Gene Ontology (GO) analysis is used to categorize and annotate GO functions of DEGs, classified in terms of molecular functions, cellular components, and biological processes; it can also aid in analyzing significant enrichment of the DEGs’ GO functions. In particular, DEGs were mapped to each term in the GO database (http://www.geneontology.org/), and the number of genes in each term was calculated to obtain a list of genes with certain GO functions and the gene number statistics; next, the hypergeometric test was applied to calculate p-values. The calculated p-values were corrected using Bonferroni correction, and the GO terms that fulfilled the threshold of corrected p (≤0.05) were defined as those significantly enriched in the DEGs, which could be analyzed to determine the main biological functions of the DEGs.

To identify crucial pathways involving the DEGs, we used the Kyoto Encyclopedia of Genes and Genomes (KEGG) pathway-based significant enrichment can be used to identify the most important metabolic and signaling pathways involving DEGs. We used the Fisher exact test, with a significance threshold of p<0.05, to identify enriched pathways.

DAVID was used for functional annotation and enrichment analyses of the DEGs. Known gene annotation information for this project was downloaded from Ensembl Biomart. New gene annotations were performed using BLAST to compare data from the nr database (20220220) for description annotation, the KEGG database (Release 101; http://www.kegg.jp) for KEGG annotation, and eggNOG DB (5.0.2) (http://eggnog5.embl.de/download/emapperdb-5.0.2/) for GO annotation. GO enrichment analysis was performed using GO.db (3.14.0), and KEGG enrichment analysis was performed using self-written scripts.

### Protein-protein interaction network analysis and hub gene determination

The STRING database (http://string-db.org) was used to create a DEG-based protein-protein interaction (PPI) network. For the species included in the database, we extracted a set of DEGs from the database, and by using Cytoscape (version 3.8.2), we constructed a PPI network graph, where nodes represented the genes and edges represented the PPI network. To determine the major hub genes, the combined score method was used to rank the nodes’ importance in the PPI network.

### Quantitative real-time reverse-transcription polymerase chain reaction confirmation of differentially expressed genes

Ten genes related to muscle metabolism, development, and fat deposition were randomly selected for quantitative real-time reverse-transcription PCR (qRT-PCR) analysis to verify the accuracy of the RSEM data. Total RNA was extracted from 0.05 g of frozen LT samples by using TRIzol reagent (Takara), according to the manufacturer’s instructions. By using 2% AGE and a Thermo Scientific Microplate Reader (Multiskan Spectrum, Shanghai, China) set to 260/280 nm (optical density at 260 nm/optical density at 280 nm = 1.8 to 2.1), we evaluated RNA concentration, purity, and integrity. First-strand cDNA was synthesized in a volume of 30 μL using 1 μg of total RNA and PrimeScript RT reagent (RR047A; Takara). A final volume of 20 μL was used for qRT-PCR, which included 10 μL of 1×SYBR Premix Ex TaqTM, 3 μL of cDNA, 0.4 μL of each of the forward and reverse primers (both 0.2 μM), and 6 μL of RNase-free water. All reactions were performed on a Light Cyber Roche 480 PCR system (Roche, Shenzhen, China) with the following conditions: an initial denaturing step of 95°C for 30 s, followed by 40 cycles of 95°C for 30 s (denaturation), different annealing temperatures (Table 1), and 72°C for 20 s (extension), and finally by 51 cycles of 70°C for 0.06 s. PCR amplification specificity was confirmed through 2% AGE and melting curve analysis. We performed qRT-PCR with three technical replicates and five biological replicates of cDNA for each analyzed gene. The glyceraldehyde-3-phosphate dehydrogenase and β-actin genes were used as reference genes. Table 1 lists the GenBank accession numbers and primer sequences of genes amplified through qRT-PCR. Finally, the 2^−ΔΔCt^ method was used to analyze the candidate characteristic LT genes in the qRT-PCR data.

### Statistical analyses

All data (i.e., carcass and LT characteristics and gene mRNA expression delta CT data) were analyzed using analysis of variance with GLM procedures in SPSS (version 22.0; SPSS, Chicago, IL, USA); they are presented as means±standard errors of the means. The model included fixed effects of dietary treatment, with the pen as a random effect. The shear force test date and cook batch were included in the random model to evaluate WBSF and cooking loss. The data presented as percentage ratios of dressing percentage, drip loss, cooking loss, and IMF were logarithmically transformed and statistically analyzed. p<0.05 in Duncan’s significant difference test was considered to indicate a significant difference, and the tendency to be significant was determined at 0.05≤p≤0.10. We used RnaSeqSampleSize to estimate the reasonableness of our sample size based on FDR error control in multiple testing and using the average read counts and discrete distributions of the real data. Covariates deemed nonsignificant were excluded from models using stepwise backward elimination before extraction of predicted means, standard errors, and least significant difference at 5% critical value.

## RESULTS

### Growth performance, carcass characteristics, and meat quality

The LT area was significantly larger in the AMRP group than in the CON group (p = 0.027; Table 2). The AMRP group also tended to have significantly higher HCW, dressing percentage, and IMF in the LT (p = 0.072, 0.078, and 0.052, respectively; Table 2). However, no other growth performance factor demonstrated a significant difference (p>0.05). LT pH_24h_ was significantly higher in the AMRP group than in the CON group (p = 0.027). In contrast, WBSF (p = 0.009) and cooking loss (p = 0.001) were significantly lower in the AMRP group than in the CON group. However, for pH45min, drip loss, and meat color in LT, we found no significant variations (all p>0.05; Table 2).

### RNA mapping and sequencing

Table 3 compares sample and reference genomic information mapping statistics. The CON and AMRP group LT samples were used to create 10 cDNA libraries, which were then sequenced to produce 10 sets of reads (CON 1 to 5 and AMRP 1 to 5, respectively). In total, 46,643,316, 42,298,252, 49,464,554, 39,138,000, 39,707,312, 47,527,944, 45,527,944, 43,430,132, 42,593,764, and 43,266,898 unmapped reads were obtained from CON 1, CON 2, CON 3, CON 4, CON 5, AMRP 1, AMRP 2, AMRP 3, AMRP 4, and AMRP 5, respectively (Table 3). The unique mapped rates were 91.99% to 92.68%.

### Differentially expressed gene analysis

**Figure 1:**
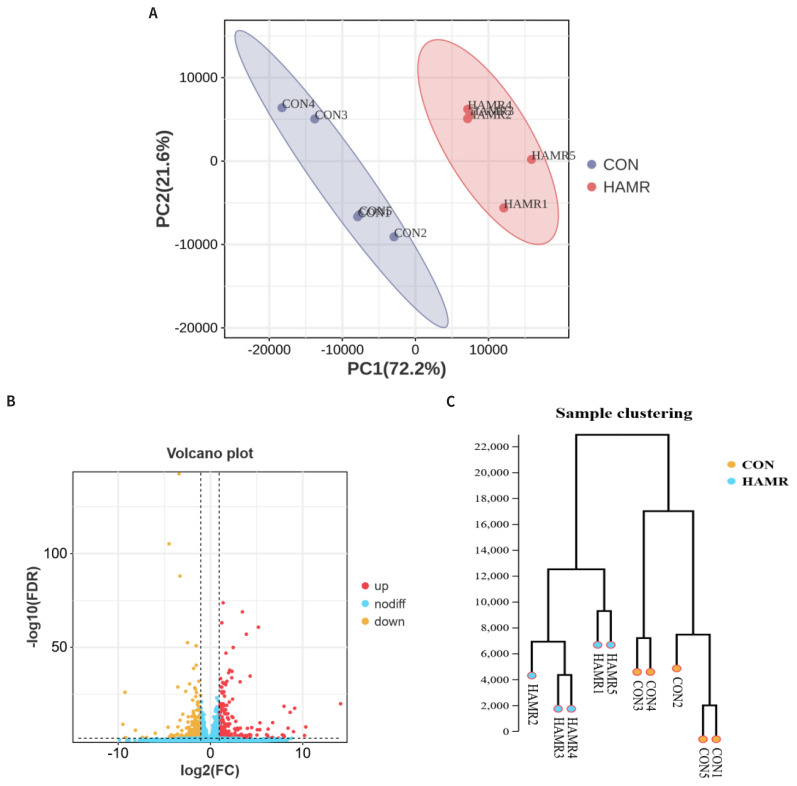
Principal component analysis plots (A), volcano plots (B), and sample clustering (C) of DEGs in LT of the CON and AMRP groups. The horizontal and vertical axes represent the first (PC1; 72.2%) and second (PC2; 21.6%) principal components, respectively. The blue area of the CON group is completely separated from the red area of the AMRP group (Figure 1A). Red dots (up) represent 1,001 significantly upregulated genes (|log2(fold change)| ≥1; FDR <0.05), blue dots (down) represent 283 significantly downregulated genes (|log2(fold change)| ≥1; FDR <0.05) in the AMRP group compared with the CON group, and black dots (nosig) represent nonsignificant DEGs (Figure 1B). Individual samples (five per experimental group, reported with a number from 1 to 5) and DEGs are indicated by columns and rows, respectively. The dendrogram was designed to intuitively represent the global expression patterns of the DEGs in the AMRP and CON groups (Figure 1C). FDR, false discovery rate; FC, fold change; DEGs, differentially expressed genes; AMRP, *Allium mongolicum* Regel powder; PC, principal component.

As displayed in Figure 1A, the blue area denoting the CON group is completely separated from the red area denoting the AMRP group; this result suggests that the two groups exhibited their respective clustered distributions. Figure 1B presents volcano plots of DEGs in the LT of the CON and AMRP groups. In total, 1,284 genes were considered DEGs corresponding to the effects of AMRP treatment, with |log2(fold change)| ≥ 1 and FDR <0.05; of these DEGs, 1,002 were upregulated [e.g., genes encoding AKT serine/threonine kinase 3 (*AKT3*), fibroblast growth factor (FGF) 6 (*FGF6*), epidermal growth factor (EGF) receptor (*EGFR*), Kirsten rat sarcoma viral oncogene (*KRAS*), and ceramide synthase (CERS) 4 (*CERS4*)] and 282 were downregulated [e.g., genes encoding myogenic differentiation 1 (*MYOD1*) and heat shock protein (HSP) family A (*HSP70*) member 6 (*HSPA6*)] in the LT of the AMRP group compared with the CON group (Figure 1B; Supplement 1). The examination of DEG clustering patterns between the AMRP and CON groups revealed that samples in the same group exhibited somewhat similar expression patterns (Figure 1C).

### Gene Ontology annotation and Kyoto Encyclopedia of Genes and Genomes pathway analyses

**Figure 2:**
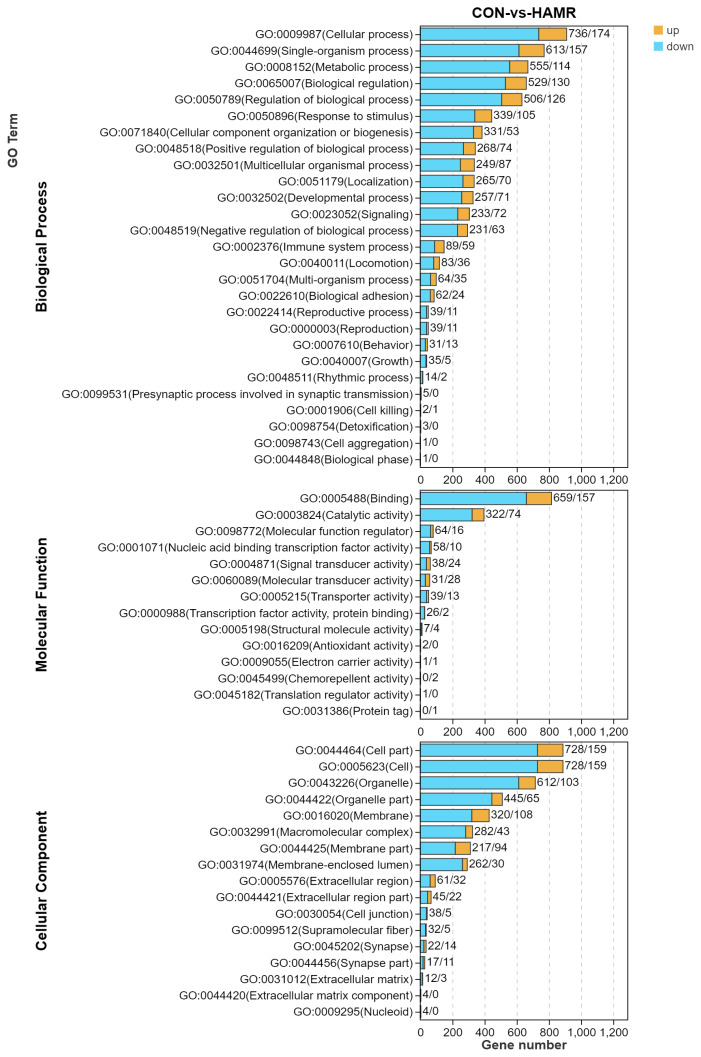
GO annotation of upregulated, downregulated, and all DEGs in the AMRP and CON groups. BP, MF, and CC denote the three GO categories biological processes, molecular functions, and cellular component, respectively. The horizontal axis represents the number of DEGs annotated to a GO term, whereas the vertical axis represents the GO terms. Yellow and blue indicate gene upregulation and downregulation in a clade, respectively. GO, Gene Ontology; DEGs, differentially expressed genes; AMRP, *Allium mongolicum* Regel powder; BP, biological process; MF, molecular function; CC, cellular component.

**Figure 3:**
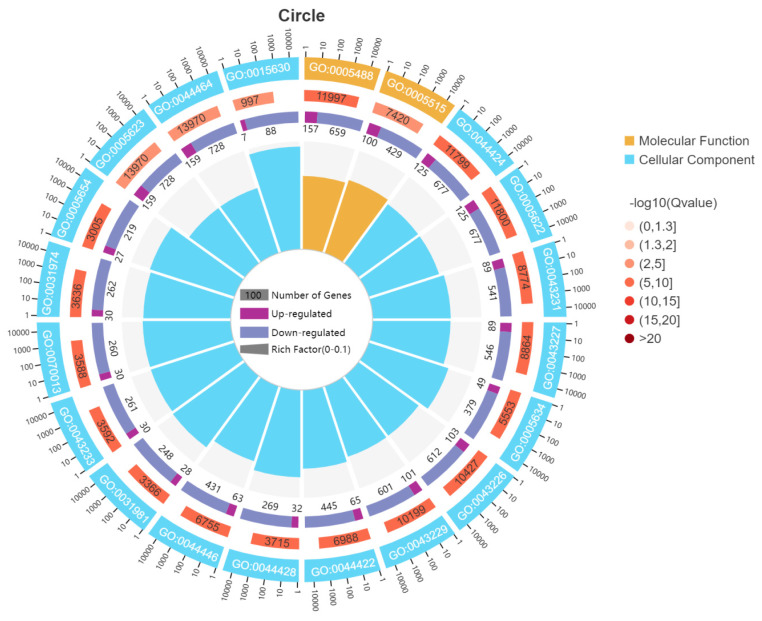
Top 20 enriched (*Q*≤0.05) terms in GO analysis of DEGs between the AMRP and CON groups. First circle: Top 20 enriched GO terms. The circle outside denotes a sitting scale for the numbers of DEGs. Different colors represent different GO terms. Second circle: The number of this GO term in the DEG background and Q value. The higher the number of DEGs, the longer in the bar, and the smaller is the Q value Adj. p≤0.05, the redder the color. Third circle: Upregulated and downregulated DEG ratio bar. Dark purple denotes the upregulated DEG ratio, and light purple represents the downregulated DEG ratio. Specific values are shown at the bottom. Fourth circle: RichFactor value of each GO term (DEG numbers in the GO term divided by the number of all genes in the GO term, with larger values indicating more significant enrichment). GO, Gene Ontology; DEGs, differentially expressed genes; AMRP, *Allium mongolicum* Regel powder.

Next, we mapped the 1,284 DEGs to all DEG modules annotated by the main GO categories (biological processes, molecular functions, and cellular component) and differentiated them between upregulated and downregulated modules (Figure 2). Function annotation results suggested that for all DEGs, the categories of biological process, molecular function, and cellular component comprised 27 GO terms (cellular, single-organism, and metabolic processes), 14 GO terms (binging, catalytic activity, and molecular function regulator), and 17 GO terms (cell parts, cells, and organelle). The top 20 enriched terms in GO enrichment analysis (Q value≤0.05) demonstrated that a few DEGs were enriched in the molecular function category, whereas most DEGs were enriched in the cellular component category (Figure 3; Supplement 2). In the cellular component category, intracellular part (GO: 0044424), intracellular membrane-bound organelle (GO: 0043231 and 0043227), and microtubule cytoskeleton (GO: 0015630) were the most primary subcategories. In the molecular function category, binding (GO: 0005488) and protein binding (GO: 0005515) were the most important subcategories (Supplement 3). Nevertheless, the biological process category did not contain any enriched GO terms.

**Figure 4:**
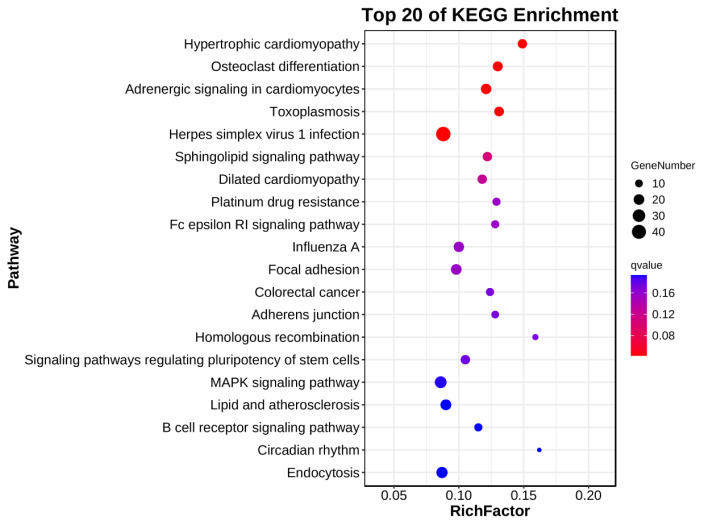
Enriched (p≤0.05) terms in the KEGG pathway analysis of DEGs between the AMRP and CON groups. RichFactor (represented by the horizontal axis) is the ratio of the number of DEGs enriched in a GO term to the number of DEGs enriched in that GO term in the background genes. The bubble size denotes the number of DEGs enriched in a GO term, whereas the bubble color represents the significance of enrichment in the GO term. The larger the *Q* value, the more significant is the difference. KEGG, Kyoto Encyclopedia of Genes and Genomes; DEGs, differentially expressed genes; AMRP, *Allium mongolicum* Regel powder; GO, Gene Ontology.

Figure 4 and Supplement 4 present the enriched (p≤0.05) terms in KEGG pathway analysis of the DEGs between the AMRP and CON groups. The results demonstrated that the top 20 significantly (p≤0.05) enriched pathways for all the DEGs belonged to the environmental information processing, cellular processes, and organismal systems categories; these pathways included sphingolipid pathway (ko04071; p = 0.002), focal adhesion (ko04510; p = 0.005), adherens junction (ko04520; p = 0.008), signaling pathways regulating stem cell pluripotency (ko04550; p = 0.008), MAPK pathway (ko04010; p = 0.010), circadian rhythm (ko04710; p = 0.012), phospholipase D (PLD) pathway (ko04072; p = 0.022), cholinergic synapse (ko04725; p = 0.038), and phosphatidylinositol signaling system (ko04070; p = 0.040). All these pathways are closely related to muscle growth, development, and lipid metabolism. Table 4 lists downregulated and upregulated DEGs enriched in these pathways.

### PPI network and hub genes

**Figure 5:**
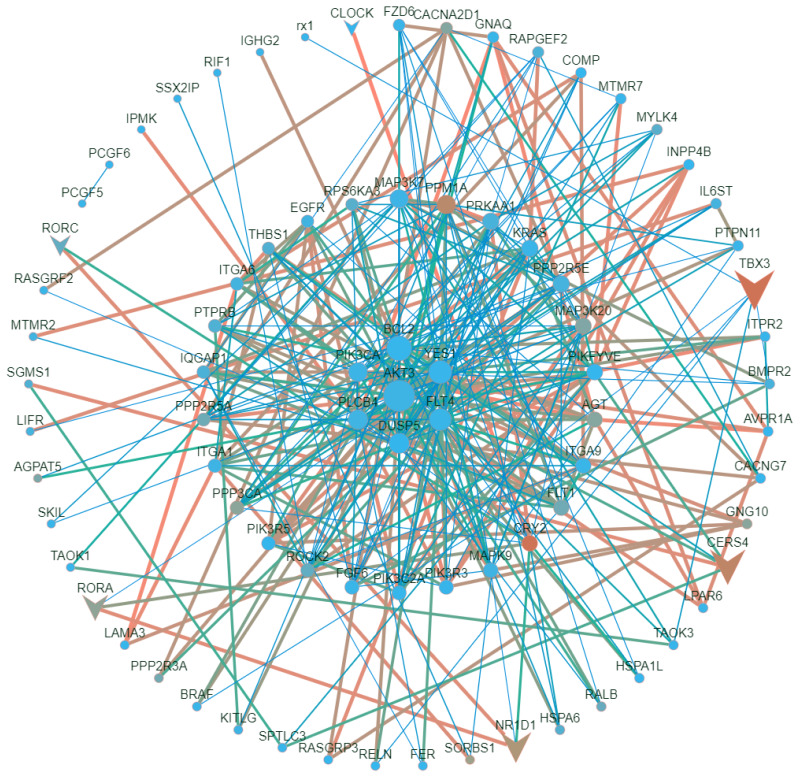
PPI network analysis and hub gene identification. Nodes (circles) are protein or gene names; the larger the circle, the better is the connectivity. The line segments indicate the gene–gene interactions or PPIs; the thicker the line segment, the stronger is the gene–gene interaction or PPI. The triangles are transcriptional regulators. The color of the node is indicated by the gene abundance (i.e., average of gene expression across all samples); the darker the color, the higher is the gene abundance. AKT3, AKT serine/threonine kinase 3; FGF6, fibroblast growth factor 6; EGFR, epidermal growth factor receptor; KRAS, Kirsten rat sarcoma viral oncogene; CERS4, ceramide synthase 4; MYOD1, myogenic differentiation 1; HSPA6, heat shock protein family A member 6; PLD, phospholipase D; BCL2, BCL2 apoptosis regulator; PIK3CA, phosphatidylinositol-4,5-bisphosphate 3-kinase catalytic subunit alpha; PLCB4, phospholipase C beta 4; YES1, YES proto-oncogene 1, Src family tyrosine kinase; MSCs, muscle satellite cells; IMPAs, intramuscular preadipocytes; ECM, extracellular matrix; ITGA1, integrin subunit alpha 1; ITGA9, integrin subunit alpha 9; ITGA6, integrin subunit alpha 6; MAP3K20, mitogen-activated protein kinase kinase kinase 20; MAP3K7, mitogen-activated protein kinase kinase kinase 7; LAMA3, laminin subunit alpha 3; COL6A5, collagen alpha-5(VI) chain isoform X1; CACNG7, calcium voltage-gated channel auxiliary subunit gamma 7; CACNG4, calcium voltage-dependent calcium channel gamma-4 subunit; DUSP5, dual specificity protein phosphatase 5; CACNG1, calcium voltage-dependent calcium channel gamma 1 subunit; DUSP13, dual specificity protein phosphatase 13; NR1D1, nuclear receptor subfamily 1 group D member 1; CLOCK, clock circadian regulator; CRY2, cryptochrome circadian regulator 2; RORA, RAR-related orphan receptor A; PIP2, phosphatidylinositol bisphosphate; PIP3, phosphatidylinositol triphosphate; DAG, diacylglycerol; PPI, protein-protein interaction.

Based on the results of our KEGG enrichment analysis, we selected pathways (p≤0.05) associated with muscle growth, development, and lipid metabolism to identify the hub genes. The nine KEGG pathways were ranked in order of significant enrichment as follows: sphingolipid pathway (ko04071), focal adhesion (ko04510), adherens junction (ko04520), signaling pathways regulating stem cell pluripotency (ko04550), MAPK pathway (ko04010), circadian rhythm (ko04710), PLD pathway (ko04072), cholinergic synapse (ko04725), and phosphatidylinositol system (ko04070). To construct a PPI network, we uploaded the DEGs involved in the KEGG pathways mentioned above to the STRING website (Figure 5). On the basis of the aforementioned analysis, we found the following upregulated genes (i.e., hub genes with deeper color): *AKT3*; the BCL2 apoptosis regulator gene (*BCL2*); the phosphatidylinositol-4,5-bisphosphate 3-kinase catalytic subunit alpha gene (*PIK3CA*); the phospholipase C beta 4 gene (*PLCB4*); kirsten rat sarcoma viral oncogene (*KRAS*); and *FGF6*.

### Validation of quantitative real-time reverse-transcription polymerase chain reaction

To validate the accuracy of our RNA-Seq data for DEGs in the CON and HAMR group LT samples, we randomly selected 17 DEGs (namely *AKT3, FGF6, EGFR, KRAS, CERS4, MYOD1, HSPA6, ITGA1, ITGA9, ITGA6, MAP3K20, MAP3K7, LAMA3, COL6A5, MYLK4, CACNG4*, and *DUSP5*) and quantified their expression patterns through qRT-PCR. The results demonstrated similar downregulated or upregulated trends in the expression of these genes (Supplement 5). Consequently, the qRT-PCR results confirmed the reliability of our RNA-Seq data.

## DISCUSSION

In the present study, AMRP treatment had little effect on Angus calves’ growth performance; nevertheless, it increased their LT area and slaughter rate. The color of beef is mainly determined by the amount of muscle pigment (i.e., myoglobin) and its pH [16]. Dietary AMRP supplementation can increase meat pH_24h_ significantly; nevertheless, the pH_24h_ value remains within the normal range (<5.6) [17], and the pH increase has no significant effect on meat color. In the current study, Angus calves fed with the AMRP-supplemented basal diet had lower cooking losses and shear force and tended to have elevated IMF content than those on the basal diet alone. In the case of beef, consumer acceptance, satisfaction, and willingness to purchase have been considered to depend on the meat’s tenderness. However, recent studies have indicated that consumers are increasingly indicating flavor as the most critical attribute of beef. Moreover, IMF content is positively correlated with beef tenderness and flavor. In beef, marbling is determined by the IMF deposition process, whereby fat accumulates in muscles mainly through intramuscular cell proliferation and hypertrophy during the animal’s lifetime. Harris et al [18] noted that IMF cell proliferation in the fetal and neonatal stages is critical because it provides a site for IMF cell hypertrophy in the later stages. Therefore, dietary supplementation with AMRP can improve meat quality. In the current study, we investigated the underlying mechanisms by identifying the relevant major regulatory genes and signaling pathways. Long-term muscle development and metabolic processes regulated by core genes and signaling were noted to determine meat quality.

Myogenesis is an extremely complex physiological process, which comprises three stages: (1) mesodermal mesenchymal stem cells undergo terminal differentiation to form myoblasts, (2) adult myoblasts undergo fusion to form multinucleated myotubes, and (3) myotubes undergo redifferentiation to form primary and secondary myofibers and ultimately functional mature myofibers. Skeletal muscle mass largely depends on the number and size of muscle fibers, which are heterogeneous due to differences in morphology (i.e., size) and physiological (i.e., contractile and metabolic) properties. In animals, the muscle fiber number is fixed before birth (during somatic, embryonic, and fetal stages), whereas muscle mass increases mainly through the proliferative growth of adult muscle cells. After birth, the cell proliferation rate decreases, leading to a preponderance of hypertrophy and remodeling of preexisting muscle fibers. In the present study, many genes associated with muscle development including *AKT3*, *FGF6*, *EGFR*, *KRAS*, and *CERS4* were upregulated.

*AKT3* is involved in various biological processes, primarily in response to stimulation by platelet-derived growth factor, insulin, and insulin-like growth factor. *AKT3* promotes an increase in mammalian cell size by stimulating protein synthesis and inhibiting protein degradation. Moreover, cell volume increases are largely attributable to the PI3K/AKT pathway, which regulates protein synthesis through its mTOR regulation ability. Zhu et al [2] also demonstrated that the PI3K/AKT pathway mediates growth factor signaling, engages in myofibroblast differentiation, protein synthesis, muscle hypertrophy and lipid metabolism, and inhibits protein degradation. In the current study, we found a trend of PI3K/AKT pathway upregulation (p = 0.073; Supplement 3). Moreover, in our PPI network analysis, AKT3 simultaneously appeared in six KEGG-annotated pathways, and it was at the core of our PPI network map. Therefore, AKT3 may be a crucial muscle development regulator in beef cattle.

*FGF6*, an FGF family member, acts on myogenesis by binding to tyrosine kinase receptors (i.e., FGF receptors 1 and 4); it is predominantly expressed in skeletal muscle tissue [19]. EGFR, a glycoprotein, is a tyrosine kinase–type receptor for EGF and facilitates cell proliferation and signaling; it is involved in skeletal muscle regeneration and regenerative repair processes [20]. As a member of the *RAS* gene family, *KRAS* is activated to trigger a cascade of serine/threonine kinases, which regulate cell division, proliferation, or apoptosis. Notably, in the current study, *KRAS* was enriched in multiple KEGG-annotated pathways (ko04071, ko04550, ko04010, ko04072, and ko04725). This result suggested that *KRAS* is required during the signaling process of the active ingredient of AMRP in inducing the adaptive response of Angus calves; moreover, *KRAS* specifically regulates its downstream genes to influence the developmental trends of the muscle, leading to different metabolic phenotypes. Therefore, dietary AMRP supplementation could effectively upregulate skeletal muscle stem cell–related genes; this has major implications for muscle development and skeletal muscle regeneration. These results are consistent with the large LT area, along with the trend of the high HCWs and slaughter rates, observed in our AMRP group.

We also observed that our AMRP group possessed a high water-holding capacity (WHC), as evidenced by relatively low cooking loss, possibly related to *CERS4* upregulation in muscle tissues. Ceramides are present in all eukaryotic cells, and they are not only crucial components of eukaryotic cell membranes but also secondary messengers in cellular signaling processes. Ceramides can strongly bind water molecules, maintaining cellular hydration through meshwork formation in the stratum corneum [21]. This is possibly the main reason underlying AMRP-mediated improvements in the muscle WHC. In de novo ceramide synthesis, CERS converts dihydrosphingosine to dihydroceramide, after which dihydroceramide is converted to ceramide by dihydroceramide desaturase. Thus, CERS upregulation can aid muscle cells in synthesizing ceramide and maintaining cellular integrity. Moreover, in our AMRP group, pH_24h_ increased with aging, whereas it decreased in our CON group. We also observed that the main reason for the decrease in meat WHC was lactic acid formation in the carcass during aging. Furthermore, meat WHC increases when muscle fibers hold more water molecules between filaments. This increase occurs because proteins have a negative overall charge when pH is higher than the isoelectric point (IP range = 5.2 to 5.3), which leads to filament repulsion, leaving a larger space for water molecules. Taken together, these factors explain the decrease in cooking loss noted in our AMRP group (pH = 5.49>IP).

In the current study, dietary AMRP supplementation significantly reduced *MYOD1* expression. *MYOD1*, *MYF5*, and *MYF6* (*MYF4*) are considered muscle determinants. *MYOD1* plays a role in shaping the assembly of condition-specific enhancers for muscle differentiation through transcription factor and histone-modifying enzyme recruitment [22]. Interactions between myogenic and adipocytes are believed to be critical in adipogenesis, musculogenesis, and adipogenesis–lipolysis rates and extent. Two key mechanisms underlying this interaction are secretion of adipokines (e.g., leptin) by adipocytes and that of muscle factors (e.g., muscle growth inhibitor) by myofibers. Thus, IMF deposition may be driven by a combination of cross-talk mechanisms between adipocytes and muscle cells. Dietze et al [23] established a system for coculturing human adipocytes with skeletal muscle cells and reported that myogenic cells cocultured with adipocytes and skeletal muscle cells demonstrated a reduction in insulin-stimulated AKT kinase activation compared with those cocultured with skeletal muscle cells alone; this result confirmed the presence of cross-talk between adipocytes and myocytes. In the present study, IMF content increased in LT. Therefore, we hypothesize that in Angus calves, dietary AMRP supplementation affects the balance between triglyceride uptake, synthesis, and degradation, thus inducing adipocyte development through multiple metabolic pathways and interaction with muscle cells during cellular hypertrophy, but *MYOD1* expression is downregulated. Adipocytes and muscle cells compete for or prioritize nutrient uptake and metabolism: During the initial stages after birth, the muscle growth rate is greater than the IMF deposition rate; however, IMF deposition begins to take precedence at a certain stage of growth and then increases gradually. This corroborates the current results. Guo et al [24] cocultured skeletal muscle satellite cells (MSCs) and intramuscular preadipocytes (IMPAs). The authors noted that *MYOD1* expression, related to muscle development, was significantly downregulated in MSCs; in contrast, lipid deposition–related gene expression was significantly upregulated. These results confirmed that IMPAs hinder MSC differentiation but promote lipid deposition.

AMRP may also induce the differentiation of MSCs to adipocyte-like cells. MSCs are pluripotent myogenic stem cells with the potential to transdifferentiate into osteoblasts, adipocyte-like cells, or neuronal cells; *MYOD1*, the transcriptional and myogenic regulator of *MYOD*, plays a crucial role during differentiation. Because myoblasts and adipocytes originate from the same mesoderm, myoblasts can be induced to directly transform into adipocytes. Thus, we speculated that AMRP supplementation accelerates IMF deposition by initiating *MYOD1* inhibition later in the growth phase and encouraging MSC lipogenic differentiation. *KRAS* inhibits myogenic differentiation in a manner dependent on *MYOD1* expression–based deletion. *KRAS* is an important member of the RAS superfamily [25]; therefore, AMRP may inhibit *MYOD1* by upregulating *KRAS* expression and promote lipogenic differentiation of LT myoblasts; however, because of the significant upregulation of many genes involved in muscle growth and development, it does not prevent muscle growth and development completely. This result may clarify the reason underlying the LT area being larger.

Our KEGG pathway enrichment analysis demonstrated that many DEGs were involved in the focal adhesion pathways. Focal adhesion molecules represent a cell adhesion molecule type. Cell adhesion molecules are macromolecules located on the cell membrane, which is the point of contact between a cell and its surroundings. The molecules mainly connect the actin cytoskeleton and integrins and establish a relationship with the extracellular matrix (ECM) [26]. Focal adhesion molecules demonstrate strong attachment to the ECM and transmit mechanical tension generated within the cell through the plasma membrane to the external environment; they also form a platform for the assembly of many signaling molecules during signal transduction [26]. In our study, the ECM–receptor interaction pathway was significantly upregulated (p = 0.095; Supplement 3). The ECM is a dynamic component of the cell microenvironment, and ECM-cell interactions not only provide mechanical support to cells but also play a crucial role in maintaining cellular and tissue homeostasis. Di Caprio & Bellas [27] confirmed that myogenin expression is insufficient to effectively drive skeletal muscle formation and that the presence of the ECM and its induction of cellular receptor signaling (probably through the integrin family proteins) is essential. Thus, the ECM plays an important role in completing the skeletal muscle differentiation process. In the current study, genes encoding integrin subunit alpha 1 (*ITGA1*), *ITGA9*, and i*ITGA6* were noted to be DEGs. *ITGA1*, *ITGA6*, and *ITGA9* are the members of the integrin family—which is the largest ECM-binding receptor family. Integrins are central regulators that deliver microenvironmental signals to epithelial cells for the regulation of epithelial cell polarization and morphogenesis. The proteins can mediate signaling between cells and the ECM, influencing skeletal muscle growth and development.

Greenberg et al [28] suggested that focal adhesion can activate the signal transduction pathways involving extracellular regulatory protein kinases through the MAPK signaling pathway, in turn enhancing hormone-sensitive lipase activity, increasing serum-free fatty acid concentrations, and promoting fat deposition. Focal adhesion kinase (FAK) is a nonreceptor tyrosine protein kinase with a critical role in intracellular signaling, which involves an intersection of multiple intracellular pathways and is closely related to the regulation of biological processes, including cell migration, proliferation, and apoptosis. FAK is phosphorylated at the Try^925^ site to appear as a Grb^2^-binding site and recruits Grb^2^/SOS complex formation; this is a mechanism through which FAK activates the RAS/MAPK pathway. FAK contains a binding site for p^130^ CAS junction proteins, and p^130^ CAS tyrosine phosphorylation leads to the aggregation of Crk and Nck junction proteins because these proteins can bind to SOS; this is another mechanism through which FAK activates the RAS/MAPK pathway [29]. In the present study, the expression of mitogen-activated protein kinase kinase kinase 20 (*MAP3K20*) and *MAP3K7* was significantly upregulated in the MAPK pathway. Notably, genes encoding laminin subunit alpha 3 (*LAMA3*) and collagen alpha-5(VI) chain isoform X1 (*COL6A5*) were enriched in both the focal adhesion pathway and the ECM–receptor interaction pathway in this study. ECM–receptor interactions not only promote developmental processes in skeletal muscles but also provide structural support for IMF deposition and regulate adipose tissue formation. In a study on fat deposition in different tail types of sheep, significant enrichment of genes encoding proteins involved ECM–receptor interaction processes, such as collagen VI genes (e.g., *COL6A5*), laminin genes (e.g., *LAMB3*, *LAMB4* and *LAMA2*), and integrin genes (e.g., *ITGA5*, *ITGA9*, and *ITGA1*), were found to be differentially expressed between two sheep breeds, and the DEGs were associated with fat deposition and fatty acid metabolism [30]. Adipocytes are mechanically supported by the ECM. In adipose tissues, an ever-changing ECM envelops adipocytes, allowing for remodeling during fluctuations in metabolism. Thus, we speculate that AMRP-induced IMF deposition also involves ECM–receptor interaction pathways. Our results for meat quality based on LT demonstrated that dietary AMRP supplementation may increase LT IMF but significantly reduce LT shear force. An increase in IMF content somewhat improves meat tenderness [5].

The intramuscular connective tissue, composed of ECM macromolecules, is another crucial component influencing meat tenderness; this tissue, which gradually develops when animals enter the later stages of growth contributes to an increase in muscle toughness. However, when animals enter the later stages of fattening, the IMF deposition gradually takes precedence, which results in ECM remodeling; this leads to a reduction in mechanical strength and promotes tenderness in LT [26]. Adipose tissue exhibits a dynamic ECM to accommodate the size of adipocytes, with collagen VI as a highly enriched ECM component. Di Caprio & Bellas [27] noted ECM remodeling due to collagen VI gene upregulation can limit adipocyte accumulation. In the current study, we noted upregulation of the myosin light chain kinase family member 4 gene (*MYLK4*), which is involved in the adhesion patch signaling pathway; this gene is predominantly expressed in skeletal muscle and constitutes the major myofibrillar protein in muscle cells [31]. *MYLK4*, responsible for myosin light chain phosphorylation, plays a crucial role in insulin-stimulated glucose transport in adipocytes [32]. Glucose is the main substrate used for fatty acid, glycerol, and ATP syntheses in adipocytes; therefore, *MYLK4* may affect IMF synthesis. In the present study, *ITGA1*, *ITGA9*, *ITGA6*, *MAP3K20*, MAP3K7, *LAMA3*, *COL6A5* and *MYLK4* upregulation may affect the MAPK pathway and ECM–receptor interaction signaling by affecting the adhesive patches. This may be the reason that LT tended to demonstrate high levels of IMF deposition during development. We previously also confirmed that an aqueous extract of AMRP does not regulate lipid metabolism directly, but it affects the upstream regulatory pathways of lipid metabolism–energy metabolism, signaling pathways, and cell proliferation–related processes through the modulation of changes in long noncoding RNAs and methylation [33].

Numerous studies based on transcriptome analyses have also validated the roles of the MAPK pathway in animal muscle development and expansion. This pathway is a major mechanism underlying the use of growth factors in cellular processes such as proliferation and differentiation. The MAPK pathway is responsible for transducing extracellular signals to intracellular targets in various cell types, including skeletal muscle cells, and is implicated in muscle cell proliferation (hypertrophy), growth, migration, differentiation, and apoptosis [34]. In the current study, the MAPK pathway was significantly enriched; moreover, the pathway was enriched to the highest number of differential genes (n = 26). These results suggested that dietary AMRP supplementation can promote muscle development through MAPK pathway regulation. An in vitro experimental study noted that resveratrol strongly affects various cellular components and processes, consistent with our GO analysis results of cellular component and molecular function categories. A KEGG enrichment analysis demonstrated that resveratrol promotes muscle cell proliferation, differentiation, and migration through the mediation of various signaling pathways such as MAPK and affects cell–ECM contact and actin cytoskeleton function. Resveratrol, a nonflavonoid polyphenolic organic compound, was previously studied in *A. mongolicum* powder, which also contains some nonflavonoid polyphenolic compounds such as tannins, catechins, and anthocyanins [7]. Therefore, resveratrol and the aforementioned active ingredients may demonstrate conformational similarity. It may influence the cellular components involved in biological processes and molecular functions and regulate the MAPK pathway, thus impacting muscle growth.

*HSPA6* is an HSP family member. HSP family proteins are molecular chaperone proteins that contribute to protein folding, assembly, and shipping; they also can prevent protein aggregation and degradation in cells; this allows the cells to respond defensively to heat stress and other environmental damage [35]. Many studies have recently confirmed a correlation between HSP expression and beef tenderness; as such, HSP expression is a potential biomolecular marker for myogenic fiber degradation and meat tenderness. Meat tenderization is a complex biological process involving the destruction of key myofibrillar proteins by endogenous protein hydrolases such as calpain, which are responsible for maintaining the structural integrity of myofibrils. *HSPA6*, a small HSP (sHSP), represents a class of heat stress proteins negatively correlated with tenderness. *HSPA6* can stabilize myofibril structure and decelerate apoptosis through interactions with myofibrillar proteins or endogenous protein hydrolases, thereby affecting tenderness [36]. *HSPA6* expression was noted to be downregulated in this study. We, therefore, hypothesize that AMRP reduces the heat stress response in beef cattle; this is because in our study, the temperature in July was extremely high, reaching 41.4°C; the temperature and humidity index was >72 after May 31, and it continued to be >72 until the end of the fattening period. Therefore, during the late fattening period, our calves were under heat stress. Plant polyphenols may reduce heat stress in calves by binding to mammalian transient receptor potential channels (i.e., TRPV1), which activate intracellular pathways and modulate anti-inflammatory and oxidative stress responses in tissues.

Contreras-Castillo et al [37] reported that sHSPs can act as an alternative substrate for μ-calpain and lead to reduced softening in meat samples. Thus, *HSPA6* downregulation can reduce the HSPA6–μ-calpain interaction risk without hindering the process of muscle tenderization. We also noted that the genes encoding calcium voltage-gated channel auxiliary subunit gamma 7 (*CACNG7*), *CACNG4*, and dual specificity protein phosphatase 5 (*DUSP5*) are DEGs enriched in the MAPK pathway, as well as muscle growth and development processes. Notably, a study demonstrated that aqueous extracts of AMRP can regulate differential expression of *CACNG1* and *DUSP13* and reduce the functional difference between muscle and fat tissues while maintaining their specificity—resulting in increased compatibility between the two tissues, constituting tissue harmony, and ultimately leading to improved meat quality and flavor.

The transcriptomic changes induced by AMRP supplementation were also highly enriched in the KEGG pathways associated with circadian rhythms, the PLD pathway, and the phosphatidylinositol signaling system. More than 2,300 genes in skeletal muscle expressed in a circadian pattern are involved in a wide range of functions, including muscle formation, transcription, and metabolism; moreover, in skeletal muscles, circadian rhythms are critical to muscle and systemic health. Recent studies have reported that skeletal muscle is a main target organ of the biological clock and that muscle growth, maintenance, and contractile properties exhibit strong time-dependent oscillations [38]. Circadian rhythms are generated by transcriptional and translational feedback loops of core clock genes, including the genes encoding nuclear receptor subfamily 1 group D member 1 (*NR1D1*), clock circadian regulator (*CLOCK*), cryptochrome circadian regulator 2 (*CRY2*), and RAR-related orphan receptor A (*RORA*). *CLOCK*, the core molecular clock gene, is key to skeletal muscle health. *CLOCK*-mutant mice demonstrate a 30% reduction in maximal force at the muscle and single fiber levels, disruption of myofiber structure, and a reduction in mitochondrial volume. Thus, proteins encoded by *CLOCK* are key to mitochondrial maintenance in skeletal muscle [39]. Loss of circadian core genes, such as brain and muscle ARNT-like-1 and *CLOCK*, leads to structural disorders in skeletal muscle fibers, resulting in myofibrillar lesions affecting mitochondrial function and muscle mass and strength [40]. Zhang et al demonstrated that *MYOD1* is a major regulator of muscle gene expression under the direct control of *CLOCK*; this is a crucial link between the molecular clock mechanism and the highly conserved muscle spectral transcription factors MYOD1 and myogenin [41]. The PLD pathway is a crucial secondary signaling system in cells. PLD can catalyze the hydrolysis of phosphatidylcholine to produce phosphatidic acid (PA) and choline; PA can be used as an active substrate for the PI3K/AKT and MAPK pathways, which are involved in cell differentiation, proliferation, and migratory movement. In the phosphatidylinositol system, an essential secondary cellular signaling system, phosphatidylinositol metabolites include phosphatidylinositol bisphosphate (PIP2), phosphatidylinositol triphosphate (PIP3), and diacylglycerol. The phosphatidylinositol signaling system is broadly divided into two pathways, namely the PIP2/Ca^2+^/GAG and PIP3/AKT pathways; these pathways regulate activities of multiple enzymes and initiate a series of cascading reactions involved in cell growth, differentiation, and apoptosis. The transcriptome analysis results provide novel insights into the genetic control of traits, and the identified genes may be used to improve carcass and muscle development traits. Our findings indicating the key genes and biological processes promoting muscle growth and IMF deposition may aid in improving Angus cattle breeding and production processes. Moreover, elucidating their mechanisms may facilitate the creation of newer, enhanced measures for optimizing the rational application of natural plant-derived feed additives in animal husbandry.

In general, dietary AMRP supplementation affected the LT transcriptome. However, this study has some limitations: (*i*) single dosing level, (*ii*) short fattening time, and (*iii*) small sample. Therefore, future studies should assess different AMRP dosage gradients to explore the dose–response relationships, understand the long-term effects of AMRP supplementation on beef cattle growth and muscle development, and relate them to other dietary factors. AMRP has a positive effect on the growth of ruminants, and the modulation of the intestinal microflora structure is considered one of the most important effects of AMRP. In contrast, the physiological mechanisms through which the active ingredients in AMRP affect the skeletal muscle and adipose tissue in animals are unclear. Future studies should focus on elucidating the mechanisms through which circadian regulation affects muscle development, directly investigating the intrinsic association between fat deposition and muscle development, and determining whether active ingredients effectively manipulate different types of muscle fibers. The combined effects of the environment, rumen microbiome, epigenome, and their interactions determine muscle development in beef cattle. Therefore, in the future, a multiomic-based systems approach should be used to develop an integrated solution to improve the economic traits of beef cattle, including growth and meat quality.

## CONCLUSION

In this study, dietary AMRP supplementation was noted to increase the LT area and IMF content in black Angus calves. In the transcriptome analysis, dietary AMRP supplementation was observed to upregulate 1,002 genes and downregulate 282 genes in LT, respectively. DEGs were significantly enriched in the GO terms in the molecular function and cellular component categories. Moreover, the KEGG pathway enrichment analysis demonstrated that AMRP supplementation activated the focal adhesion and MAPK pathways, which may promote muscle growth and IMF deposition, thus improving meat quality. In general, these results indicated that AMRP may be used as a source of natural bioactive compounds in the calves’ diet to improve meat yield and quality and facilitate the selection of appropriate dietary supplements that guarantee breeder and consumer satisfaction in the beef industry.

## Figures and Tables

**Table 1:** GenBank accession numbers and primer sequences for qRT-PCR

Gene name^1^	Accession number	Primer sequence	Length (bp)	Annealing temperature (°C)
*GAPDH*	NC_019460.2	F: 5′-GAGAAACCTGCCAAGTATGA-3′ R: 5′-TACCAGGAAATGAGCTTGAC-3	203	62
*β-Actin*	NC_019471.2	F: 5′-GAAAACGAGATGAGATTGGC-3′ R: 5′-CCATCATAGAGTGGAGTTCG-3	194	62
*AKT3*	XM_024975971.2	F: 5′-ACTAGAGAGTGGGAAGGGCA-3′ R: 5′-ATATATTCTCCCGCCCGCCA-3′	222	61
*FGF6*	NM_001192400.1	F: 5′-GGAAAATTATACGCCACGCCC-3′ R: 5′-GCTTCACCCTTCCGTATTTGC-3′	139	60
*EGFR*	XM_002696890.6	F: 5′-AAAAGTGTGACGGGCCTTGT-3′ R: 5′-AGGTGCAGTACGTGTGAAGG-3′	179	61
*KRAS*	XM_024992102.2	F:5′-TACATGAGGACTGGGGAGGG-3′ R:5′-AGTCCTGAGCCTGTTTTGTGT-3′	187	60
*CERS4*	XM_005208850.4	F:5′-AAGCGCAAGGACTTCAAGGA-3′ R:5′-CCGAAGAAGGGGCTGAAGTT-3′	299	60
*MYOD1*	NM_001040478.2	F:5′-GCACGTCTAGCAACCCAAAC-3′ R:5′-GCTGTAGTAAGTGCGGTCGT-3′	293	60
*HSPA6*	XM_002685850.6	F: 5′-GGTGGAGAGGATGGTTCGTG-3′ R: 5′-TTGTACTTTGCGCCTGTCCT-3′	172	60
*ITGA1*	XM_059878883.1	F: 5′-GGCTCCTCTCCGTTGTTCTG-3′ R:5′-AGCACCCATTTCCCTTCTTCG-3′	139	60
*ITGA9*	NM_001192718.2	F: 5′-ACACTTTGGGGAGAGCATCG-3′ R: 5′-TCCGACATGAAGGCTCCAAC-3′	270	60
*ITGA6*	NM_001109981.2	F: 5′-TGGGCTGTCGTCAAGAGTTC-3′ R: 5′-TTCTTCCATGCACGCCTTCT-3′	103	60
*MAP3K20*	NM_001205493.1	F: 5′-GGTTTCTCGGTCAGCACTCA-3′ R:5′-CTCCACCTTTGTCCATCCCC-3′	169	60
*MAP3K7*	NM_001081595.1	F: 5′-ATGCGGTACTTTCCAGGAGC-3′ R:5′-CCACGAGAAGCTCCCAAACT-3′	257	60
*LAMA3*	XM_024984437.2	F: 5′-CTGCACCCGCCCTACTTCAA-3′ R: 5′-GTTGTACTGCATGCCTGACG-3′	279	61
*COL6A5*	XM_024997170.2	F: 5′-CGCAAAGATGGGGTGAGGAT-3′ R: 5′-ATTCTCTGTGGTCCCAACGG-3′	279	60
*MYLK4*	XM_010818635.4	F: 5′-TGAAGGACAAGGACGACGTG-3′ R: 5′-TGTCCCGATTCACACACAGG-3′	294	60
*CACNG4*	XM_002696227.6	F: 5′-CCATGACAGCTCGGAGTACC-3′ R:5′-TCTTGCGGCTGTAGAACCTG-3′	125	60
*DUSP5*	NM_001304282.2	F:5′-GGGCGGGAAAGAAGAGTTGA-3′ R:5′-TTGAGGTTGACGTTCAGCGA-3′	293	60

qRT-PCR, quantitative real-time reverse-transcription polymerase chain reaction; GADPH, glyceraldehyde-3-phosphate dehydrogenase; AKT3, AKT serine/threonine kinase 3; FGF6, fibroblast growth factor 6; EGFR, epidermal growth factor receptor; KRAS, kirsten rat sarcoma viral oncogene; CERS4, ceramide synthase 4; MYOD1, myogenic differentiation 1; HSPA6, heat shock protein family A member 6; ITGA1, integrin subunit alpha 1; ITGA9, integrin subunit alpha 9; ITGA6, integrin subunit alpha 6; MAP3K20, mitogen-activated protein kinase kinase kinase 20; MAP3K7, mitogen-activated protein kinase kinase kinase 7; LAMA3, laminin subunit alpha 3; COL6A5, collagen alpha-5(VI) chain isoform X1; MYLK4, myosin light chain kinase family member 4; CACNG4, calcium voltage-dependent calcium channel gamma-4 subunit; DUSP5, dual specificity protein phosphatase 5.

**Table 2:** Effects of dietary AMRP supplementation on growth performance, carcass characteristics, and meat quality of black Angus calves

Items	CON^1)^	AMRP	SEM	p-value
Growth performance				
Initial body weight (kg)	274.67	266.83	15.74	0.521
Final body weight (kg)	376.00	381.00	14.87	0.668
Average daily intake (kg)	8.70	8.64	0.19	0.159
Average daily gain (kg)	0.79	0.86	0.10	0.294
Preslaughter weight (kg)	387.17	384.33	14.99	0.593
Carcass characteristics				
Hot carcass weight (kg)	193.80	199.86	6.714	0.072
Dressing percentage (%)	50.31	51.87	0.945	0.078
LT area (cm^2^)	80.27	84.70	0.83	0.040
Meat quality				
pH_45min_	6.45	6.51	0.07	0.583
pH_24h_	5.29	5.49	0.05	0.002
L*	49.9	48.2	0.23	0.256
a*	15.2	14.9	0.32	0.675
b*	22.2	22.5	0.11	0.523
WBSF (N)	75.26	48.23	3.99	0.009
Drip loss (%)	5.12	5.32	0.68	0.184
Cooking loss (%)	53.52	46.79	2.58	<0.001
Intramuscular fat (%)	2.03	2.56	0.53	0.057

1) CON, basal diet without additives; AMRP, basal diet + 20 g of dried AMRP per calf per day.

AMRP, *Allium mongolicum* Regel powder; SEM, standard error of the mean; LT, longissimus thoracis; WBSF, Warner-Bratzler shear force.

**Table 3:** Comparison of each sample and reference genomic information mapping statistics^1)^

Sample	Clean reads	Unmapped reads	Total mapped reads	Uniquely mapped reads	Multiple mapped reads
CON 1	46,881,792	46,643,316 (99.49%)	45,030,357 (96.54%)	42,927,228 (92.03%)	2,103,129 (4.51%)
CON 2	42,553,048	42,298,252 (99.40%)	40,917,698 (96.74%)	39,200,604 (92.68%)	1,717,094 (4.06%)
CON 3	49,690,700	49,464,554 (99.54%)	47,618,288 (96.27%)	45,504,861 (91.99%)	2,113,427 (4.27%)
CON 4	39,372,842	39,138,000 (99.40%)	37,698,416 (96.32%)	36,112,685 (92.27%)	1,585,731 (4.05%)
CON 5	39,944,324	39,707,312 (99.41%)	38,224,185 (96.26%)	36,687,034 (92.39%)	1,537,151 (3.87%)
AMRP 1	47,824,064	47,527,944 (99.38%)	45,769,287 (96.30%)	43,769,532 (92.09%)	1,999,755 (4.21%)
AMRP 2	46,292,564	45,986,400 (99.34%)	44,327,589 (96.39%)	42,388,289 (92.18%)	1,939,300 (4.22%)
AMRP 3	43,715,720	43,430,132 (99.35%)	42,016,691 (96.75%)	40,169,285 (92.49%)	1,847,406 (4.25%)
AMRP 4	42,824,892	42,593,764 (99.46%)	41,167,190 (96.65%)	39,332,191 (92.34%)	1,834.999 (4.31%)
AMRP 5	43,512,288	43,266,898 (99.44%)	41,673,498 (96.32%)	39,910,996 (92.24%)	1,762,502 (4.07%)

1) Five replicates of the CON group (CON 1–5) and the AMRP group (AMRP 1–5) were in the transcriptome analysis.

AMRP, *Allium mongolicum* Regel powder.

**Table 4:** DEGs enriched in signal transduction pathways

Pathway	p-value	Total DEGs	Downregulated DEGs	Upregulated DEGs
Sphingolipid pathway	0.001	15	PPP2R5A	PIK3R3; MAPK9; ROCK2; PIK3CA; KRAS; GNAQ; SGMS1; PLCB4; SPTLC3; AKT3; BCL2; PPP2R5E; PPP2R3; CERS4
Focal adhesion	0.005	20	FLT4; RELN	THBS1; PIK3R3; COMP; MAPK9 ROCK2; PIK3CA; EGFR; ITGA1 ITGA9; FLT1; ITGA6; AKT3; BCL2 BRAF; LAMA3; CAV2; MYLK4
Adherens junction	0.007	10	YES1	MAP3K7; SORBS1; FER; EGFR; IQGAP1; PTPRB; SSX2IP; pol; Zo-1
Signaling pathways regulating stem cell pluripotency	0.008	15	-	PIK3R3; SKIL; PCGF5; BMPR2; PIK3CA; KRAS; PCGF6; LIFR; FZD6; IL6ST; AKT3; RIF1; TBX3 SMAD5; Rx1
MAPK pathway	0.009	26	FLT4; HSPAIL; HSPA6	TAOK1; MAP3K20; MAP3K7; MAPK9 FGF6; RASGRP3 KRAS; EGFR; NF1; RAPGEF2; TAOK3; PPP3CA; FLT1; KITLG; RPS6KA3; AKT3; PPM1A CACNA2D1; BRAF; RASGRF2; CACNG7; DUSP5; CACNG4
Circadian rhythm	0.012	6	NR1D1; RORC; CRY2	PRKAA1; RORA; CLOCK
PLD pathway	0.022	16	PIK3R5; AVPRIA; AGT; PTPN11	PIK3R3; AGPAT5; LPAR6; PIK3CA; KRAS; EGFR; PLCB4; KITLG; AKT3; RALB; IGHA2; IGHG2
Cholinergic synapse	0.037	11	PIK3R5	ACHE; ITPR2; PIK3R3; GNG10; PIK3CA; KRAS; GNAQ; PLCB4; AKT3; BCL2
Phosphatidylinositol signaling system	0.039	10	-	PIKFYVE; ITPR2; PIK3R3; PIK3C2A; PIK3CA; MTMR7; IPMK PLCB4; INPP4B; MTMR2

DEGs, differentially expressed genes; PLD, phospholipase D.

## SUPPLEMENTARY MATERIAL

**Supplement 1**. GenBank accession numbers and primer sequences for qRT-PCR

**Supplement 2**. Differentially expressed genes (DEGs) in Longissimus thoracis muscle of CON and HAMR group

**Supplement 3**. The top 20 enriched terms in GO analysis of DEGs between CON and HAMR group

**Supplement 4**. The enriched terms in KEGG pathway analysis of DEGs between CON and HAMP group

**Supplement 5**. Expression levels of 17 randomly selected differentially expressed genes by qRT-PCR.
