# Supplementary material for: Molecular regulatory mechanisms of dietary supplementation with Allium mongolicum Regel powder to improve muscle development and meat quality in Angus calves
Source: Anim Biosci. 2025 Feb 27;38(8):1798–816. doi: 10.5713/ab.24.0809 (PMC12229934; doi:10.5713/ab.24.0809)
Supplement: Supplementary file 1 [file ab-24-0809-Supplementary-1.pdf]

Supplement 1. GenBank accession numbers and primer sequences for qRT-PCR

Gene name[1]	Accession number	Primer sequence	Length, bp	Annealing Temperature, °C
GAPDH	NC_019460.2	F: 5'-GAGAAACCTGCCAAGTATGA-3'<br>R: 5'-TACCAGGAAATGAGCTTGAC-3	203	62
β-Actin	NC_019471.2	F: 5'-GAAAACGAGATGAGATTGGC-3'<br>R: 5'-CCATCATAGAGTGGAGTTCG-3	194	62
AKT3	XM_024975971.2	F: 5'-ACTAGAGAGTGGGAAGGGCA-3'<br>R: 5'-ATATATTCTCCCGCCCGCCA-3'	222	61
FGF6	NM_001192400.1	F: 5'-GGAAAATTATACGCCACGCCC-3'<br>R: 5'-GCTTCACCCTTCCGTATTTGC-3'	139	60
EGFR	XM_002696890.6	F: 5'-AAAAGTGTGACGGGCCTTGT-3'<br>R: 5'-AGGTGCAGTACGTGTGAAGG-3'	179	61
KRAS	XM_024992102.2	F:5'-TACATGAGGACTGGGGAGGG-3'<br>R:5'-AGTCCTGAGCCTGTTTTGTGT-3'	187	60
CERS4	XM_005208850.4	F:5'-AAGCGCAAGGACTTCAAGGA-3'<br>R:5'-CCGAAGAAGGGGCTGAAGTT-3'	299	60
MYOD1	NM_001040478.2	F:5'-GCACGTCTAGCAACCCAAAC-3'<br>R:5'-GCTGTAGTAAGTGCGGTCGT-3'	293	60
HSPA6	XM_002685850.6	F: 5'-GGTGGAGAGGATGGTTCGTG-3'<br>R: 5'-TTGTACTTTGCGCCTGTCCT-3'	172	60
ITGA1	XM_059878883.1	F: 5'-GGCTCCTCTCCGTTGTTCTG-3'<br>R:5'-AGCACCCATTTCCCTTCTTCG-3'	139	60
ITGA9	NM_001192718.2	F: 5'-ACACTTTGGGGAGAGCATCG-3'<br>R: 5'-TCCGACATGAAGGCTCCAAC-3'	270	60
ITGA6	NM_001109981.2	F: 5'-TGGGCTGTCGTCAAGAGTTC-3'<br>R: 5'-TTCTTCCATGCACGCCTTCT-3'	103	60
MAP3K20	NM_001205493.1	F: 5'-GGTTTCTCGGTCAGCACTCA-3'<br>R:5'-CTCCACCTTTGTCCATCCCC-3'	169	60
MAP3K7	NM_001081595.1	F: 5'-ATGCGGTACTTTCCAGGAGC-3'	257	60

		R:5'-CCACGAGAAGCTCCCAAACT-3'		
LAMA3	XM_024984437.2	F: 5'- CTGCACCCGCCCTACTTCAA-3'<br>R: 5'- GTTGTACTGCATGCCTGACG-3'	279	61
COL6A5	XM_024997170.2	F: 5'-CGCAAAGATGGGGTGAGGAT-3'<br>R: 5'-ATTCTCTGTGGTCCCAACGG-3'	279	60
MYLK4	XM_010818635.4	F: 5'-TGAAGGACAAGGACGACGTG-3'<br>R: 5'- TGTCCCGATTCACACACAGG-3'	294	60
CACNG4	XM_002696227.6	F: 5'-CCATGACAGCTCGGAGTACC-3'<br>R:5'-TCTTGCGGCTGTAGAACCTG-3'	125	60
DUSP5	NM_001304282.2	F:5'- GGGCGGGAAAGAAGAGTTGA-3'<br>R:5'-TTGAGGTTGACGTTCAGCGA-3'	293	60

[1]GADPH = glyceraldehyde-3-phosphate dehydrogenase, β-actin = beta-actin, AKT3 = AKT serine/threonine kinase 3, FGF6 = fibroblast growth factor 6, EGFR = epidermal growth factor receptor, KRAS = kirsten rat sarcoma viral oncogene, CERS4 = ceramide synthase 4, MYOD1 = Myogenic Differentiation 1, HSPA6 = heat shock protein family A (HSP70) member 6, ITGA1 = integrin subunit alpha 1, ITGA9 =integrin subunit alpha 9, ITGA6 = integrin subunit alpha 6, MAP3K20 = mitogen-activated protein kinase kinase kinase 20, MAP3K7 = mitogen-activated protein kinase kinase kinase 7), LAMA3 = laminin subunit alpha 3, COL6A5 = collagen alpha-5(VI) chain isoform X1, MYLK4 = myosin light chain kinase family member 4, CACNG4 = calcium voltage-dependent calcium channel gamma-4 subunit, DUSP5 = dual specificity protein phosphatase 5
