# Supplementary material for: Molecular regulatory mechanisms of dietary supplementation with Allium mongolicum Regel powder to improve muscle development and meat quality in Angus calves
Source: Anim Biosci. 2025 Feb 27;38(8):1798–816. doi: 10.5713/ab.24.0809 (PMC12229934; doi:10.5713/ab.24.0809)
Supplement: Supplementary file 2 [file ab-24-0809-Supplementary-2.pdf]

Supplement 2. Differentially expressed genes(DEGs) in Longissimus thoracis muscle of CON and HAMR group

Number	ID	DEGs	10g2(F01d Change)	*P*- Value	FDR	Up or Down
1	ENSBTAG00000007075	HLA-A	-3.357060554	3.14E-147	5.30E-143	up
2	ENSBTAG00000039035	HSPA6	4.437258747	1.30E-109	1.09E-105	down
3	ENSBTAG00000011976	CYP4B1	-3.25417031	2.92E-92	1.64E-88	up
4	ENSBTAG00000000392	ASCC1	1.423844986	7.45E-78	3.14E-74	down
5	ENSBTAG00000011465	MYBPH	3.51568956	6.10E-73	2.05E-69	down
6	ENSBTAG00000014831	PPP1R3C	1.275672143	5.00E-67	1.40E-63	down
7	ENSBTAG00000052397	MYOD1	5.247863704	1.22E-64	2.93E-61	down
8	ENSBTAG00000004602	PITX1	3.942801104	8.13E-61	1.71E-57	down
9	ENSBTAG00000040053	MYH6	-2.433895461	2.84E-56	5.31E-53	up
10	ENSBTAG00000037605	HLA-DQA2	-1.502139443	1.43E-54	2.41E-51	up
11	ENSBTAG00000021306	CHRDL2	2.514872043	1.31E-53	2.01E-50	down
12	ENSBTAG00000008394	MYL3	1.730609246	1.56E-50	2.19E-47	down
13	ENSBTAG00000023648	ART5	-1.492791273	5.05E-44	6.54E-41	up
14	ENSBTAG00000010452	PODXL	-1.779335372	2.79E-42	3.35E-39	up
15	ENSBTAG00000010161	CCL21	2.135034482	2.12E-41	2.37E-38	down
16	ENSBTAG00000047996	IFITM10	2.383450679	1.02E-40	1.08E-37	down
17	ENSBTAG00000021516	GSTA1	2.021152606	6.26E-40	6.21E-37	down
18	ENSBTAG00000034185	EEF1A1	4.350348578	3.78E-38	3.53E-35	down
19	ENSBTAG00000001086	FHL2	2.360672177	3.13E-37	2.77E-34	down
20	ENSBTAG00000012079	MYLK4	-1.152988235	8.85E-37	7.45E-34	up
21	ENSBTAG00000050786	SECTM1	1.837447398	2.66E-35	2.13E-32	down
22	ENSBTAG00000007090	MYH2	-1.207407782	2.84E-35	2.18E-32	up
23	ENSBTAG00000017391	HS3ST2	3.263844516	5.48E-35	4.01E-32	down
24	MSTRG.9972	--	1.363292646	1.29E-34	9.03E-32	down
25	ENSBTAG00000017328	--	1.629362539	3.20E-34	2.16E-31	down
26	ENSBTAG00000051206	KY	1.500875594	5.01E-34	3.25E-31	down
27	ENSBTAG00000012208	Patr-A	-2.21130109	8.48E-34	5.29E-31	up
28	ENSBTAG00000003152	IFI6	1.70105934	2.34E-33	1.41E-30	down
29	ENSBTAG00000023179	TRIB1	1.143222065	3.03E-32	1.76E-29	down
30	ENSBTAG00000054342	H2-T3	-3.481674386	4.28E-32	2.40E-29	up
31	ENSBTAG00000048470	IFITM1	1.272526213	5.57E-32	3.03E-29	down
32	ENSBTAG00000005744	COQ2	-1.650497144	1.26E-31	6.60E-29	up
33	MSTRG.3909	--	-1.577413357	5.85E-31	2.99E-28	up
34	ENSBTAG00000039440	COX7C	-2.631209645	1.05E-29	5.19E-27	up
35	ENSBTAG00000045757	TNNC1	1.269179581	1.66E-29	8.00E-27	down
36	ENSBTAG00000011193	C1QC	1.456893071	1.74E-29	8.15E-27	down
37	ENSBTAG00000012178	NR1D1	1.515826495	2.29E-29	1.04E-26	down
38	ENSBTAG00000027962	SDHC	-9.214516629	3.41E-29	1.51E-26	up
39	ENSBTAG00000016768	SCN3B	-1.471614818	4.29E-29	1.85E-26	up
40	ENSBTAG00000021902	ALDH5A1	1.339075952	1.30E-28	5.48E-26	down
41	ENSBTAG00000007415	SLC7A8	-1.02809473	1.68E-27	6.90E-25	up

42	ENSBTAG00000015406	ZNF750	1.577194514	4.59E-27	1.84E-24	down
43	ENSBTAG00000004347	ADGRF5	-1.54554032	7.78E-27	3.05E-24	up
44	ENSBTAG00000008763	PRR32	2.443634889	1.83E-25	6.85E-23	down
45	ENSBTAG00000027181	LAMA3	-1.877240107	6.63E-25	2.43E-22	up
46	ENSBTAG00000016448	ZBTB40	-1.214089118	5.35E-24	1.88E-21	up
47	ENSBTAG00000046158	CFB	2.093297379	7.52E-24	2.53E-21	down
48	ENSBTAG00000002006	THBS1	-1.356263806	8.93E-24	2.95E-21	up
49	ENSBTAG00000016915	FLT1	-1.236310265	1.29E-23	4.19E-21	up
50	ENSBTAG00000012302	RTN4RL1	1.105795491	4.34E-23	1.38E-20	down
51	ENSBTAG00000019018	IFITM3	14.15515027	6.82E-23	2.13E-20	down
52	ENSBTAG00000007554	IFI6	1.433329013	7.32E-23	2.24E-20	down
53	ENSBTAG00000021396	HEG1	-1.353934732	2.26E-22	6.81E-20	up
54	ENSBTAG00000008401	PFKFB3	1.165795786	2.83E-22	8.37E-20	down
55	ENSBTAG00000004950	BRB	1.178610534	3.52E-22	1.02E-19	down
56	ENSBTAG00000011390	CHRND	1.822913589	4.79E-22	1.37E-19	down
57	ENSBTAG00000014313	PYROXD2	1.520544353	6.20E-22	1.74E-19	down
58	ENSBTAG00000002551	KCNJ8	1.058509728	6.29E-22	1.74E-19	down
59	ENSBTAG00000051907	CYBA	1.405333528	1.42E-21	3.86E-19	down
60	ENSBTAG00000016063	DNER	8.019892844	1.81E-21	4.83E-19	down
61	ENSBTAG00000008240	GALNT15	-1.177363221	4.63E-21	1.20E-18	up
62	ENSBTAG00000018562	LDAF1	-1.012397906	1.22E-20	3.12E-18	up
63	ENSBTAG00000005714	ACTC1	1.079976598	1.29E-20	3.23E-18	down
64	ENSBTAG00000006030	MYOG	1.142048385	1.78E-20	4.40E-18	down
65	ENSBTAG00000009656	HLA-DQA2	9.173228677	2.19E-20	5.34E-18	down
66	ENSBTAG00000019015	IFITM3	1.214130883	3.59E-20	8.52E-18	down
67	MSTRG.1649	KCNS3	1.556861281	1.21E-19	2.78E-17	down
68	ENSBTAG00000049782	--	1.70043191	1.43E-19	3.21E-17	down
69	ENSBTAG00000020056	COL12A1	-1.372791888	2.09E-19	4.64E-17	up
70	ENSBTAG00000027064	BTBD11	-1.433217515	2.40E-19	5.25E-17	up
71	ENSBTAG00000000601	COL11A2	1.601247139	2.61E-19	5.64E-17	down
72	ENSBTAG00000012314	LDLR	1.424816992	3.78E-19	8.07E-17	down
73	ENSBTAG00000008827	SPOCK2	-1.104458981	4.24E-19	8.92E-17	up
74	ENSBTAG00000016520	MCT7	1.538627545	7.98E-19	1.64E-16	down
75	ENSBTAG00000001864	NR4A3	-1.801753553	1.01E-18	2.03E-16	up
76	ENSBTAG00000011196	ClQB	1.16050479	2.97E-18	5.81E-16	down
77	ENSBTAG00000003357	CLRN2	8.688137484	4.34E-18	8.20E-16	down
78	ENSBTAG00000021223	CRY2	1.042048266	4.80E-18	8.98E-16	down
79	ENSBTAG00000003074	SLC16A6	-1.800811823	7.90E-18	1.43E-15	up
80	ENSBTAG00000018248	MGLL	1.029929912	1.19E-17	2.13E-15	down
81	MSTRG.13864	--	1.588940356	2.14E-17	3.80E-15	down
82	ENSBTAG00000014707	ISG15	1.93122226	2.27E-17	3.98E-15	down
83	MSTRG.3074	--	1.756897239	3.27E-17	5.61E-15	down
84	ENSBTAG00000007153	C1QA	1.021540471	4.12E-17	7.01E-15	down
85	ENSBTAG00000002786	MYADM	-2.94887191	4.63E-17	7.80E-15	up

86	ENSBTAG00000002996	SHROOM4	-1.078443568	4.96E-17	8.26E-15	up
87	ENSBTAG00000008182	FOSB	-1.724135991	1.65E-16	2.68E-14	up
88	ENSBTAG00000011617	MAMSTR	1.040700565	3.35E-16	5.27E-14	down
89	ENSBTAG00000017069	GASK1B	-1.960367895	7.43E-16	1.15E-13	up
90	ENSBTAG00000039319	CYP4F21	2.379134952	1.04E-15	1.56E-13	down
91	ENSBTAG00000015296	PTPRB	-1.643813357	1.13E-15	1.66E-13	up
92	ENSBTAG00000054931	CACNG4	-1.292942563	1.18E-15	1.73E-13	up
93	ENSBTAG00000017549	KITLG	-2.350640592	1.27E-15	1.84E-13	up
94	ENSBTAG00000012450	RAPGEF2	-1.167081849	1.29E-15	1.86E-13	up
95	ENSBTAG00000032051	SULT1C4	-1.176994766	1.31E-15	1.87E-13	up
96	ENSBTAG00000044073	CD248	1.107469038	1.51E-15	2.11E-13	down
97	ENSBTAG00000053886	NDUFAF8	1.003815385	1.55E-15	2.14E-13	down
98	ENSBTAG00000004238	TACC1	-1.341084525	1.57E-15	2.15E-13	up
99	MSTRG.2465	--	1.09443778	1.59E-15	2.16E-13	down
100	ENSBTAG00000003721	CHST1	1.134020881	1.78E-15	2.40E-13	down
101	ENSBTAG00000019146	OSBP2	1.148165951	2.07E-15	2.74E-13	down
102	MSTRG.2373	--	2.49205992	3.42E-15	4.49E-13	down
103	ENSBTAG00000039861	OAS1	1.045893534	3.78E-15	4.93E-13	down
104	ENSBTAG00000017405	RORC	1.104567538	3.88E-15	5.02E-13	down
105	ENSBTAG00000011500	CASQ2	1.042135456	4.25E-15	5.47E-13	down
106	ENSBTAG00000016357	VNN2	1.89438116	4.80E-15	6.13E-13	down
107	ENSBTAG00000047605	slc23a2	-1.110826149	6.56E-15	8.25E-13	up
108	ENSBTAG00000050914	CYYR1	-1.591856421	2.54E-14	2.97E-12	up
109	MSTRG.8843	--	-1.586254659	2.58E-14	3.00E-12	up
110	ENSBTAG00000031532	DACT2	1.996826387	2.78E-14	3.20E-12	down
111	ENSBTAG00000001462	FLT4	1.384263733	4.79E-14	5.31E-12	down
112	MSTRG.1187	--	-3.236694648	1.15E-13	1.26E-11	up
113	ENSBTAG00000002910	ECHDC2	1.031090805	1.38E-13	1.49E-11	down
114	ENSBTAG00000012393	AGT	1.068048563	1.50E-13	1.59E-11	down
115	ENSBTAG00000010442	PANK1	-1.621399507	1.63E-13	1.73E-11	up
116	ENSBTAG00000004078	KCNH2	1.41471896	2.77E-13	2.89E-11	down
117	ENSBTAG00000004982	GPLD1	2.794201778	5.16E-13	5.36E-11	down
118	ENSBTAG00000004908	CHRNE	1.036157867	5.57E-13	5.75E-11	down
119	ENSBTAG00000047502	FKBP5	-1.50726312	6.06E-13	6.19E-11	up
120	ENSBTAG00000020658	LNX1	-1.2980955	6.25E-13	6.27E-11	up
121	ENSBTAG00000024492	PTPN11	1.189893305	6.88E-13	6.78E-11	down
122	MSTRG.11382	--	-1.948660807	6.98E-13	6.83E-11	up
123	ENSBTAG00000016411	RNF122	1.045412271	7.11E-13	6.92E-11	down
124	ENSBTAG00000046339	VASN	1.157156245	8.06E-13	7.80E-11	down
125	ENSBTAG00000014069	PDK4	-1.303561312	9.41E-13	9.05E-11	up
126	ENSBTAG00000014124	MLLT3	-1.692850292	9.84E-13	9.42E-11	up
127	ENSBTAG00000021706	TBX3	-1.330225429	1.01E-12	9.59E-11	up
128	ENSBTAG00000051698	DCXR	-2.378983317	1.16E-12	1.10E-10	up
129	ENSBTAG00000004510	SARDH	1.282976789	1.71E-12	1.57E-10	down

130	ENSBTAG00000013650	SLC66A1	1.055933495	1.78E-12	1.61E-10	down
131	ENSBTAG00000053198	eeflas	6.791629983	2.67E-12	2.35E-10	down
132	ENSBTAG00000021911	PTPRG	-1.08963541	3.43E-12	2.96E-10	up
133	ENSBTAG00000034662	UQCRC2	5.408293412	3.61E-12	3.09E-10	down
134	ENSBTAG00000013060	IQGAP1	-1.087540666	4.94E-12	4.18E-10	up
135	ENSBTAG00000016566	ITGA9	-1.172088258	5.10E-12	4.30E-10	up
136	ENSBTAG00000004305	RGS16	1.135904647	6.04E-12	5.01E-10	down
137	ENSBTAG00000049368	Tjpl	-1.034519398	8.13E-12	6.61E-10	up
138	ENSBTAG00000048486	IQANK1	1.613014686	8.88E-12	7.15E-10	down
139	ENSBTAG00000003434	CERS4	-1.587007427	1.12E-11	8.84E-10	up
140	ENSBTAG00000017504	FAIM2	-1.519990295	1.26E-11	9.73E-10	up
141	ENSBTAG00000049589	SAA1	2.127877566	1.42E-11	1.07E-09	down
142	ENSBTAG00000000505	KYAT3	2.020288174	1.46E-11	1.10E-09	down
143	ENSBTAG00000020601	ZNF366	-1.137391994	1.49E-11	1.11E-09	up
144	ENSBTAG00000007071	RAI14	-1.366752569	2.14E-11	1.56E-09	up
145	ENSBTAG00000052165	pol	1.900824595	2.33E-11	1.68E-09	down
146	ENSBTAG00000027569	APBB2	-1.084365558	2.58E-11	1.83E-09	up
147	ENSBTAG00000014382	KANK4	-1.047673192	2.64E-11	1.85E-09	up
148	ENSBTAG00000001308	MYADM	-9.437960088	2.73E-11	1.91E-09	up
149	ENSBTAG00000012994	LOX	-1.780740234	2.94E-11	2.05E-09	up
150	ENSBTAG00000053071	RNF213	-1.260979143	4.33E-11	2.96E-09	up
151	ENSBTAG00000016991	EFNB2	-1.274870387	4.88E-11	3.30E-09	up
152	ENSBTAG00000001139	ACHE	-1.510306225	5.61E-11	3.78E-09	up
153	ENSBTAG00000030259	RASGRF2	-1.434299401	5.77E-11	3.87E-09	up
154	ENSBTAG00000002313	ITPR2	-1.94654433	6.04E-11	4.02E-09	up
155	ENSBTAG00000013773	PKP4	-1.275432216	6.33E-11	4.20E-09	up
156	ENSBTAG00000012684	CCL19	1.619337363	6.61E-11	4.37E-09	down
157	ENSBTAG00000015844	TFPI2	-1.196916108	1.11E-10	7.20E-09	up
158	ENSBTAG00000017266	ITGA6	-1.500552903	1.22E-10	7.87E-09	up
159	ENSBTAG00000004427	OSBPL8	-1.74004649	1.33E-10	8.51E-09	up
160	ENSBTAG00000011831	SPPL2A	-1.575453391	1.52E-10	9.62E-09	up
161	ENSBTAG00000002880	SORBS1	-1.089843405	1.64E-10	1.03E-08	up
162	MSTRG.13361	--	-1.509832987	2.04E-10	1.27E-08	up
163	ENSBTAG00000044195	SDK2	1.137835311	2.49E-10	1.53E-08	down
164	ENSBTAG00000019901	RAD54L	1.979306015	2.79E-10	1.71E-08	down
165	ENSBTAG00000031569	GOLM1	-1.195862038	3.85E-10	2.31E-08	up
166	ENSBTAG00000007814	WWTR1	-1.20344022	4.87E-10	2.88E-08	up
167	ENSBTAG00000011628	EGFR	-1.084569823	5.53E-10	3.20E-08	up
168	ENSBTAG00000048622	TMOD3	-1.418426563	6.13E-10	3.49E-08	up
169	ENSBTAG00000015106	DSP	1.629729399	6.53E-10	3.68E-08	down
170	ENSBTAG00000008453	LBR	-1.246896229	6.54E-10	3.68E-08	up
171	ENSBTAG00000017129	CLCC1	-1.377684495	7.39E-10	4.12E-08	up
172	MSTRG.3033	--	-3.476118628	7.84E-10	4.36E-08	up
173	ENSBTAG00000000802	LYVE1	1.340390623	8.42E-10	4.67E-08	down

174	ENSBTAG00000021684	TCEANC	10.38089392	8.74E-10	4.83E-08	down
175	ENSBTAG00000053433	MICA	-4.543646202	1.18E-09	6.41E-08	up
176	ENSBTAG00000007239	TNFAIP6	-2.301479259	1.25E-09	6.76E-08	up
177	ENSBTAG00000000202	SLC25A19	1.088533877	1.30E-09	6.96E-08	down
178	ENSBTAG00000005857	SLC6A1	1.138992596	1.35E-09	7.17E-08	down
179	ENSBTAG00000023523	GKAP1	-1.363619934	1.42E-09	7.53E-08	up
180	ENSBTAG00000010507	SLC22A16	1.156048974	1.59E-09	8.37E-08	down
181	ENSBTAG00000015727	Tgtp1	-1.116923114	1.71E-09	8.90E-08	up
182	ENSBTAG00000001939	SECISBP2	-1.136454401	1.82E-09	9.41E-08	up
183	ENSBTAG00000013222	CD109	-1.257612532	1.84E-09	9.47E-08	up
184	ENSBTAG00000010954	ART3	-1.061108239	2.06E-09	1.05E-07	up
185	ENSBTAG00000044056	C19H17orf98	4.518033825	2.19E-09	1.11E-07	down
186	ENSBTAG00000026275	CCL24	1.45684537	2.30E-09	1.16E-07	down
187	ENSBTAG00000016751	MYO6	-2.334615945	2.36E-09	1.19E-07	up
188	MSTRG.7730	--	-2.247529217	2.51E-09	1.25E-07	up
189	ENSBTAG00000019784	PPP2R5E	-1.178014311	2.55E-09	1.26E-07	up
190	ENSBTAG00000014252	SPTLC3	-1.592331088	3.11E-09	1.52E-07	up
191	ENSBTAG00000012789	PRKD1	-1.27236629	3.48E-09	1.67E-07	up
192	ENSBTAG00000000133	CD68	1.228783644	3.82E-09	1.82E-07	down
193	ENSBTAG00000049122	COL6A5	-2.548092699	4.05E-09	1.92E-07	up
194	ENSBTAG00000002574	MYOZ2	-1.166848538	4.14E-09	1.95E-07	up
195	ENSBTAG00000048696	NCR3LG1	2.967702448	4.20E-09	1.97E-07	down
196	ENSBTAG00000031503	NDUFA4L2	1.04965082	4.26E-09	1.99E-07	down
197	ENSBTAG00000008716	SSB	-1.032169065	4.45E-09	2.07E-07	up
198	ENSBTAG00000008026	OXT	3.287472908	4.66E-09	2.16E-07	down
199	ENSBTAG00000002294	SIM1	8.461070114	4.67E-09	2.16E-07	down
200	MSTRG.5442	--	1.326024203	4.99E-09	2.30E-07	down
201	ENSBTAG00000008040	SPART	-1.516303613	5.19E-09	2.39E-07	up
202	ENSBTAG00000012828	TBC1D15	-1.342785709	5.41E-09	2.48E-07	up
203	ENSBTAG00000011824	OGN	-1.262157147	5.51E-09	2.51E-07	up
204	MSTRG.13986	--	4.051544914	5.84E-09	2.66E-07	down
205	ENSBTAG00000054594	SIRPA	-2.282065886	6.04E-09	2.73E-07	up
206	ENSBTAG00000007489	NCOA3	-1.144940604	6.05E-09	2.73E-07	up
207	ENSBTAG00000002280	KIF5B	-1.463329193	7.03E-09	3.10E-07	up
208	ENSBTAG00000003658	RELN	1.273748922	9.46E-09	4.09E-07	down
209	ENSBTAG00000008626	KCNN1	1.460447959	9.79E-09	4.20E-07	down
210	ENSBTAG00000013204	ATP13A3	-1.593210265	1.03E-08	4.41E-07	up
211	ENSBTAG00000017788	AKT3	-1.657250834	1.10E-08	4.68E-07	up
212	ENSBTAG00000039803	MYO7B	2.671089232	1.15E-08	4.86E-07	down
213	ENSBTAG00000015509	NAMPT	-1.439518031	1.19E-08	4.95E-07	up
214	ENSBTAG00000003120	ZNF385B	-1.478782699	1.23E-08	5.11E-07	up
215	ENSBTAG00000003338	LRRCC1	-1.484659363	1.51E-08	6.23E-07	up
216	MSTRG.8027	--	6.298492218	1.52E-08	6.26E-07	down
217	ENSBTAG00000027684	FOLR2	1.086423272	1.53E-08	6.27E-07	down

218	ENSBTAG00000012446	MTHFSD	1.226640936	1.56E-08	6.36E-07	down
219	ENSBTAG00000009665	UTRN	-1.293584922	1.72E-08	6.94E-07	up
220	ENSBTAG00000001919	ZNF652	-1.253440348	2.01E-08	8.00E-07	up
221	ENSBTAG00000003012	TRAF1	1.066425897	2.32E-08	9.12E-07	down
222	ENSBTAG00000001114	PRKD3	-1.5606997	2.44E-08	9.50E-07	up
223	ENSBTAG00000003062	FHIP2A	-1.285597596	2.45E-08	9.51E-07	up
224	ENSBTAG00000014558	DDX21	-1.179440276	2.59E-08	9.98E-07	up
225	ENSBTAG00000000137	FRYL	-1.313356392	2.71E-08	1.04E-06	up
226	ENSBTAG00000001101	IFNLR1	-1.507835904	2.96E-08	1.12E-06	up
227	ENSBTAG00000004484	CEP83	-1.938912482	3.17E-08	1.19E-06	up
228	ENSBTAG00000007626	IL2RG	1.163560169	3.42E-08	1.29E-06	down
229	ENSBTAG00000010423	LIFR	-1.476865872	3.49E-08	1.31E-06	up
230	ENSBTAG00000053626	TTN	-1.023822956	3.58E-08	1.34E-06	up
231	ENSBTAG00000005681	ME1	-1.044604166	3.72E-08	1.38E-06	up
232	ENSBTAG00000044173	EHBP1	-1.221016752	3.76E-08	1.40E-06	up
233	ENSBTAG00000021565	Prss2	5.389042291	3.91E-08	1.45E-06	down
234	ENSBTAG00000016563	GOLGA4	-1.13839048	3.94E-08	1.46E-06	up
235	ENSBTAG00000025398	UBD	5.120512807	4.07E-08	1.50E-06	down
236	ENSBTAG00000016401	OPTN	-1.259413692	4.18E-08	1.54E-06	up
237	ENSBTAG00000000754	PPP2R5A	-1.09617074	4.24E-08	1.55E-06	down
238	ENSBTAG00000054128	ERVPABLB-1	3.013510286	4.33E-08	1.58E-06	down
239	ENSBTAG00000008483	CALCRL	-1.623970013	4.45E-08	1.62E-06	up
240	ENSBTAG00000016229	KLF9	-1.109532229	4.46E-08	1.62E-06	up
241	ENSBTAG00000003581	SETD7	1.206596929	4.74E-08	1.72E-06	up
242	ENSBTAG00000019733	ADGRL4	1.762964025	4.80E-08	1.73E-06	up
243	ENSBTAG00000016779	CLIP1	-1.135756925	4.96E-08	1.78E-06	up
244	ENSBTAG00000014162	ACKR2	1.370158841	5.21E-08	1.86E-06	down
245	ENSBTAG00000013167	SIGLEC1	2.808499464	5.38E-08	1.92E-06	down
246	ENSBTAG00000007335	TPD52	-1.087553476	5.50E-08	1.95E-06	up
247	ENSBTAG00000020911	FNBP4	-1.316194274	6.24E-08	2.17E-06	up
248	ENSBTAG00000046900	GGT1	1.645357368	6.36E-08	2.20E-06	down
249	MSTRG.7361	--	1.391946542	6.41E-08	2.22E-06	down
250	ENSBTAG00000016494	RELT	1.37089595	7.36E-08	2.53E-06	down
251	ENSBTAG00000008931	CREG1	1.136395203	7.70E-08	2.63E-06	up
252	ENSBTAG00000004079	ZNF106	1.214246796	7.99E-08	2.72E-06	up
253	ENSBTAG00000017561	HHIPL2	-8.082149041	8.09E-08	2.75E-06	up
254	ENSBTAG00000018127	PPM1A	1.135784665	8.12E-08	2.76E-06	up
255	ENSBTAG00000012409	POSTN	2.222851367	8.67E-08	2.93E-06	up
256	ENSBTAG00000022150	MXRA5	1.037400682	9.25E-08	3.11E-06	up
257	ENSBTAG00000013749	RHOQ	1.150571903	9.32E-08	3.13E-06	up
258	ENSBTAG00000013922	MOSPD1	-1.068192507	1.04E-07	3.46E-06	up
259	ENSBTAG00000021059	TNMD	-3.0480186	1.18E-07	3.86E-06	up
260	ENSBTAG00000003550	C29H11orf54	-1.231292057	1.19E-07	3.90E-06	up
261	ENSBTAG00000037996	ARMCX3	-1.105075587	1.22E-07	3.97E-06	up

262	ENSBTAG00000020542	SHE	-1.822057139	1.25E-07	4.04E-06	up
263	ENSBTAG00000005542	EPS15	-1.074184753	1.31E-07	4.22E-06	up
264	ENSBTAG00000033304	TCIM	-1.053588792	1.33E-07	4.29E-06	up
265	ENSBTAG00000002902	ANO6	-1.131952916	1.36E-07	4.36E-06	up
266	ENSBTAG00000003848	ATRN	-1.149672021	1.40E-07	4.49E-06	up
267	ENSBTAG00000020281	NIN	-1.564222306	1.44E-07	4.59E-06	up
268	ENSBTAG00000005773	PGM2	-1.387851673	1.44E-07	4.59E-06	up
269	ENSBTAG00000015200	L2HGDH	-1.37164857	1.48E-07	4.69E-06	up
270	ENSBTAG00000006259	MOB1A	-1.292342534	1.48E-07	4.69E-06	up
271	ENSBTAG00000012191	WWC2	-1.204455529	1.52E-07	4.79E-06	up
272	ENSBTAG00000012966	SCRN3	-1.799730242	1.54E-07	4.83E-06	up
273	ENSBTAG00000009242	ZNF638	-1.14531379	1.65E-07	5.15E-06	up
274	ENSBTAG00000005847	ROCK2	-1.353067446	1.66E-07	5.17E-06	up
275	ENSBTAG00000020505	SBNO1	1.416782957	1.67E-07	5.17E-06	up
276	ENSBTAG00000018690	RPAP3	1.346055664	1.72E-07	5.32E-06	up
277	ENSBTAG00000007321	SREK1	-1.202016572	1.86E-07	5.74E-06	up
278	ENSBTAG00000009441	RBBP6	-1.083121767	2.03E-07	6.20E-06	up
279	ENSBTAG00000012757	GCNT1	1.312781467	2.05E-07	6.25E-06	up
280	ENSBTAG00000017743	XIRP2	1.240360309	2.11E-07	6.39E-06	up
281	ENSBTAG00000013116	PLCB4	-1.180368738	2.15E-07	6.49E-06	up
282	ENSBTAG00000010008	TTC12	1.438423227	2.25E-07	6.77E-06	down
283	ENSBTAG00000008619	SAMD8	-1.394744792	2.43E-07	7.26E-06	up
284	ENSBTAG00000011847	ASPN	-1.410183169	2.55E-07	7.59E-06	up
285	ENSBTAG00000017683	LYSMD2	1.045019613	2.58E-07	7.68E-06	down
286	ENSBTAG00000011748	SGMS1	-1.129348069	2.68E-07	7.94E-06	up
287	ENSBTAG00000027722	IPO7	-1.321511187	2.76E-07	8.14E-06	up
288	ENSBTAG00000006291	CYLD	1.484920748	2.77E-07	8.17E-06	up
289	ENSBTAG00000021020	RIF1	-1.394740085	2.82E-07	8.29E-06	up
290	ENSBTAG00000008921	NEXN	-1.092300996	2.99E-07	8.78E-06	up
291	ENSBTAG00000008285	OXR1	-1.289541066	3.11E-07	9.07E-06	up
292	ENSBTAG00000007506	CACNG7	2.041903953	3.35E-07	9.72E-06	up
293	ENSBTAG00000020735	SMG1	-1.316801617	3.51E-07	1.01E-05	up
294	ENSBTAG00000005376	FBXO30	-1.350635746	3.53E-07	1.02E-05	up
295	ENSBTAG00000011677	H1-2	1.314069128	3.75E-07	1.08E-05	down
296	ENSBTAG00000016005	PPP3CA	-1.090491483	3.84E-07	1.10E-05	up
297	ENSBTAG00000016498	PHACTR2	-1.61108916	4.22E-07	1.20E-05	up
298	ENSBTAG00000032477	HECTD1	-1.158259202	4.71E-07	1.33E-05	up
299	ENSBTAG00000053806	BST2	1.650242194	4.75E-07	1.34E-05	down
300	ENSBTAG00000017809	PDS5A	-1.289913584	4.82E-07	1.35E-05	up
301	ENSBTAG00000004630	COMP	-2.12691498	4.88E-07	1.37E-05	up
302	ENSBTAG00000011096	ERGIC2	-1.279524242	5.08E-07	1.42E-05	up
303	ENSBTAG00000013982	UACA	-1.248233783	5.13E-07	1.43E-05	up
304	ENSBTAG00000027434	SORCS2	1.787796799	5.57E-07	1.53E-05	down
305	ENSBTAG00000050710	ERVPABLB-1	9.048759312	5.59E-07	1.53E-05	down

306	ENSBTAG00000001083	MAP3K20	-1.212849829	5.87E-07	1.60E-05	up
307	ENSBTAG00000009667	SLC25A46	-1.395173528	5.99E-07	1.63E-05	up
308	ENSBTAG00000019284	COPS2	-1.374904492	6.30E-07	1.70E-05	up
309	ENSBTAG00000002640	KALRN	-1.197468919	6.38E-07	1.72E-05	up
310	ENSBTAG00000006471	OSBPL11	-1.322706	6.39E-07	1.72E-05	up
311	ENSBTAG00000000053	FILIP1	-1.107995359	6.41E-07	1.72E-05	up
312	ENSBTAG00000031358	DPYD	-1.112889887	6.68E-07	1.78E-05	up
313	ENSBTAG00000017713	KTN1	-1.20442353	6.81E-07	1.81E-05	up
314	ENSBTAG00000015381	ARHGAP18	-1.172962712	6.89E-07	1.83E-05	up
315	ENSBTAG00000003280	DDA1	1.662246938	7.54E-07	1.98E-05	down
316	ENSBTAG00000002625	MAP3K7	-1.144014862	7.57E-07	1.98E-05	up
317	ENSBTAG00000004924	PENK	-2.153239647	8.63E-07	2.23E-05	up
318	ENSBTAG00000046553	SECISBP2L	-1.335552213	8.78E-07	2.26E-05	up
319	ENSBTAG00000012865	DEK	-1.196925788	8.82E-07	2.27E-05	up
320	ENSBTAG00000000245	NHSL1	1.416878376	9.16E-07	2.35E-05	down
321	ENSBTAG00000012065	DNAJC3	-1.120813291	9.45E-07	2.43E-05	up
322	ENSBTAG00000013615	CHORDC1	1.3237686	9.70E-07	2.47E-05	up
323	ENSBTAG00000019514	OPA1	-1.125018742	9.80E-07	2.49E-05	up
324	ENSBTAG00000005380	MOCS2	1.132332965	1.02E-06	2.58E-05	up
325	ENSBTAG00000023416	PPP2R3A	-1.225940359	1.07E-06	2.71E-05	up
326	ENSBTAG00000008082	CEP350	-1.421410731	1.08E-06	2.71E-05	up
327	ENSBTAG00000017810	EFHC1	1.831367307	1.08E-06	2.71E-05	down
328	ENSBTAG00000013949	AHCTF1	-1.235030686	1.08E-06	2.72E-05	up
329	ENSBTAG00000016525	ITGA1	-1.584833827	1.08E-06	2.72E-05	up
330	ENSBTAG00000008096	EDN1	-1.168242814	1.09E-06	2.72E-05	up
331	MSTRG.9974	--	-1.08642139	1.14E-06	2.83E-05	up
332	ENSBTAG00000038619	Patr-A	-1.340964995	1.20E-06	2.99E-05	up
333	ENSBTAG00000015277	PCF11	-1.141933592	1.21E-06	3.02E-05	up
334	ENSBTAG00000021520	DDX46	-1.340900514	1.24E-06	3.07E-05	up
335	ENSBTAG00000006504	TCHP	-1.156052612	1.26E-06	3.11E-05	up
336	ENSBTAG00000010018	ATP7A	-1.479352313	1.28E-06	3.13E-05	up
337	ENSBTAG00000017141	CGRRF1	-1.422065478	1.29E-06	3.17E-05	up
338	ENSBTAG00000004190	ARHGAP29	-1.22526512	1.31E-06	3.20E-05	up
339	ENSBTAG00000018093	KMT2A	-1.138796131	1.38E-06	3.35E-05	up
340	ENSBTAG00000039117	TOGARAM1	-1.156698153	1.39E-06	3.36E-05	up
341	ENSBTAG00000009055	RNF144B	-1.016472369	1.46E-06	3.52E-05	up
342	ENSBTAG00000020933	HCFC2	-1.37389705	1.47E-06	3.53E-05	up
343	ENSBTAG00000013812	RO60	-1.244447318	1.47E-06	3.53E-05	up
344	ENSBTAG00000000770	PGM2L1	-1.252397806	1.49E-06	3.58E-05	up
345	ENSBTAG00000006320	DBT	-1.28496631	1.53E-06	3.66E-05	up
346	ENSBTAG00000012252	MOCOS	-1.282586344	1.59E-06	3.77E-05	up
347	ENSBTAG00000052708	RC3H1	-1.588598394	1.61E-06	3.81E-05	up
348	ENSBTAG00000049397	CAV2	-1.372614548	1.65E-06	3.88E-05	up
349	ENSBTAG00000011597	PDS5B	-1.502256726	1.73E-06	4.06E-05	up

350	ENSBTAG00000047450	SFR1	-1.435619818	1.76E-06	4.11E-05	up
351	ENSBTAG00000050417	APOL2	-1.490805801	1.80E-06	4.18E-05	up
352	ENSBTAG00000005057	FAM204A	-1.103430556	1.82E-06	4.23E-05	up
353	ENSBTAG00000033735	WASHC4	-1.257206799	1.83E-06	4.23E-05	up
354	MSTRG.1134	--	-1.066536335	1.83E-06	4.23E-05	up
355	ENSBTAG00000008915	SF3B1	-1.107302307	1.84E-06	4.24E-05	up
356	ENSBTAG00000048903	PRNP	-1.255487308	1.91E-06	4.38E-05	up
357	ENSBTAG00000032021	RALB	-1.186296917	1.96E-06	4.49E-05	up
358	ENSBTAG00000011622	C24H18orf21	-1.123712827	2.05E-06	4.64E-05	up
359	ENSBTAG00000006800	FGF6	-2.09207039	2.06E-06	4.66E-05	up
360	MSTRG.14528	--	1.928997909	2.10E-06	4.74E-05	down
361	ENSBTAG00000048960	MZT1	1.382076382	2.17E-06	4.89E-05	up
362	ENSBTAG00000004745	NAA15	1.11776789	2.32E-06	5.17E-05	up
363	ENSBTAG00000009598	CHM	1.315666127	2.33E-06	5.20E-05	up
364	ENSBTAG00000012805	TSPAN13	-1.044239802	2.38E-06	5.30E-05	up
365	ENSBTAG00000027525	NAP1L1	-1.244954104	2.47E-06	5.48E-05	up
366	ENSBTAG00000017719	AKAP6	-1.187371403	2.48E-06	5.50E-05	up
367	ENSBTAG00000021343	ARHGEF12	-1.088055757	2.50E-06	5.52E-05	up
368	ENSBTAG00000000222	ARID4B	-1.13967325	2.51E-06	5.55E-05	up
369	ENSBTAG00000043975	MYCT1	-1.571004043	2.55E-06	5.60E-05	up
370	ENSBTAG00000009012	PTX3	-1.22396812	2.62E-06	5.74E-05	up
371	ENSBTAG00000016512	TTC21B	-1.227809662	2.67E-06	5.83E-05	up
372	ENSBTAG00000006606	RFLNB	-1.692405349	2.67E-06	5.83E-05	up
373	ENSBTAG00000016514	CPE	-1.316975135	2.72E-06	5.93E-05	up
374	ENSBTAG00000011399	YARS2	-1.40171372	2.75E-06	5.97E-05	up
375	ENSBTAG00000012741	CCPG1	-1.289936255	2.75E-06	5.97E-05	up
376	ENSBTAG00000015467	FAM184A	-1.445929639	2.80E-06	6.05E-05	up
377	ENSBTAG00000037937	GVINP1	-2.301086231	2.82E-06	6.10E-05	up
378	ENSBTAG00000000421	EEA1	-1.20995179	3.10E-06	6.56E-05	up
379	ENSBTAG00000018857	RAB4A	-1.282522628	3.25E-06	6.85E-05	up
380	ENSBTAG00000008530	SLC36A4	-1.348296623	3.25E-06	6.85E-05	up
381	ENSBTAG00000015010	TBC1D23	-1.164173656	3.28E-06	6.89E-05	up
382	ENSBTAG00000021762	ZC3H15	-1.019253341	3.34E-06	6.99E-05	up
383	ENSBTAG00000051897	NMNAT1	-1.041197071	3.34E-06	6.99E-05	up
384	ENSBTAG00000003399	SMARCA5	-1.060927527	3.36E-06	7.02E-05	up
385	ENSBTAG00000020193	DCLRE1A	-1.43544215	3.39E-06	7.06E-05	up
386	ENSBTAG00000018460	ADAMTS16	1.569840763	3.49E-06	7.25E-05	down
387	ENSBTAG00000006420	BMPR2	-1.180694523	3.66E-06	7.55E-05	up
388	ENSBTAG00000002293	ARL5A	-1.443968261	3.81E-06	7.81E-05	up
389	ENSBTAG00000006852	ACYP2	-1.131612589	3.83E-06	7.82E-05	up
390	ENSBTAG00000013317	RNF168	-1.389958863	3.85E-06	7.86E-05	up
391	ENSBTAG00000005370	TMTC1	-1.205205842	3.88E-06	7.88E-05	up
392	ENSBTAG00000051082	DST	-1.419554624	3.98E-06	8.06E-05	up
393	ENSBTAG00000006027	USP34	-1.08013807	4.02E-06	8.13E-05	up

394	ENSBTAG00000004672	RDX	-1.14904592	4.12E-06	8.31E-05	up
395	ENSBTAG00000000308	ITCH	-1.225369497	4.19E-06	8.43E-05	up
396	ENSBTAG00000024958	FAM214A	-1.122849048	4.30E-06	8.62E-05	up
397	ENSBTAG00000015222	RSRP1	-1.029529181	4.32E-06	8.65E-05	up
398	ENSBTAG00000048135	IGHG2	1.352200106	4.39E-06	8.79E-05	down
399	ENSBTAG00000009734	XPA	-1.240758779	4.48E-06	8.94E-05	up
400	ENSBTAG00000006645	LPAR6	-1.9878877	4.50E-06	8.96E-05	up
401	ENSBTAG00000010760	REV3L	-1.159462237	4.70E-06	9.32E-05	up
402	ENSBTAG00000011155	PUM2	-1.068859166	4.73E-06	9.35E-05	up
403	ENSBTAG00000018416	CCDC42	2.701401622	4.81E-06	9.50E-05	down
404	ENSBTAG00000006679	MITF	-1.090669832	4.90E-06	9.66E-05	up
405	ENSBTAG00000016750	PTBP3	-1.648625434	5.08E-06	9.97E-05	up
406	ENSBTAG00000010091	ARL6	-1.312048055	5.09E-06	9.99E-05	up
407	ENSBTAG00000001257	AGTPBP1	-1.17228131	5.35E-06	0.000104086	up
408	ENSBTAG00000038233	GBP4	-2.121503045	5.40E-06	0.000104682	up
409	ENSBTAG00000003887	ECHDC1	-1.141104142	5.46E-06	0.000105626	up
410	ENSBTAG00000018986	ACSL4	-1.014433867	5.48E-06	0.000106001	up
411	ENSBTAG00000039520	SIRPA	-5.937215132	5.95E-06	0.000114197	up
412	ENSBTAG00000004850	KPNA3	-1.013902911	6.08E-06	0.00011618	up
413	ENSBTAG00000011401	PDZD8	-1.057231654	6.13E-06	0.00011676	up
414	ENSBTAG00000017455	ADAM9	-1.107930517	6.14E-06	0.00011676	up
415	ENSBTAG00000001282	RALGAPA1	-1.040219231	6.29E-06	0.000119068	up
416	ENSBTAG00000018765	SEMA5B	1.19913895	6.40E-06	0.000120886	down
417	ENSBTAG00000005711	NSG1	-1.362180383	6.43E-06	0.000121281	up
418	ENSBTAG00000007802	BCLAF1	-1.065844514	6.50E-06	0.000122014	up
419	ENSBTAG00000000801	ZNF583	-1.185167926	6.52E-06	0.000122362	up
420	ENSBTAG00000047550	KIAA2026	-1.036326215	6.61E-06	0.000123765	up
421	ENSBTAG00000032656	CPN2	1.802627354	6.80E-06	0.000126639	down
422	ENSBTAG00000016549	CARMIL1	-1.210780532	6.89E-06	0.00012811	up
423	ENSBTAG00000011081	KBTBD8	-1.495095301	6.96E-06	0.000129104	up
424	ENSBTAG00000016495	LINS1	1.192561606	6.97E-06	0.000129188	up
425	ENSBTAG00000018795	PMS1	-1.309863471	7.07E-06	0.000130699	up
426	ENSBTAG00000023867	RC3H2	-1.239032559	7.18E-06	0.000132434	up
427	ENSBTAG00000003288	CCZ1	-1.048787535	7.22E-06	0.000132912	up
428	ENSBTAG00000015022	KIF2A	-1.223218741	7.25E-06	0.000133355	up
429	ENSBTAG00000006121	SBK2	1.602054038	7.35E-06	0.000134961	down
430	ENSBTAG00000031165	TRPM7	1.230225771	7.40E-06	0.000135556	up
431	ENSBTAG00000008390	DNAJC10	-1.085881251	7.54E-06	0.000137638	up
432	ENSBTAG00000001523	YES1	1.422409488	7.60E-06	0.000138399	down
433	ENSBTAG00000020180	TMED5	1.239104971	7.74E-06	0.000140544	up
434	ENSBTAG00000038434	ATRX	-1.267401262	7.76E-06	0.000140896	up
435	ENSBTAG00000013225	NBN	1.183435499	7.86E-06	0.000142361	up
436	ENSBTAG00000010948	CLIC2	1.418020672	7.87E-06	0.000142372	up
437	ENSBTAG00000033801	VRK2	1.225780005	8.11E-06	0.000146238	up

438	ENSBTAG00000032588	TMEM150C	1.187227629	8.47E-06	0.000151568	up
439	ENSBTAG00000010227	CPSF2	1.102589686	8.51E-06	0.000152007	up
440	ENSBTAG00000031709	PDCD10	1.14752218	8.70E-06	0.000154823	up
441	ENSBTAG00000017028	USO1	1.090547222	8.79E-06	0.000155429	up
442	ENSBTAG00000004832	KIF21A	1.273977083	9.11E-06	0.000160401	up
443	ENSBTAG00000048151	PRPF40A	1.170285917	9.21E-06	0.000161612	up
444	ENSBTAG00000012834	ARSI	1.193477572	9.30E-06	0.000162716	down
445	ENSBTAG00000001902	CEP295	1.299651175	9.34E-06	0.000163092	up
446	ENSBTAG00000021694	SPAST	-1.13186715	9.40E-06	0.000163705	up
447	ENSBTAG00000000081	CERT1	-1.071380299	9.41E-06	0.000163705	up
448	ENSBTAG00000021769	CUL3	-1.082258236	9.57E-06	0.000166004	up
449	ENSBTAG00000014958	PRKAR2B	-1.578286436	9.63E-06	0.000166773	up
450	MSTRG.11706	--	-1.701215569	9.75E-06	0.000168683	up
451	ENSBTAG00000020707	ADGRG6	-1.778470177	9.87E-06	0.000170161	up
452	ENSBTAG00000016562	SEC63	-1.092694224	9.87E-06	0.000170161	up
453	ENSBTAG00000051461	SERTM1	-1.489943668	9.89E-06	0.000170296	up
454	ENSBTAG00000046503	NCOA7	-1.379305125	9.91E-06	0.000170425	up
455	ENSBTAG00000048155	FCN1	1.889669756	1.00E-05	0.000171825	down
456	ENSBTAG00000000545	PPP4R3B	-1.08489803	1.00E-05	0.000171825	up
457	ENSBTAG00000013421	PRMT3	-1.244060479	1.02E-05	0.000174194	up
458	ENSBTAG00000021263	CYP4V2	-1.22938053	1.08E-05	0.000182221	up
459	ENSBTAG00000013724	ATG4A	-1.209113404	1.08E-05	0.000182732	up
460	ENSBTAG00000050233	PRRG1	-1.258909558	1.15E-05	0.00019221	up
461	ENSBTAG00000047679	DNTTIP2	-1.315914556	1.16E-05	0.000193658	up
462	ENSBTAG00000016637	WBP4	-1.194934559	1.17E-05	0.000195647	up
463	ENSBTAG00000018527	HDGFL3	-1.438136406	1.19E-05	0.000198726	up
464	ENSBTAG00000004028	POLK	-1.478863928	1.20E-05	0.000199905	up
465	MSTRG.13508	ZNF37A	-1.008114226	1.21E-05	0.000201681	up
466	ENSBTAG00000003481	OTUD6B	-1.158851155	1.27E-05	0.000210376	up
467	ENSBTAG00000008068	ERLEC1	-1.001058616	1.28E-05	0.000210793	up
468	ENSBTAG00000021087	UBR1	-1.206848819	1.30E-05	0.000213637	up
469	ENSBTAG00000051102	KCNK3	1.670397977	1.32E-05	0.000216362	down
470	ENSBTAG00000030855	CHCHD6	1.123842428	1.32E-05	0.000216535	down
471	ENSBTAG00000030174	ACOX1	-1.010249177	1.33E-05	0.000217539	up
472	ENSBTAG00000005596	IGFBP2	1.490181182	1.35E-05	0.000219978	down
473	ENSBTAG00000016557	MTMR2	-1.061470134	1.36E-05	0.000220926	up
474	ENSBTAG00000036282	CCDC59	-1.026373518	1.37E-05	0.000222883	up
475	ENSBTAG00000009230	FBLN7	-1.301959679	1.37E-05	0.000223267	up
476	ENSBTAG00000021602	CTTNBP2NL	-1.07937618	1.39E-05	0.000225934	up
477	ENSBTAG00000004024	UBE2Q2	-1.134308047	1.42E-05	0.000229352	up
478	ENSBTAG00000008755	MARCHF7	-1.140759831	1.42E-05	0.000229464	up
479	ENSBTAG00000037404	LRFN4	1.112202616	1.43E-05	0.000230317	down
480	ENSBTAG00000026953	TRIP11	-1.135764939	1.44E-05	0.000231479	up
481	ENSBTAG00000015291	ORC4	1.342862765	1.46E-05	0.000233891	up

482	ENSBTAG00000013464	CEP97	1.198504444	1.47E-05	0.00023536	up
483	ENSBTAG00000004383	FNBP1L	-1.542871406	1.49E-05	0.000238141	up
484	ENSBTAG00000008167	TTC3	-1.025229193	1.52E-05	0.000241947	up
485	ENSBTAG00000046580	DHX58	1.228429494	1.52E-05	0.000242424	down
486	ENSBTAG00000010006	EMC2	-1.093983727	1.53E-05	0.000243064	up
487	ENSBTAG00000018437	SMC5	-1.314781072	1.57E-05	0.000249279	up
488	ENSBTAG00000002033	UBXN2A	-1.196808523	1.58E-05	0.000249327	up
489	ENSBTAG00000007593	AIDA	-1.260235118	1.60E-05	0.000253076	up
490	ENSBTAG00000014310	HEATR5B	-1.006133048	1.60E-05	0.000253076	up
491	ENSBTAG00000007141	GULP1	-1.909009232	1.61E-05	0.000254081	up
492	ENSBTAG00000014021	UBN2	-1.422416318	1.69E-05	0.000263774	up
493	ENSBTAG00000010124	ATG4C	-1.653640657	1.69E-05	0.000263774	up
494	MSTRG.9971	--	1.644462268	1.70E-05	0.000264514	down
495	ENSBTAG00000012090	GGTA1	-1.099353244	1.71E-05	0.000265815	up
496	ENSBTAG00000039524	APOL2	-1.515583817	1.73E-05	0.000268843	up
497	ENSBTAG00000016462	TCF4	-1.134098787	1.74E-05	0.000269933	up
498	ENSBTAG00000024387	PNPLA8	-1.078478025	1.76E-05	0.000272308	up
499	ENSBTAG00000009121	STAG2	-1.146001793	1.76E-05	0.000272308	up
500	ENSBTAG00000007331	PLOD2	-1.413419221	1.77E-05	0.000272933	up
501	ENSBTAG00000018737	GLMN	-1.394734745	1.77E-05	0.000273155	up
502	ENSBTAG00000021842	FCGR2B	1.753378199	1.79E-05	0.000275291	down
503	ENSBTAG00000016298	TM9SF3	-1.06718554	1.83E-05	0.000280911	up
504	MSTRG.12190	--	-1.529794611	1.87E-05	0.000284648	up
505	ENSBTAG00000004532	FGFR1OP2	-1.515656594	1.87E-05	0.000285251	up
506	ENSBTAG00000021980	RASSF8	-1.010723667	1.89E-05	0.000287116	up
507	ENSBTAG00000013078	DNAH11	1.543142325	1.90E-05	0.000289746	down
508	ENSBTAG00000007272	ATP6V0A2	-1.075234147	1.93E-05	0.000292776	up
509	ENSBTAG00000020713	BACH2	-1.698280044	1.94E-05	0.000293966	up
510	ENSBTAG00000001181	RPS6KC1	-1.000262201	1.96E-05	0.000296391	up
511	ENSBTAG00000010134	OSTM1	-1.425952953	1.97E-05	0.000296391	up
512	ENSBTAG00000005869	SENP6	-1.05495479	1.97E-05	0.000296391	up
513	ENSBTAG00000008942	NGEF	-1.341346808	1.98E-05	0.000297932	up
514	ENSBTAG00000013142	MYNN	-1.247219936	1.99E-05	0.000299918	up
515	ENSBTAG00000018433	DENND6A	-1.375748489	2.01E-05	0.000301764	up
516	ENSBTAG00000022777	CDC42BPA	-1.16020421	2.03E-05	0.000304282	up
517	ENSBTAG00000014002	CEP95	-1.063861978	2.07E-05	0.000308386	up
518	ENSBTAG00000009187	DNAJC28	-1.189682101	2.16E-05	0.00031765	up
519	ENSBTAG00000015334	ZHX1	-1.035327802	2.16E-05	0.00031765	up
520	ENSBTAG00000017233	RNF213	-1.929199719	2.17E-05	0.000318234	up
521	ENSBTAG00000015708	CXCR6	2.323927673	2.19E-05	0.000320036	down
522	MSTRG.3821	--	1.44949697	2.21E-05	0.000323757	down
523	ENSBTAG00000020174	HBS1L	-1.067539909	2.22E-05	0.000324201	up
524	ENSBTAG00000014029	HERC4	1.176168282	2.30E-05	0.000332616	up
525	ENSBTAG00000005865	MAPK6	-1.032594831	2.31E-05	0.000333826	up

526	ENSBTAG00000019568	IFT74	-1.34075374	2.31E-05	0.000333826	up
527	ENSBTAG00000001810	SCAF11	-1.110858806	2.32E-05	0.000335419	up
528	ENSBTAG00000018613	NOL8	-1.313631369	2.37E-05	0.000340093	up
529	ENSBTAG00000008366	STAC2	3.407722116	2.41E-05	0.000344491	down
530	ENSBTAG00000012545	GOLM2	-1.05764033	2.44E-05	0.000348836	up
531	ENSBTAG00000020031	PKD2	-1.059525897	2.46E-05	0.000350974	up
532	MSTRG.7255	--	1.38332864	2.47E-05	0.000351185	down
533	ENSBTAG00000003040	RALGAPB	-1.000382668	2.54E-05	0.000360203	up
534	ENSBTAG00000019409	GNPTAB	-1.026019421	2.55E-05	0.000361468	up
535	ENSBTAG00000049233	TMEM123	-1.024109966	2.56E-05	0.000361762	up
536	ENSBTAG00000005012	HSPH1	-1.013606256	2.58E-05	0.000362959	up
537	ENSBTAG00000007678	MKX	-3.489286023	2.66E-05	0.000372055	up
538	ENSBTAG00000001603	YIPF7	-1.008730299	2.68E-05	0.000373833	up
539	ENSBTAG00000019843	HAUS6	-1.112663108	2.68E-05	0.000373833	up
540	ENSBTAG00000010546	LIN54	-1.105978253	2.68E-05	0.000374103	up
541	ENSBTAG00000030744	NFYB	-1.131822869	2.72E-05	0.000377649	up
542	ENSBTAG00000001945	ARG2	1.111087529	2.72E-05	0.000377649	down
543	ENSBTAG00000013678	OSGIN2	-1.262963234	2.72E-05	0.000377722	up
544	ENSBTAG00000014099	YTHDC2	-1.199110758	2.76E-05	0.000380699	up
545	ENSBTAG00000006369	MRPS35	-1.146687493	2.80E-05	0.000385837	up
546	ENSBTAG00000008388	THOC2	-1.131100549	2.83E-05	0.000389153	up
547	ENSBTAG00000053445	TMEM225B	-1.820475072	2.87E-05	0.000393462	up
548	ENSBTAG00000002725	RNGTT	-1.094836004	2.87E-05	0.000393462	up
549	ENSBTAG00000035907	DDX3X	-1.035004643	2.88E-05	0.000393699	up
550	ENSBTAG00000012882	CUL5	-1.286296943	2.89E-05	0.000395256	up
551	ENSBTAG00000012128	AASS	-1.073277805	2.92E-05	0.000398431	up
552	ENSBTAG00000039770	CEBPZ	-1.144498783	2.98E-05	0.000404878	up
553	ENSBTAG00000004842	RSPRY1	-1.141607922	3.00E-05	0.000406249	up
554	ENSBTAG00000051486	UFM1	-1.00657747	3.00E-05	0.000406249	up
555	ENSBTAG00000033446	TATDN1	-1.113161506	3.01E-05	0.000407245	up
556	ENSBTAG00000006040	UHRF1BP1L	-1.414551334	3.09E-05	0.000415617	up
557	ENSBTAG00000033077	NUFIP2	-1.077852935	3.09E-05	0.000415617	up
558	ENSBTAG00000020410	UFL1	-1.019095993	3.18E-05	0.000425754	up
559	ENSBTAG00000000201	LTN1	-1.20301857	3.20E-05	0.000427881	up
560	ENSBTAG00000000920	DNAH17	-1.934112064	3.32E-05	0.000442394	up
561	ENSBTAG00000001509	ELK3	-1.151460421	3.39E-05	0.000449412	up
562	ENSBTAG00000003438	ZBTB43	-1.117359255	3.41E-05	0.000451935	up
563	ENSBTAG00000051412	HBA	1.809597359	3.44E-05	0.000454907	down
564	ENSBTAG00000009232	PIK3CA	-1.039332412	3.49E-05	0.000461106	up
565	ENSBTAG00000004934	NEMF	-1.151679054	3.54E-05	0.000466033	up
566	ENSBTAG00000020296	UBR3	-1.018377868	3.56E-05	0.000467649	up
567	ENSBTAG00000046239	LCOR	-1.062393604	3.57E-05	0.000467716	up
568	ENSBTAG00000019472	NR3C1	-1.010898247	3.58E-05	0.000468207	up
569	ENSBTAG00000005308	ANGPTL5	-2.521907426	3.58E-05	0.00046843	up

570	ENSBTAG00000019853	SMURF2	-1.167941697	3.64E-05	0.000475694	up
571	ENSBTAG00000040001	MARVELD2	-1.731587414	3.73E-05	0.00048471	up
572	ENSBTAG00000039287	ZFP69	-1.213250973	3.78E-05	0.000491293	up
573	ENSBTAG00000013938	PHC3	-1.32722776	3.82E-05	0.000495278	up
574	ENSBTAG00000019750	TMEM106B	-1.082276831	3.83E-05	0.000496419	up
575	ENSBTAG00000012454	SLC35A3	-1.470934194	4.05E-05	0.000520491	up
576	ENSBTAG00000022169	PREX2	-1.363763151	4.10E-05	0.000525391	up
577	ENSBTAG00000001805	TWSG1	1.213536902	4.16E-05	0.000532199	up
578	ENSBTAG00000013676	DYNC2LI1	1.572945365	4.20E-05	0.000535779	up
579	ENSBTAG00000030424	CLEC1A	-1.082634202	4.22E-05	0.00053774	up
580	ENSBTAG00000006708	ARIH1	-1.012956672	4.25E-05	0.000539657	up
581	ENSBTAG00000039671	DNAJC10	-3.587766574	4.25E-05	0.000539862	up
582	ENSBTAG00000006697	RICTOR	-1.200895721	4.33E-05	0.000546094	up
583	ENSBTAG00000010485	MFN1	-1.02865299	4.33E-05	0.000546094	up
584	ENSBTAG00000004901	PIK3C2A	-1.586957378	4.34E-05	0.000546094	up
585	ENSBTAG00000011895	PANK3	-1.248056132	4.35E-05	0.000547268	up
586	ENSBTAG00000017442	CDO1	-1.186914526	4.44E-05	0.000558092	up
587	ENSBTAG00000015428	TMEM170A	-1.294705973	4.51E-05	0.000564473	up
588	ENSBTAG00000020936	NOD2	-1.468322644	4.53E-05	0.000566382	up
589	ENSBTAG00000014602	CAMTA1	-1.534697257	4.57E-05	0.00057106	up
590	ENSBTAG00000020569	CACNA2D1	-1.069941249	4.66E-05	0.000579218	up
591	ENSBTAG00000003960	CCP110	-1.323336633	4.71E-05	0.000584491	up
592	ENSBTAG00000000295	CLHC1	-1.284561242	4.75E-05	0.000587562	up
593	ENSBTAG00000020164	BAZ1A	-1.22289016	4.76E-05	0.000588444	up
594	ENSBTAG00000011733	GIPC2	-1.814603499	4.79E-05	0.000590492	up
595	ENSBTAG00000016804	LYST	-1.463484623	4.95E-05	0.000607092	up
596	ENSBTAG00000031012	PIGK	-1.381379121	4.97E-05	0.00060877	up
597	ENSBTAG00000047441	HSPD1	-1.127932811	5.01E-05	0.000612797	up
598	ENSBTAG00000025358	SNX2	-1.036751613	5.05E-05	0.000616853	up
599	ENSBTAG00000017242	FADS6	1.20948266	5.11E-05	0.000622977	down
600	ENSBTAG00000012630	PAMR1	1.289378281	5.15E-05	0.000627082	down
601	ENSBTAG00000052214	ERVPABLB-1	6.132958675	5.19E-05	0.000631606	down
602	ENSBTAG00000018994	TNFSF10	-1.400091076	5.20E-05	0.000631921	up
603	ENSBTAG00000019036	ARHGAP28	-1.464178411	5.26E-05	0.000639052	up
604	ENSBTAG00000031561	RSRC1	-1.083269308	5.28E-05	0.000640425	up
605	ENSBTAG00000002020	CREBRF	-1.468166037	5.31E-05	0.000642717	up
606	ENSBTAG00000001425	FBXO11	-1.007672645	5.33E-05	0.000644548	up
607	ENSBTAG00000005165	CSGALNACT2	-1.067196836	5.41E-05	0.000652247	up
608	ENSBTAG00000006511	MTF1	-1.193294356	5.60E-05	0.000670853	up
609	ENSBTAG00000046768	IGFBP1	-1.494311496	5.76E-05	0.000686321	up
610	ENSBTAG00000006138	SEMA3C	-1.084991324	5.77E-05	0.000686788	up
611	ENSBTAG00000020748	FAM76B	-1.635625424	5.97E-05	0.000705744	up
612	ENSBTAG00000025029	MAN2A1	-1.082140496	6.01E-05	0.000709617	up
613	ENSBTAG00000037644	HBB	4.382765681	6.01E-05	0.000709617	down

614	ENSBTAG00000035230	TOR1AIP2	-1.246680923	6.07E-05	0.000713727	up
615	ENSBTAG00000006748	DMXL1	-1.262348778	6.09E-05	0.000715383	up
616	ENSBTAG00000009698	ABCD3	-1.037753472	6.10E-05	0.000715383	up
617	ENSBTAG00000000579	NIPBL	-1.040191072	6.11E-05	0.000716596	up
618	ENSBTAG00000017639	RPS6KA3	-1.086788185	6.12E-05	0.000716886	up
619	ENSBTAG00000007611	NRIP2	1.083639104	6.13E-05	0.000717865	down
620	ENSBTAG00000047902	ULBP1	-1.348291554	6.18E-05	0.000721103	up
621	ENSBTAG00000014752	AKAP11	-1.222392421	6.19E-05	0.000721883	up
622	ENSBTAG00000007236	PCM1	-1.057411187	6.21E-05	0.000723531	up
623	ENSBTAG00000019043	GABPA	-1.013922964	6.24E-05	0.00072617	up
624	ENSBTAG00000010152	MID1	-1.315224037	6.32E-05	0.00073364	up
625	ENSBTAG00000047874	PRPF38B	-1.043866301	6.34E-05	0.000736009	up
626	ENSBTAG00000051141	pol	-4.187778666	6.41E-05	0.000741475	up
627	ENSBTAG00000011761	LRP6	-1.213362268	6.44E-05	0.000744044	up
628	ENSBTAG00000009087	GNG10	-1.029603499	6.46E-05	0.000746425	up
629	ENSBTAG00000002795	NKTR	-1.08731156	6.50E-05	0.000748878	up
630	ENSBTAG00000011313	CASP8AP2	-1.578280251	6.50E-05	0.000748878	up
631	ENSBTAG00000024426	PPP1R9A	-1.116183901	6.67E-05	0.000763026	up
632	ENSBTAG00000012550	TAOK3	-1.113802391	6.70E-05	0.000765476	up
633	MSTRG.8188	--	3.372167723	6.72E-05	0.000766594	down
634	ENSBTAG00000013476	CPA5	1.088481557	6.72E-05	0.000766594	down
635	ENSBTAG00000007382	SCAPER	-1.446642647	6.74E-05	0.000767513	up
636	ENSBTAG00000002979	PIK3R3	-1.528195761	6.78E-05	0.000770926	up
637	ENSBTAG00000014792	MAP4K5	-1.041767007	6.80E-05	0.000771841	up
638	ENSBTAG00000008333	ETV4	-1.169217625	6.80E-05	0.000771841	up
639	ENSBTAG00000011116	PAQR9	1.70856987	7.02E-05	0.000792109	down
640	ENSBTAG00000009836	CHGA	7.836681593	7.07E-05	0.000795441	down
641	ENSBTAG00000015371	GABPB2	-1.510336835	7.17E-05	0.000805306	up
642	ENSBTAG00000011509	SERAC1	-1.173720918	7.36E-05	0.000819783	up
643	ENSBTAG00000005305	NTS	4.203801994	7.37E-05	0.000819783	down
644	ENSBTAG00000003690	NHLRC2	-1.060976321	7.44E-05	0.000827421	up
645	ENSBTAG00000009091	RNASEL	-1.393172353	7.50E-05	0.000832972	up
646	ENSBTAG00000023279	N4BP2L2	-1.177067758	7.91E-05	0.000872133	up
647	ENSBTAG00000007693	SLC45A3	2.044812013	7.94E-05	0.000873414	down
648	ENSBTAG00000015868	LIG4	-1.181944632	7.95E-05	0.000874691	up
649	ENSBTAG00000009575	TBC1D32	-3.089920585	8.09E-05	0.000886843	up
650	ENSBTAG00000013621	DPY19L4	-1.311348344	8.25E-05	0.000901147	up
651	ENSBTAG00000010850	SERTAD4	-2.469428519	8.42E-05	0.000917034	up
652	ENSBTAG00000017429	PREPL	-1.005198907	8.57E-05	0.000931496	up
653	ENSBTAG00000015474	SRSF11	-1.238170471	8.64E-05	0.00093742	up
654	ENSBTAG00000006836	FBXO33	-1.612197493	8.68E-05	0.000941267	up
655	ENSBTAG00000000363	BDP1	-1.177658946	8.70E-05	0.000942995	up
656	ENSBTAG00000012423	SENP1	-1.563423921	8.74E-05	0.000946357	up
657	ENSBTAG00000006878	Tafl	-1.103874834	8.77E-05	0.000948269	up

658	ENSBTAG00000013961	MYSM1	-1.348602847	8.78E-05	0.000948269	up
659	ENSBTAG00000003098	MTDH	-1.021664028	8.83E-05	0.000952225	up
660	ENSBTAG00000005481	ADAM10	-1.148696176	8.91E-05	0.000957433	up
661	ENSBTAG00000032674	SENP5	-1.44366901	8.94E-05	0.000959455	up
662	ENSBTAG00000007608	BMT2	-1.336192889	9.13E-05	0.000977584	up
663	ENSBTAG00000047147	CRYBG3	-1.134869944	9.27E-05	0.000992033	up
664	ENSBTAG00000020471	SAMD15	-1.91075954	9.48E-05	0.001011836	up
665	ENSBTAG00000021910	TBL1XR1	-1.126541727	9.58E-05	0.00102219	up
666	ENSBTAG00000013616	AP4E1	-1.063831164	9.60E-05	0.001023009	up
667	MSTRG.3017		5.086743472	9.80E-05	0.001040197	down
668	ENSBTAG00000003546	TFAM	-1.236494727	9.81E-05	0.001040197	up
669	ENSBTAG00000026501	CYP2D14	1.318532185	9.87E-05	0.001044516	down
670	ENSBTAG00000033662	NCKAP1	-1.113737015	9.93E-05	0.001048669	up
671	ENSBTAG00000003354	SMCHD1	-1.052566965	9.93E-05	0.001048669	up
672	ENSBTAG00000031010	ACAD11	-1.072070554	0.000100268	0.001055983	up
673	ENSBTAG00000018674	PAK1IP1	-1.000134038	0.000100877	0.001061734	up
674	ENSBTAG00000031609	THAP12	-1.082557111	0.000101419	0.001064114	up
675	ENSBTAG00000015202	DMAC2L	-1.050859333	0.000102095	0.001069866	up
676	MSTRG.6670	--	-1.106353716	0.000102694	0.001074146	up
677	ENSBTAG00000006751	TENT2	-1.033811711	0.000103693	0.001081496	up
678	ENSBTAG00000023172	ITSN2	-1.450185199	0.000103718	0.001081496	up
679	ENSBTAG00000016368	LRPPRC	-1.001234087	0.000104252	0.001085716	up
680	ENSBTAG00000011626	ATP2C1	-1.023704497	0.000106243	0.001101686	up
681	ENSBTAG00000006428	ZNF354A	-1.534968065	0.000108024	0.001118768	up
682	ENSBTAG00000006869	PHF20L1	-1.369060677	0.000110279	0.001137231	up
683	ENSBTAG00000052979	TMEM69	-1.325201322	0.000111003	0.001142608	up
684	ENSBTAG00000008219	RBM7	1.039849306	0.000111072	0.001142608	up
685	ENSBTAG00000026768	TH	1.376894554	0.000111266	0.001143907	down
686	ENSBTAG00000001323	CENPC	1.224398752	0.000111667	0.001146631	up
687	ENSBTAG00000012908	HSPA13	1.447949356	0.000112876	0.001156222	up
688	ENSBTAG00000024787	HAUS3	1.266714752	0.000113685	0.001161685	up
689	ENSBTAG00000031682	ZNF484	1.584571791	0.000114349	0.001167759	up
690	ENSBTAG00000007730	ZFX	-1.007289967	0.000116072	0.001182451	up
691	ENSBTAG00000000285	GUF1	1.185504791	0.00011623	0.00118267	up
692	ENSBTAG00000017565	SCFD1	1.120341633	0.00011648	0.001184499	up
693	ENSBTAG00000017597	ESCO1	1.110165029	0.000116822	0.00118726	up
694	ENSBTAG00000010362	NOSTRIN	1.164654471	0.000117329	0.001190971	up
695	ENSBTAG00000044074	MLLT10	-1.029482205	0.000117708	0.001193948	up
696	ENSBTAG00000009061	FAR1	-1.083689649	0.000117764	0.001193948	up
697	ENSBTAG00000000869	OBI1	1.233039666	0.000117883	0.001194432	up
698	ENSBTAG00000004922	AGPAT5	-1.029720097	0.000118633	0.001199168	up
699	ENSBTAG00000004709	MAPK9	-1.204914933	0.000122086	0.001228153	up
700	ENSBTAG00000007709	SHOC2	-1.03058832	0.000123593	0.001240349	up
701	MSTRG.6327	GRPEL1	3.231065296	0.000123977	0.001241504	down

702	ENSBTAG00000021226	SAR1B	-1.054025848	0.000124702	0.001246282	up
703	ENSBTAG00000005443	MIER1	-1.344821381	0.000127286	0.001268337	up
704	ENSBTAG00000012260	MTRF1	-1.094125588	0.000128709	0.001278736	up
705	ENSBTAG00000003545	TAF1D	-1.321875335	0.000130646	0.001295691	up
706	ENSBTAG00000040206	ZNF770	-1.159569671	0.000132636	0.001310019	up
707	ENSBTAG00000014011	TMOD2	1.243513292	0.000133891	0.001320092	up
708	ENSBTAG00000007634	HOOK3	1.255616241	0.000136029	0.001332597	up
709	ENSBTAG00000003097	DENND4C	-1.290877699	0.000137139	0.001341125	up
710	ENSBTAG00000031701	TAGAP	1.135710381	0.000137409	0.001342983	down
711	ENSBTAG00000002073	RAD17	-1.005775408	0.000140541	0.001367246	up
712	ENSBTAG00000009778	KRAS	-1.317337847	0.000140966	0.001369008	up
713	ENSBTAG00000055046	--	1.952017424	0.000141897	0.001374091	down
714	ENSBTAG00000017573	RBM26	1.033372229	0.00014407	0.001390338	up
715	MSTRG.6429	--	1.228370098	0.000146098	0.001404669	up
716	ENSBTAG00000005769	NPHP3	1.875692052	0.000146139	0.001404669	up
717	ENSBTAG00000010372	MTMR7	1.008271408	0.000146239	0.001404834	up
718	ENSBTAG00000009482	AFF4	-1.031702758	0.000148795	0.001425857	up
719	ENSBTAG00000007460	RAB11FIP2	1.115612879	0.000148938	0.001425873	up
720	ENSBTAG00000005668	SLC39A8	1.292752569	0.000149294	0.001428098	up
721	ENSBTAG00000014616	SEPSECS	1.824632913	0.000150455	0.001435084	up
722	ENSBTAG00000033078	CNEP1R1	-1.028619531	0.000151007	0.001439134	up
723	ENSBTAG00000011176	TPRKB	-1.065120563	0.000151263	0.001440765	up
724	ENSBTAG00000010343	C1QTNF7	-1.072797624	0.00015226	0.001447802	up
725	ENSBTAG00000003786	GCGR	-1.166846923	0.000153172	0.001451555	up
726	ENSBTAG00000011613	PLS3	-1.030124018	0.00015381	0.001455968	up
727	ENSBTAG00000010609	STK31	-2.069348047	0.000155509	0.001469813	up
728	ENSBTAG00000008016	STX17	-1.002581553	0.000155575	0.001469813	up
729	ENSBTAG00000013196	STK17B	-1.848104055	0.000155622	0.001469813	up
730	ENSBTAG00000010313	DDX52	-1.031799589	0.000156302	0.001475132	up
731	ENSBTAG00000000125	SPATA5	-1.283627855	0.00015653	0.001475905	up
732	ENSBTAG00000018347	IL33	-1.318315167	0.000158573	0.00149183	up
733	ENSBTAG00000051480	Fgf13	-1.011465186	0.000159498	0.001496348	up
734	ENSBTAG00000052983	ARMCX4	-1.471964567	0.000161843	0.001515811	up
735	ENSBTAG00000021318	SCAMP5	1.077746582	0.000162049	0.001516903	down
736	ENSBTAG00000014655	MYO1A	-1.893902071	0.000162736	0.001521638	up
737	ENSBTAG00000015554	TMF1	-1.044256894	0.000166245	0.001548916	up
738	ENSBTAG00000018743	C5H12orf29	-1.023363193	0.000166297	0.001548916	up
739	ENSBTAG00000011147	RNF13	-1.063631712	0.000166983	0.00155445	up
740	ENSBTAG00000020495	SH3YL1	-1.144883998	0.000167913	0.001562243	up
741	ENSBTAG00000003586	MAN1A2	-1.24724853	0.000168363	0.001565566	up
742	ENSBTAG00000007442	AKAP9	-1.013913666	0.000168694	0.001566912	up
743	ENSBTAG00000019889	DIS3	-1.022759903	0.000170636	0.001582327	up
744	ENSBTAG00000039442	SEC62	-1.023402379	0.000176769	0.00162933	up
745	ENSBTAG00000022986	TAFA5	1.528933818	0.000178	0.001637985	down

746	ENSBTAG00000010522	CD163	3.504994196	0.000180255	0.001655119	down
747	ENSBTAG00000014007	ELMOD2	-1.206921013	0.000182174	0.001669099	up
748	ENSBTAG00000009637	SLC12A2	-1.015289496	0.000185736	0.001696205	up
749	ENSBTAG00000014091	ARHGEF3	-1.087462841	0.000186901	0.001704068	up
750	ENSBTAG00000011758	DNAJC19	10.24721548	0.000189497	0.001719207	down
751	ENSBTAG00000014123	ZNF507	-1.01579871	0.000190077	0.001722762	up
752	ENSBTAG00000012797	DCUN1D1	1.089531688	0.000190522	0.001725746	up
753	ENSBTAG00000018408	SNX14	-1.017364643	0.000190654	0.001725746	up
754	ENSBTAG00000018694	ACSS3	-1.162634203	0.000194849	0.001753743	up
755	ENSBTAG00000054911	ANO9	-1.104301478	0.000195667	0.001756406	up
756	ENSBTAG00000002726	CDC27	-1.075750321	0.000197066	0.001763331	up
757	ENSBTAG00000047213	GPRASP1	-1.86425132	0.000199094	0.001779517	up
758	ENSBTAG00000006837	UBA6	-1.098078968	0.000202457	0.001799953	up
759	MSTRG.4179	--	1.035166705	0.000204626	0.001815541	down
760	ENSBTAG00000049036	ERVPABLB-1	3.853194999	0.00020726	0.001832156	down
761	ENSBTAG00000051072	AQP4	-1.235280905	0.000207497	0.001833287	up
762	ENSBTAG00000019020	TAX1BP1	-1.050890093	0.000209749	0.001850272	up
763	ENSBTAG00000004394	PKIB	-1.737670498	0.000215452	0.001895672	up
764	ENSBTAG00000011946	ODR4	-1.024439856	0.000218657	0.001917807	up
765	ENSBTAG00000017179	USP12	-1.024617187	0.000219734	0.001924239	up
766	ENSBTAG00000021543	MDFIC	-1.004062298	0.000221884	0.001939034	up
767	ENSBTAG00000007817	BRCC3	-1.263722006	0.000224131	0.001955626	up
768	ENSBTAG00000006743	RBBP8	-1.058347627	0.000226425	0.001970537	up
769	ENSBTAG00000006666	NAA25	-1.100914144	0.000227455	0.00197365	up
770	ENSBTAG00000006439	DOCK11	-1.185118393	0.000228364	0.001980254	up
771	ENSBTAG00000007988	STX2	-1.079644234	0.00023103	0.001996178	up
772	ENSBTAG00000048861	FAM151A	8.020146573	0.000232001	0.002000952	down
773	ENSBTAG00000017287	DRAM1	-1.001190974	0.000233666	0.002013789	up
774	ENSBTAG00000015654	PON1	-1.417125638	0.000236221	0.002031647	up
775	ENSBTAG00000053649	CCL5	1.820016393	0.00023663	0.002033087	down
776	ENSBTAG00000005257	PEX13	-1.191012859	0.000238847	0.002048998	up
777	ENSBTAG00000013201	ALOX5AP	1.41920561	0.000239787	0.002053802	down
778	ENSBTAG00000001687	STC1	-1.281096339	0.000241328	0.002061878	up
779	ENSBTAG00000053324	Pol	6.244125943	0.000242199	0.002067219	down
780	ENSBTAG00000021900	PKN2	-1.283351633	0.000242351	0.002067476	up
781	ENSBTAG00000011098	TTLL8	-3.928916902	0.000245512	0.002089031	up
782	ENSBTAG00000006555	ZMYM1	-1.103117822	0.000246666	0.002094737	up
783	ENSBTAG00000019026	EXTL2	-1.070564694	0.000254923	0.002152913	up
784	ENSBTAG00000003440	FRRS1	-1.515810524	0.000256958	0.002164458	up
785	ENSBTAG00000032637	EXOC1	-1.021232637	0.000257513	0.002165141	up
786	ENSBTAG00000008204	CARNMT1	-1.29793559	0.000257657	0.002165141	up
787	ENSBTAG00000005679	TMEM130	4.403999228	0.0002578	0.002165262	down
788	ENSBTAG00000035437	STAG1	-1.006888163	0.000259642	0.002179651	up
789	ENSBTAG00000053471	pol	-2.144693262	0.000260852	0.002184361	up

790	ENSBTAG00000018330	RAD9B	-1.14358438	0.000266374	0.002221762	up
791	ENSBTAG00000015904	RORA	-1.039070027	0.000273661	0.002270688	up
792	ENSBTAG00000014933	TRAK2	-1.420723576	0.00027605	0.002286608	up
793	ENSBTAG00000021023	ZMYM4	-1.191467535	0.000276782	0.00229079	up
794	ENSBTAG00000000328	TPPP2	-2.364479942	0.000276826	0.00229079	up
795	ENSBTAG00000009680	FAM177A1	-1.001865901	0.000281912	0.002322731	up
796	ENSBTAG00000013036	YDJC	1.425869267	0.000282978	0.002327971	down
797	ENSBTAG00000001894	NEDD1	-1.030521551	0.000286089	0.002346685	up
798	ENSBTAG00000008809	PTBP2	-1.397542831	0.000286743	0.002348613	up
799	ENSBTAG00000031913	UQCRC2	2.938599455	0.000288211	0.00235949	down
800	ENSBTAG00000009308	TDRD3	-1.103742265	0.000288897	0.002363959	up
801	ENSBTAG00000053537	DOCK11	-1.438421237	0.000291213	0.002379191	up
802	ENSBTAG00000044097	KLF7	-1.241443611	0.000292039	0.002380927	up
803	ENSBTAG00000044202	CNKSR2	-1.102223509	0.000296411	0.002409055	up
804	ENSBTAG00000004660	SPATS2	1.153567632	0.000297785	0.002417887	up
805	ENSBTAG00000003111	ATM	-1.227704106	0.000298727	0.002424367	up
806	ENSBTAG00000012448	CCDC91	-1.043656396	0.00030093	0.002435512	up
807	ENSBTAG00000052883	NANOS1	-1.42824184	0.000306457	0.00246577	up
808	ENSBTAG00000044044	CLOCK	-1.443689687	0.000310611	0.002491991	up
809	ENSBTAG00000001080	SPAG17	-2.422532036	0.000313706	0.002512934	up
810	MSTRG.551	--	1.153970809	0.000313822	0.002512934	down
811	ENSBTAG00000013042	EXOC5	-1.062228136	0.000314245	0.002513957	up
812	ENSBTAG00000008457	SLU7	-1.11164617	0.000322613	0.002571136	up
813	ENSBTAG00000002504	IREB2	-1.048004608	0.00033342	0.002644745	up
814	ENSBTAG00000006118	RSRC2	-1.024934407	0.000337933	0.002671736	up
815	ENSBTAG00000025477	KIF3A	-1.194774759	0.000338881	0.002676714	up
816	ENSBTAG00000016104	BTAF1	-1.248696709	0.000353265	0.002766965	up
817	ENSBTAG00000009137	NKG7	1.244485997	0.000363741	0.002838462	down
818	ENSBTAG00000018767	RTP3	-1.306543224	0.000370163	0.002872246	up
819	MSTRG.13213	--	-2.143724529	0.000370288	0.002872246	up
820	ENSBTAG00000007939	CHMP2B	-1.031504375	0.000378325	0.002923816	up
821	ENSBTAG00000044065	PITPNC1	-1.787059987	0.000380096	0.002932704	up
822	ENSBTAG00000000290	ATP11B	-1.232985916	0.000381387	0.002936698	up
823	ENSBTAG00000020890	TRIM66	1.718726476	0.000390068	0.002986073	down
824	ENSBTAG00000012929	CEP85L	-1.180182907	0.000392423	0.003001093	up
825	ENSBTAG00000000561	OCLN	-1.032823295	0.000401402	0.003051382	up
826	ENSBTAG00000014340	KERA	-1.476459377	0.000402343	0.003053381	up
827	ENSBTAG00000019704	HLTF	-1.06221439	0.000404999	0.003070368	up
828	ENSBTAG00000008471	MX2	2.097690341	0.000407558	0.003084622	down
829	ENSBTAG00000021671	METTL18	-1.250468559	0.000407815	0.003085177	up
830	ENSBTAG00000021761	BRAF	-1.009781021	0.000412035	0.003111514	up
831	ENSBTAG00000055062	SMAD5	-1.000093971	0.000414042	0.003122468	up
832	ENSBTAG00000013412	NFAT5	-1.231739108	0.000419291	0.003156396	up
833	ENSBTAG00000021726	CWC22	-1.009524429	0.000419987	0.003157405	up

834	ENSBTAG00000014169	PCDH19	-2.616821415	0.000422053	0.003171518	up
835	ENSBTAG00000012694	UHMK1	-1.205202003	0.000429684	0.003211664	up
836	ENSBTAG00000049426	PCTP	-1.02435976	0.000431284	0.00322163	up
837	ENSBTAG00000049147	pol	-1.286459984	0.000432192	0.00322421	up
838	ENSBTAG00000039196	C4BPA	1.266780514	0.00043232	0.00322421	down
839	ENSBTAG00000003752	SLC25A24	-1.116199328	0.000433854	0.003231355	up
840	ENSBTAG00000050482	ZNF148	-1.116166348	0.000436706	0.003246859	up
841	ENSBTAG00000002177	PIKFYVE	-1.369080714	0.000439186	0.003262416	up
842	ENSBTAG00000014399	QSER1	-1.027299733	0.000439473	0.003263105	up
843	ENSBTAG00000003892	CMAH	-2.214691529	0.000443111	0.003284328	up
844	ENSBTAG00000007330	STXBP3	-1.045454651	0.000444353	0.003292081	up
845	ENSBTAG00000047673	THUMPD1	-1.177858828	0.000475026	0.003474993	up
846	ENSBTAG00000021921	STRBP	-1.220704861	0.000480217	0.003501828	up
847	ENSBTAG00000017711	WHAMM	-1.687073508	0.000480586	0.003501975	up
848	ENSBTAG00000005218	PDE3B	-2.46208412	0.000485175	0.003529311	up
849	ENSBTAG00000010286	NPAT	-1.088437965	0.000486693	0.003538822	up
850	ENSBTAG00000000581	DOCK10	-1.016636002	0.000499843	0.003621925	up
851	ENSBTAG00000007130	ESF1	-1.130189868	0.000509315	0.003674749	up
852	ENSBTAG00000021975	PATJ	-1.086652006	0.000531829	0.003804588	up
853	ENSBTAG00000017124	GPSM2	-1.235201647	0.000534192	0.003818246	up
854	ENSBTAG00000002612	FANCM	-1.177327834	0.000541898	0.003866764	up
855	ENSBTAG00000004718	PUS7L	-1.340787122	0.000547987	0.003897002	up
856	ENSBTAG00000020696	HECW2	-1.318043856	0.000554652	0.003932773	up
857	ENSBTAG00000040147	SYNDIG1	1.623152065	0.000555436	0.003936679	down
858	ENSBTAG00000020436	KIF27	-1.339001608	0.000556489	0.003942483	up
859	ENSBTAG00000007288	CDK19	-1.294323248	0.000562152	0.003975912	up
860	ENSBTAG00000008293	GPCPD1	-1.108824426	0.000573689	0.00403885	up
861	ENSBTAG00000012391	MINDY2	-1.206361702	0.000576606	0.004057686	up
862	ENSBTAG00000012186	DKKL1	1.697845578	0.000578188	0.004065423	down
863	ENSBTAG00000009570	ANGPTL8	1.364424985	0.000585479	0.004111141	down
864	ENSBTAG00000003825	PTPN12	-1.077924472	0.000587287	0.004117365	up
865	ENSBTAG00000000197	TRMT10A	-1.449941013	0.000587641	0.004118132	up
866	ENSBTAG00000039509	GPRC5A	-1.206720263	0.000588243	0.004120638	up
867	ENSBTAG00000011572	TSPYL5	1.183210059	0.000589511	0.004127017	down
868	ENSBTAG00000009702	MYH8	1.254317189	0.000590134	0.004127017	down
869	ENSBTAG00000019746	CCDC66	-1.12996827	0.000594287	0.004149168	up
870	ENSBTAG00000010255	ZNF24	-1.074213484	0.000595555	0.004154574	up
871	ENSBTAG00000019182	TIA1	-1.343388099	0.000609098	0.004238519	up
872	ENSBTAG00000014255	VPS50	-1.065283017	0.000611421	0.004252926	up
873	ENSBTAG00000021249	LAT	1.377150065	0.000613569	0.004264343	down
874	ENSBTAG00000021577	ZFYVE16	-1.045461814	0.00061993	0.004301451	up
875	ENSBTAG00000000827	TAOK1	-1.261386553	0.000620536	0.004302843	up
876	ENSBTAG00000005784	CSMD2	1.487631098	0.000632134	0.004369927	down
877	ENSBTAG00000037819	SFT2D2	-1.217945444	0.0006368	0.004393166	up

878	ENSBTAG00000011833	GRIA3	-1.533876095	0.0006401	0.004403301	up
879	ENSBTAG00000045783	ATP5MD	-2.337418736	0.000642278	0.00441108	up
880	ENSBTAG00000014076	ABRAXAS1	-1.197472524	0.000645009	0.00442442	up
881	ENSBTAG00000014074	SNX13	-1.019829722	0.000649032	0.004439243	up
882	ENSBTAG00000016030	HOXD10	-1.640215729	0.000649542	0.004439243	up
883	ENSBTAG00000016374	OGFRL1	-1.1701679	0.00065228	0.004451041	up
884	ENSBTAG00000006824	APPL1	-1.120644167	0.000658342	0.004483008	up
885	ENSBTAG00000035615	UPF3B	-1.246181847	0.000659994	0.004492442	up
886	ENSBTAG00000053833	PARD3B	-1.500034429	0.000660641	0.004495028	up
887	ENSBTAG00000019354	PAX8	-2.890038677	0.000666866	0.004530178	up
888	ENSBTAG00000027246	Ubd	1.00441555	0.000668458	0.004530178	down
889	ENSBTAG00000023675	ZNF654	-1.003348464	0.000675987	0.004562572	up
890	ENSBTAG00000019924	DDHD1	-1.259709456	0.000677803	0.004571164	up
891	ENSBTAG00000005650	SKAP2	-1.152892303	0.000679877	0.004580788	up
892	ENSBTAG00000003943	TTC39B	-1.9308296	0.000680365	0.004581109	up
893	ENSBTAG00000003345	FAT4	-1.184135714	0.000686764	0.004618653	up
894	ENSBTAG00000051822	--	-2.263142517	0.000689229	0.004629682	up
895	ENSBTAG00000005137	LPGAT1	-1.398567024	0.000690574	0.004635017	up
896	ENSBTAG00000003726	MTMR9	-1.066571701	0.000700819	0.00469443	up
897	ENSBTAG00000045789	TNNT2	-1.083070008	0.000705202	0.004716284	up
898	ENSBTAG00000014890	CYP19A1	-4.19939191	0.000716577	0.004778288	up
899	ENSBTAG00000011602	RASAL3	1.224530276	0.000716743	0.004778288	down
900	ENSBTAG00000011642	RBM27	-1.048328885	0.000719598	0.004793523	up
901	ENSBTAG00000031194	PHLDA2	1.39913438	0.000720346	0.004794714	down
902	ENSBTAG00000014248	MIER3	-1.056944208	0.000733476	0.004864806	up
903	ENSBTAG00000039738	TMIGD3	1.343981555	0.000746489	0.004931689	down
904	ENSBTAG00000045886	STARD4	-2.592874368	0.000781277	0.005097523	up
905	ENSBTAG00000008605	DNAH2	-1.289302947	0.000802405	0.005207129	up
906	ENSBTAG00000017421	SLC3A1	-1.401143058	0.000808156	0.005238397	up
907	ENSBTAG00000020319	ALOX5	1.144911179	0.000814007	0.005270235	down
908	ENSBTAG00000021217	COL11A1	-2.509411468	0.000827936	0.005343979	up
909	ENSBTAG00000013163	ADAM33	-1.034819906	0.000830468	0.005354166	up
910	ENSBTAG00000030616	ZCCHC10	-1.50494184	0.000833058	0.005366753	up
911	ENSBTAG00000026172	LRATD2	-1.206107338	0.000836697	0.005386074	up
912	ENSBTAG00000020465	FERMT1	-1.640030745	0.000840344	0.005405424	up
913	ENSBTAG00000015718	CASP8	-1.084770103	0.00084588	0.005434804	up
914	ENSBTAG00000024420	COL28A1	-1.973344368	0.000846377	0.005435923	up
915	ENSBTAG00000019264	EDEM3	-1.020402009	0.000847676	0.005442189	up
916	ENSBTAG00000005102	PHTF2	-1.323012846	0.000856526	0.005480204	up
917	ENSBTAG00000015196	PHF6	-1.066827297	0.000883443	0.005620395	up
918	ENSBTAG00000000472	ZNF570	-1.641222513	0.000886093	0.005625057	up
919	ENSBTAG00000007479	CCDC90B	-1.114199678	0.000887363	0.005630445	up
920	ENSBTAG00000017123	ZNF354B	-1.669898934	0.000899109	0.005684019	up
921	ENSBTAG00000010913	SRXN1	-1.828306424	0.000901873	0.005694616	up

922	ENSBTAG00000023847	IKZF5	-1.023092737	0.000918143	0.005784338	up
923	ENSBTAG00000018942	SSX2IP	-1.186152477	0.000919826	0.005788512	up
924	ENSBTAG00000019881	ZNF474	2.644303468	0.000921163	0.005792525	down
925	ENSBTAG00000043958	TMEM33	-1.131234276	0.000927993	0.00582677	up
926	ENSBTAG00000010877	ARMC12	-1.062424407	0.000940908	0.005885915	up
927	ENSBTAG00000020878	DMTF1	-1.14839184	0.00099495	0.006157645	up
928	ENSBTAG00000021445	ST8SIA4	-1.832166677	0.000997266	0.006167446	up
929	ENSBTAG00000016507	SERHL2	1.533249213	0.001017622	0.006268014	down
930	ENSBTAG00000053546	PCDHAC2	-1.122432498	0.001044048	0.006405016	up
931	ENSBTAG00000011095	FAR2	-1.181765497	0.001056917	0.006458084	up
932	ENSBTAG00000023989	MSANTD4	-1.076354918	0.001079678	0.006573312	up
933	ENSBTAG00000017745	IL6ST	-1.262883342	0.001095834	0.006648455	up
934	ENSBTAG00000011363	EVI5	-1.239350347	0.001099299	0.006661845	up
935	ENSBTAG00000008243	LRRC40	-1.052421291	0.001099868	0.006662507	up
936	ENSBTAG00000018745	CEP290	-1.181826123	0.00110216	0.006669002	up
937	ENSBTAG00000004925	--	-4.380197781	0.001108705	0.00670398	up
938	ENSBTAG00000003456	ZNF606	-1.396734162	0.001128222	0.006792728	up
939	ENSBTAG00000017346	RASSF2	-2.041354946	0.001135542	0.006824602	up
940	ENSBTAG00000011241	HPDL	1.622357208	0.001150306	0.006898559	down
941	ENSBTAG00000026247	PKHD1L1	1.012803348	0.001167165	0.006984739	down
942	ENSBTAG00000004206	LRRC55	-2.090366233	0.001170775	0.007001369	up
943	ENSBTAG00000001042	MXD1	-1.002758793	0.001173138	0.007013008	up
944	ENSBTAG00000000013	PRKAA1	-1.000968216	0.001174011	0.007015739	up
945	ENSBTAG00000050479	SETMAR	-1.015735923	0.001209695	0.007190703	up
946	ENSBTAG00000014469	NBEAL1	-1.108246085	0.001221883	0.00725355	up
947	ENSBTAG00000050495	GREM1	-1.805235908	0.001238817	0.007332751	up
948	ENSBTAG00000007863	GLS	-1.322049071	0.001244006	0.007358294	up
949	ENSBTAG00000040072	ZNF140	-1.109982345	0.001250909	0.007386151	up
950	ENSBTAG00000000900	LRRC8C	-1.241576224	0.00125238	0.007392248	up
951	ENSBTAG00000009331	CPAMD8	1.145644366	0.001268706	0.007454646	down
952	ENSBTAG00000001517	KRT18	1.059450392	0.001280775	0.007504613	down
953	ENSBTAG00000012907	ODF2L	-1.241905689	0.001323655	0.007720939	up
954	ENSBTAG00000015716	ERO1A	-1.10428434	0.001325845	0.007725685	up
955	MSTRG.11971	--	-2.014167332	0.001326422	0.007726373	up
956	ENSBTAG00000010118	HAT1	-1.007545816	0.001344421	0.007812302	up
957	ENSBTAG00000049723	DENR	-1.354227699	0.001357169	0.007864671	up
958	ENSBTAG00000032933	TTC23L	-1.583463597	0.001361244	0.00787761	up
959	ENSBTAG00000008006	RASGRP3	-1.220276461	0.001368401	0.007905274	up
960	ENSBTAG00000003495	KDM7A	-1.171777291	0.001384342	0.007972747	up
961	ENSBTAG00000037393	ZNF639	-1.005501017	0.001389277	0.007994484	up
962	ENSBTAG00000010736	BBS5	1.178477803	0.001402966	0.008041544	up
963	ENSBTAG00000010350	USP53	-1.003490393	0.001402972	0.008041544	up
964	ENSBTAG00000014659	NEMP1	-1.12661684	0.001405729	0.008051865	up
965	ENSBTAG00000013185	TIMD4	1.587374363	0.001407743	0.008060659	down

966	ENSBTAG00000015769	VSIG4	1.155840579	0.001411622	0.008077376	down
967	ENSBTAG00000015151	FOXN2	-1.199853306	0.001427119	0.008143912	up
968	ENSBTAG00000005547	VCPKMT	-1.811153762	0.00143062	0.008160942	up
969	ENSBTAG00000021444	TWIST2	-1.854816856	0.001453335	0.008262717	up
970	ENSBTAG00000011498	ZNF184	-1.246996682	0.001472806	0.008338267	up
971	ENSBTAG00000016932	SENP7	-1.152505687	0.001478212	0.008358169	up
972	ENSBTAG00000038128	BOLA-DQA5	8.045486656	0.001482962	0.008377418	down
973	ENSBTAG00000020914	CPNE8	-1.054384816	0.001487557	0.008389301	up
974	ENSBTAG00000003384	CEP162	-1.611406169	0.001523251	0.008539131	up
975	ENSBTAG00000045617	MTERF1	-1.220830566	0.00152924	0.008566998	up
976	ENSBTAG00000048962	--	-2.093013067	0.001538591	0.00861079	up
977	ENSBTAG00000001533	HACE1	-1.189424738	0.001540982	0.008621305	up
978	ENSBTAG00000024751	ULBP3	1.348620359	0.00154954	0.00866343	down
979	ENSBTAG00000051482	CFDP2	-1.974474521	0.001555254	0.008691683	up
980	ENSBTAG00000019302	BCL2	1.99199162	0.001556142	0.008691683	up
981	ENSBTAG00000049002	ClD	-1.029360115	0.001557733	0.008694802	up
982	ENSBTAG00000009634	HOXC5	1.191109479	0.00157469	0.008766209	down
983	ENSBTAG00000021962	BCLAF3	-1.030664531	0.001577199	0.008777275	up
984	ENSBTAG00000016046	GOPC	1.007521399	0.001590428	0.008842127	up
985	ENSBTAG00000019300	TPMT	1.270595092	0.001603182	0.008894092	up
986	ENSBTAG00000007666	IGF2BP2	-1.286956467	0.001609776	0.008920243	up
987	ENSBTAG00000054229	C2CD3	-1.242495874	0.00167204	0.009216743	up
988	ENSBTAG00000013069	IQCB1	1.081774196	0.00167675	0.00923002	up
989	ENSBTAG00000053124	pol	-1.137742885	0.00168716	0.009269749	up
990	ENSBTAG00000032982	TERF1	-1.139388278	0.001690644	0.009285862	up
991	ENSBTAG00000004426	DNAJB14	-1.954931495	0.001691345	0.009286678	up
992	ENSBTAG00000009789	GNAQ	-1.436452789	0.001692943	0.009292425	up
993	ENSBTAG00000021948	ZMYM6	-1.010615417	0.00170331	0.009334117	up
994	ENSBTAG00000047448	C25H16orf54	1.082386794	0.001755904	0.009563202	down
995	ENSBTAG00000006918	TLK1	-1.006438842	0.001770554	0.009624315	up
996	ENSBTAG00000030960	DNAJC25	-1.357783484	0.001789769	0.009709958	up
997	ENSBTAG00000019459	CHAC2	-1.143255542	0.001800014	0.00975297	up
998	ENSBTAG00000005978	PIK3R5	1.128790622	0.001829334	0.009886387	down
999	ENSBTAG00000014791	CTH	-1.231841052	0.001843898	0.009948986	up
1000	ENSBTAG00000009023	TMTC3	-1.215876935	0.001854565	0.009990684	up
1001	ENSBTAG00000016484	ATP11C	-1.144765427	0.001856307	0.009996869	up
1002	ENSBTAG00000004964	PCGF5	-1.174534426	0.001880377	0.010100653	up
1003	ENSBTAG00000006730	SUSD2	1.128188269	0.001891828	0.010151297	down
1004	ENSBTAG00000048516	pol	-2.214656773	0.001932744	0.010335791	up
1005	ENSBTAG00000004575	FAM126A	-1.109687663	0.001947592	0.010383017	up
1006	ENSBTAG00000055051	RNF125	-1.828871307	0.001965043	0.010465315	up
1007	ENSBTAG00000001574	GPATCH2	-1.436235535	0.001983386	0.010546327	up
1008	ENSBTAG00000016430	METTL8	-1.068004107	0.001986249	0.010554885	up
1009	ENSBTAG00000004995	C1GALT1	-1.232824895	0.001990367	0.010568203	up

1010	ENSBTAG00000052620	--	-1.523413876	0.001991341	0.010568606	up
1011	ENSBTAG00000019232	TYW5	-1.534820223	0.00199997	0.010604375	up
1012	ENSBTAG00000012995	MCUB	-1.224267391	0.002020124	0.010684324	up
1013	ENSBTAG00000054797	STAG1	-1.458888537	0.002050015	0.010805405	up
1014	ENSBTAG00000011636	DDIAS	-2.578743978	0.002061886	0.010854066	up
1015	ENSBTAG00000019250	BTK	1.119542054	0.002065685	0.010869524	down
1016	ENSBTAG00000008925	MRE11	-1.26142547	0.002117902	0.011094642	up
1017	ENSBTAG00000013242	MYMK	-1.369470027	0.002118128	0.011094642	up
1018	ENSBTAG00000049238	CFDP2	-1.529527584	0.002141653	0.01119697	up
1019	ENSBTAG00000010387	MINDY3	-1.03659847	0.002155798	0.011256941	up
1020	ENSBTAG00000011667	PM20D2	-1.111388272	0.002170502	0.01132319	up
1021	ENSBTAG00000007681	DHFR	-1.036840763	0.00221708	0.011502044	up
1022	MSTRG.11341	--	-1.044664626	0.002242729	0.011600338	up
1023	ENSBTAG00000004023	ELAPOR2	-2.257511366	0.002261522	0.011675056	up
1024	ENSBTAG00000017741	HACD4	-1.689064519	0.002266136	0.011695287	up
1025	ENSBTAG00000019137	SCN7A	-1.712560281	0.002286497	0.011782314	up
1026	ENSBTAG00000017694	TRPS1	-1.022208888	0.002310316	0.011883238	up
1027	ENSBTAG00000008771	MYEF2	-1.436720029	0.002367872	0.012123735	up
1028	ENSBTAG00000021582	NCAPG	-1.783646495	0.002385612	0.012199731	up
1029	MSTRG.751	--	1.147451315	0.002393893	0.012230936	down
1030	ENSBTAG00000000322	CD2AP	-1.229893702	0.002449912	0.012456679	up
1031	ENSBTAG00000011829	NF1	-1.395366592	0.002463384	0.012510069	up
1032	ENSBTAG00000010741	KLHL41	-1.03229358	0.002490865	0.012623176	up
1033	ENSBTAG00000006167	BTBD8	-1.834233823	0.002515907	0.012734557	up
1034	ENSBTAG00000005633	ARHGEF28	-1.145005367	0.002534573	0.012803527	up
1035	ENSBTAG00000030340	IFFO2	1.031026896	0.002596637	0.013033491	down
1036	ENSBTAG00000000284	NAALAD2	-3.231065296	0.002633684	0.013180161	up
1037	ENSBTAG00000001393	MTF2	-1.090935033	0.002637515	0.013191494	up
1038	ENSBTAG00000019174	ZNF710	1.201741449	0.002638639	0.013193194	down
1039	MSTRG.6775	--	-1.393621544	0.002642625	0.013209205	up
1040	ENSBTAG00000019864	MAPK15	1.157991671	0.002667143	0.013300173	down
1041	ENSBTAG0000001223	CAAP1	-1.04548775	0.002734208	0.013550644	up
1042	ENSBTAG00000017256	CD2	1.317245536	0.002824892	0.013909702	down
1043	ENSBTAG00000048649	F52C9.6	-1.10849463	0.002828381	0.013918222	up
1044	ENSBTAG00000023054	SLC25A16	-1.728278577	0.002860075	0.01402553	up
1045	ENSBTAG00000046362	BBIP1	-1.10667432	0.002893268	0.014143002	up
1046	ENSBTAG00000016260	LRRK2	-1.014451156	0.00290791	0.014184652	up
1047	ENSBTAG00000003632	CYP39A1	-2.037419161	0.002916908	0.014213176	up
1048	ENSBTAG00000026080	LAIR1	1.245346406	0.002916908	0.014213176	down
1049	ENSBTAG00000054887	ZNF471	-1.048126378	0.002984043	0.014473298	up
1050	ENSBTAG00000015481	MTIF2	-1.276784214	0.003002604	0.014529841	up
1051	ENSBTAG00000052702	COLCA2	1.687397934	0.003020228	0.014585789	down
1052	ENSBTAG00000022689	RYBP	-1.087999179	0.0030289	0.014623476	up
1053	ENSBTAG00000002281	ZNF354C	-2.063009798	0.003060102	0.014735816	up

1054	ENSBTAG00000010529	FZD6	-1.037126791	0.003085007	0.014842391	up
1055	MSTRG.2022	--	1.204140717	0.003085701	0.014842391	down
1056	ENSBTAG00000006377	MYO1G	1.046659674	0.003103431	0.01492341	down
1057	ENSBTAG00000014711	DBF4	-1.732066795	0.003163334	0.015168149	up
1058	ENSBTAG00000014407	INO80D	-1.207601894	0.003200581	0.015303176	up
1059	ENSBTAG00000007942	TENT4B	-1.085216002	0.003304983	0.015671652	up
1060	ENSBTAG00000053347	PRUNE2	-2.276375943	0.003331779	0.015773728	up
1061	ENSBTAG00000026842	ZRANB3	-1.328142153	0.003367166	0.01590103	up
1062	ENSBTAG00000015385	CTDSPL2	-1.049629329	0.003382091	0.015944686	up
1063	ENSBTAG00000008363	EXD1	-7.325530332	0.00341535	0.01606999	up
1064	ENSBTAG00000008773	GUCY2D	2.30133416	0.00344281	0.016185629	down
1065	ENSBTAG00000004085	ASF1B	1.108398949	0.003482107	0.016333895	down
1066	ENSBTAG00000018031	PODXL2	1.238307968	0.00353144	0.016519291	down
1067	ENSBTAG00000006734	BICD1	-2.277984747	0.003549326	0.016589136	up
1068	ENSBTAG00000040406	OBI1	2.503631423	0.003597897	0.016738218	down
1069	ENSBTAG00000004154	SKIL	-1.151696626	0.003629361	0.016846316	up
1070	ENSBTAG00000002240	GPR18	3.949016071	0.003638014	0.016867887	down
1071	ENSBTAG00000000586	CPLANE1	-1.269186633	0.003666007	0.016946351	up
1072	ENSBTAG00000027809	RABGAP IL	-1.62611064	0.003673739	0.016968975	up
1073	ENSBTAG00000011959	GPR19	-1.673371475	0.003674932	0.016968975	up
1074	ENSBTAG00000010841	FMO5	-1.06545944	0.003716397	0.017113515	up
1075	ENSBTAG00000025221	TEX30	-1.092118202	0.0037474	0.017237424	up
1076	ENSBTAG00000004813	SUSD1	-6.87282876	0.003761211	0.017286788	up
1077	ENSBTAG00000005951	HIC2	1.010708713	0.003771389	0.017324111	down
1078	ENSBTAG00000005629	CRYBG2	2.101719921	0.003792902	0.017398418	down
1079	ENSBTAG00000014171	NAPEPLD	-1.199524341	0.003804926	0.01743536	up
1080	ENSBTAG00000049052	pol	-3.181623523	0.003840015	0.017581722	up
1081	ENSBTAG00000008909	PNPT1	-1.069765595	0.003844803	0.017594151	up
1082	ENSBTAG00000007837	-	-1.297277481	0.003902933	0.017792472	up
1083	ENSBTAG00000048620	CEBPZOS	-1.064237617	0.003939832	0.017921873	up
1084	ENSBTAG00000052991	Pol	-1.101185411	0.003956332	0.017987214	up
1085	ENSBTAG00000009014	UPK1B	6.773468928	0.003962098	0.018003704	down
1086	ENSBTAG00000010388	MGAT4A	-1.156672595	0.004004824	0.018143998	up
1087	ENSBTAG00000017215	WDR76	-1.154536559	0.004043407	0.018289274	up
1088	ENSBTAG00000054599	ERVPABLB-1	2.30256277	0.00408972	0.018444266	down
1089	ENSBTAG00000017133	GINS4	1.560969645	0.004097015	0.018462332	down
1090	ENSBTAG00000002253	FKBP6	3.723064777	0.004105434	0.018480488	down
1091	ENSBTAG00000013455	SPOPL	-1.058474657	0.004134552	0.018566894	up
1092	ENSBTAG00000044063	B4GALT6	-1.519852404	0.004180058	0.018730227	up
1093	ENSBTAG00000008140	FAP	-1.027868116	0.004189247	0.018762476	up
1094	ENSBTAG00000055011	FAM200B	-1.91694426	0.00419168	0.018768384	up
1095	ENSBTAG00000005833	ETNK1	-1.380456168	0.004248414	0.018956886	up
1096	ENSBTAG00000023832	ADAM8	1.121230745	0.004255112	0.018981744	down
1097	ENSBTAG00000050912	Pol	-1.792809985	0.004262509	0.018999645	up

1098	ENSBTAG00000010371	CHAC1	1.938051527	0.004292219	0.019101735	down
1099	ENSBTAG00000051479	GLS	-1.433833	0.004305643	0.019156416	up
1100	MSTRG.14107	Rxl	-1.286734834	0.004313061	0.019182464	up
1101	ENSBTAG00000021051	ZNHIT6	-1.040699055	0.004337261	0.019266547	up
1102	ENSBTAG00000002744	MUSK	-1.048050139	0.004383503	0.019436067	up
1103	ENSBTAG00000003051	FER	-1.036612107	0.004409552	0.019534293	up
1104	ENSBTAG00000015728	NSUN3	-1.074572966	0.004468659	0.019740876	up
1105	ENSBTAG00000025622	DEF6	1.191892782	0.00447618	0.019768912	down
1106	ENSBTAG00000002291	ZBTB41	-1.186828533	0.004492678	0.019826273	up
1107	ENSBTAG00000052213	ADIPOQ	-1.278461211	0.004496231	0.019826273	up
1108	ENSBTAG00000007866	HS3ST3B1	-2.777607579	0.004560275	0.020045689	up
1109	ENSBTAG00000021156	TBCID31	-1.097266905	0.004562923	0.020051103	up
1110	ENSBTAG00000016473	NAT1	-1.168196602	0.004576735	0.020091817	up
1111	ENSBTAG00000005870	FANCL	-1.518988463	0.00463552	0.020275884	up
1112	ENSBTAG00000001034	IL18R1	1.768065791	0.004677711	0.020407423	down
1113	ENSBTAG00000015749	STEAP1	-3.124695747	0.004683551	0.020417033	up
1114	ENSBTAG00000025856	JMY	-1.043664195	0.004732083	0.020575338	up
1115	ENSBTAG00000007746	AHR	-1.0096796	0.004776299	0.020719444	up
1116	ENSBTAG00000033267	LIN9	-1.249660064	0.004889856	0.021103325	up
1117	MSTRG.3571	--	-9.166916252	0.004907432	0.021168328	up
1118	ENSBTAG00000030929	ZNF558	-1.268640222	0.004950204	0.02131461	up
1119	ENSBTAG00000013918	ADGRL3	-1.456039891	0.004964857	0.021350408	up
1120	ENSBTAG00000003668	RADX	-3.061296179	0.005017178	0.021531221	up
1121	ENSBTAG00000030470	ZNF160	-1.367162103	0.005019618	0.021531221	up
1122	ENSBTAG00000003506	STEAP2	-2.752964165	0.00507398	0.021686758	up
1123	ENSBTAG00000019639	SNX16	-1.162705685	0.005075844	0.02168922	up
1124	ENSBTAG00000008703	EIF2AK2	-1.259300809	0.005091703	0.021743754	up
1125	MSTRG.9830	--	-1.102907602	0.005109466	0.021777626	up
1126	ENSBTAG00000038486	B3GALT9	-4.033423002	0.005151726	0.021929995	up
1127	ENSBTAG00000010884	NKAP	-1.075590185	0.00517284	0.021986994	up
1128	ENSBTAG00000053029	ZNF26	-1.35687516	0.005209985	0.022116455	up
1129	ENSBTAG00000005025	FAM91A1	-1.006654273	0.005211288	0.022116455	up
1130	ENSBTAG00000021672	RGS1	-1.048170654	0.005226702	0.022159531	up
1131	ENSBTAG00000009154	U2SURP	-1.20801375	0.005255357	0.022258604	up
1132	ENSBTAG00000005110	CADPS2	-1.37781 7853	0.005259717	0.022271471	up
1133	ENSBTAG00000006232	WDR86	2.009794301	0.005263695	0.022282709	down
1134	ENSBTAG00000009994	EML5	-1.776658125	0.005358037	0.022636254	up
1135	ENSBTAG00000001786	HYKK	-1.067164804	0.005366529	0.022661068	up
1136	ENSBTAG00000036260	LPXN	1.161741874	0.005437239	0.022885053	down
1137	ENSBTAG00000010210	SUV39H2	-1.480879165	0.005512394	0.023126237	up
1138	ENSBTAG00000047700	GHA2	2.266447601	0.005521462	0.02314698	down
1139	ENSBTAG00000019895	PIBF1	-1.074613061	0.005550987	0.023253388	up
1140	ENSBTAG00000000671	PARP3	-1.09494105	0.005571391	0.023298291	up
1141	ENSBTAG00000033214	TBC1D19	-1.047918554	0.005578917	0.023323971	up

1142	ENSBTAG00000036087	ARMC2	-1.377092695	0.005581694	0.023324894	up
1143	ENSBTAG00000020802	DNAH12	-1.106160459	0.005587539	0.023342635	up
1144	ENSBTAG00000008661	C15H11orf52	-1.067911138	0.005664125	0.023563208	up
1145	ENSBTAG00000013734	ATP6V1E2	1.63352721	0.005734976	0.023816769	down
1146	MSTRG.9599	Antxrl	-3.31140405	0.005746444	0.02384528	up
1147	ENSBTAG00000025200	ASIC2	1.532495081	0.005757229	0.023879674	down
1148	ENSBTAG00000040253	ADRA1B	2.876341532	0.005784054	0.023955602	down
1149	ENSBTAG00000034154	AGMO	1.285974571	0.005821169	0.024091543	down
1150	ENSBTAG00000007175	AVPRIA	1.174497731	0.00585149	0.024181373	down
1151	ENSBTAG00000014016	KZF1	2.041169189	0.005926201	0.02430165	down
1152	ENSBTAG00000019755	REEP3	1.225873915	0.005962695	0.0245 32.565	up
1153	MSTRG.1259		943.27741	0.005973646	0.024571618	down
1154	ENSBTAG00000008048	GCFC2	1.2899091 83	0.005 996594	0 024635923	up
1155	ENSBTAG00000024534	POU2F1	-1.218553528	0.006017412	0.024685314	up
1156	ENSBTAG00000014599	LRRC66	-1.377413427	0.006036385	0.024745066	up
1157	ENSBTAG00000053874	NATSL	2 150656668	0.006085995	0.0248 69 728	down
1158	ENSBTAG00000021568	VWCE	699-49 08 89	0.006087661	0.0248 70502	down
1159	ENSBTAG00000004939	ZNF569	-1.162686175	0.006132454	0.025023147	up
1160	ENSBTAG00000018324	AGO3	-1.31141147	0006142417	0.025057728	up
1161	ENSBTAG00000003961	SNX20	49171309	0.006.233828	0.025354719	down
1162	ENSBTAG00000034368	PR.SS33	-2.161966416	0.006236297	0.0.25354719	up
1163	ENSBTAG00000002614	RUFY2	-1.103093493	0.006243-403	0 0253 77483	up
1164	ENSBTAG00000031687	ZNF146	-1.013622369	0.006353819	0.025725348	up
1165	ENSBTAG00000009599	CNI	1.87288:8082	0.006546511	0.0263-4247	up
1166	ENSBTAG00000000287	GNPDA2	-1.003-41 9923	0.006584586	0.026470381	up
1167	ENSBTAG00000012997	SMPDL3B	2. 199 752568	0.006627726	0.0266 12044	down
1168	ENSBTAG00000004008	FAM199X	-1.434067105	0.006652425	0.026683932	up
1169	ENSBTAG00000021752	DNAB4	-1.020171652	0.006 701.266	0.026843319	up
1170	ENSBTAG00000005826	PTARI	-1.025094167	0.006744929	0.026955969	up
1171	ENSBTAG00000005026	MANEA	-1.431656706	0.00677047	0.027030516	up
1172	ENSBTAG00000040209	ZNF112	-1.10306-4935	0.006832947	0.027208994	up
1173	ENSBTAG00000033344	SENPS	-1.53481 7489	0.007042387	0.0278 8633	up
1174	ENSBTAG00000009879	PCGF6	-1.111203945	0.007080976	0.027991436	up
1175	ENSBTAG00000012434	ENOXI	-1.003570145	0.007120777	0.028115801	up
1176	ENSBTAG00000006197	ZDHHC21	-1.263.297455	0.00728898.2	0.029658992	up
1177	ENSBTAG00000025442	HSPAIL	-1.2395705 73	0.007389492	0.028966.26	down
1178	ENSBTAG00000010765	C28H1 orf131	-1.017674019	0.007458969	0.029163927	up
1179	ENSBTAG00000054372	ERVPABLB-1	4.371.21 8845	0.007466275	0.029173618	down
1180	ENSBTAG00000055151		-3.289 206462	0.007564262	0.029459336	up
1181	ENSBTAG00000010634	NDNF	1.1740294	0.007648274	0.029710942	up
1182	ENSBTAG00000031242	CDCA4	806640593	0.007676514	0.029786183	down
1183	ENSBTAG00000016385	F10	99845 7835	0.007695 738	0.029847127	down
1184	ENSBTAG00000000868	ZFP1	-1.610794794	0.007706541	0.029861516	up
1185	ENSBTAG00000051886	Kiaa 1109	-1.447843644	0.007803 107	0.0301-46304	up

1186	ENSBTAG00000030193	GPR.35	393 186778	0.007905-496	0.030147834	down
1187	ENSBTAG00000006256	PTPRO	927928585	0.007S4089	0.0302 4985	down
1188	ENSBTAG00000001474	NRGN	845761305	0.007864645	0.030320655	down
1189	ENSBTAG00000015073	CRPPA	-1.250811619	0.007879419	0 0303 70662	up
1190	ENSBTAG00000010991	TIR	-1.695589666	0.007928229	0.0305 24 395	up
1191	ENSBTAG00000000161	TMEM40	-1.3.5960887	0.007993 724	0.030712825	up
1192	ENSBTAG00000009169	XPR1	-1.038151938	0.008017508	0.030783137	up
1193	MSTRG.8189	--	2 957029961	0.00814219	0.031183645	down
1194	ENSBTAG00000006587	ZNF367	-1.384287167	0.008 234652	0.031444795	up
1195	ENSBTAG00000053998	--	-1.499503547	0.008.298.243	0.031644567	up
1196	ENSBTAG00000047707	BOLA-DOB	063665052	0.008443445	0.032082044	down
1197	ENSBTAG00000048942	F52C9.6	-1.35928884	0: 008/657594	0 032740597	up
1198	ENSBTAG00000031579	SGO2	-1.031071446	0.0086619	0.032742178	up
1199	ENSBTAG00000052696	CFDP2	-1 469 799699	0.008723611	0.032931095	up
1200	FNSBTAG00000018133	SEMA3A	6.357552005	0.008778251	0.033095315	down
1201	ENSBTAG00000003861	GPATCH11	-1.0368343	0.008792845	0.03313303	up
1202	MSTRG.8337		1.539162873	0.00884219	0.033289176	down
1203	ENSBTAG00000005394	COX18	-1.113301272	0.008981698	0.033731442	up
1204	ENSBTAG00000024275	XRCC4	-1.094611092	0.009005228	0.033801811	up
1205	ENSBTAG00000021869	THAP5	-1.016915159	0.009064957	0.0339835	up
1206	ENSBTAG00000014046	BPI	2.098647588	0.009092645	0.034056225	down
1207	ENSBTAG00000017370	ARFGEF3	-1.106106457	0.009165017	0.034282293	up
1208	ENSBTAG00000053260	F52C9.6	-1.953888075	0.009200367	0.034375142	up
1209	ENSBTAG00000021991	TMEFF1	-1.373386963	0.009246583	0.03449545	up
1210	ENSBTAG00000007388	ZC3H12D	1.508924008	0.009258246	0.034531308	down
1211	ENSBTAG00000000195	ZNF548	-1.064220979	0.009288263	0.034620263	up
1212	ENSBTAG00000008553	B4GALNT3	-1.086315569	0.009405369	0.034994787	up
1213	ENSBTAG00000017263	MXI1	-1.129167137	0.009497197	0.035243013	up
1214	ENSBTAG00000046396	TMEM200C	1.218131181	0.009665154	0.035708907	down
1215	ENSBTAG00000049591	Itsn2	-1.450194726	0.009684005	0.035762862	up
1216	MSTRG.6298		-1.463457014	0.009733918	0.035915683	up
1217	ENSBTAG00000009265	NR5A2	-2.429478397	0.009795975	0.036120915	up
1218	ENSBTAG00000020956	MPPED2	1.319394828	0.009828383	0.036224551	down
1219	ENSBTAG00000014969	CILP2	-2.05626822	0.009872125	0.036336563	up
1220	ENSBTAG00000002600	EFCAB7	-1.362570079	0.010328131	0.037589309	up
1221	ENSBTAG00000007329	SETDB2	-1.294599721	0.010471679	0.037972237	up
1222	ENSBTAG00000005439	FAM102B	-1.395928676	0.010529777	0.038141845	up
1223	ENSBTAG00000003305	NCF1	1.086865081	0.010603454	0.038331412	down
1224	ENSBTAG00000053939	F52C9.6	-2.457507255	0.010604872	0.038331412	up
1225	ENSBTAG00000004475	CDK5R1	-1.072738745	0.010647584	0.038444536	up
1226	ENSBTAG00000001847	CNTLN	-1.524400543	0.010670395	0.038490637	up
1227	ENSBTAG00000053836	CCDC71L	-1.200776838	0.010705688	0.038563378	up
1228	ENSBTAG00000011932	PRG4	-1.312764673	0.010752584	0.038707463	up
1229	ENSBTAG00000048331	ACAD10	-1.1026786	0.010766636	0.038749764	up

1230	ENSBTAG00000004136	NFE2L3	-1.385534061	0.010790515	0.038795072	up
1231	ENSBTAG00000021600	KANSL1L	-1.018678803	0.011044863	0.039539859	up
1232	ENSBTAG00000017375	FAM241A	-1.056106851	0.011079613	0.039638982	up
1233	ENSBTAG00000017624	RAB17	1.198221973	0.011130047	0.039777163	down
1234	ENSBTAG00000049540	CCDC103	-3.698076581	0.011134993	0.039786397	up
1235	ENSBTAG00000011836	OMD	-2.313022542	0.011141322	0.039792125	up
1236	ENSBTAG00000027321	CCDC18	-1.530874985	0.011148863	0.039810613	up
1237	ENSBTAG00000035018	ZCCHC12	-1.275813356	0.011161053	0.039834027	up
1238	ENSBTAG00000010774	NUSAP1	-1.474470413	0.011283603	0.040164	up
1239	ENSBTAG00000015338	DSCC1	-1.0228763	0.01129554	0.040181008	up
1240	ENSBTAG00000023814	ECT2	-2.001741334	0.011309254	0.040204316	up
1241	ENSBTAG00000007543	TAF1B	-1.082771466	0.011381156	0.040408742	up
1242	ENSBTAG00000000715	GIMAP5	2.2206698	0.01140732	0.040458985	down
1243	ENSBTAG00000043964	ARL5B	-1.345518298	0.011445415	0.040551397	up
1244	ENSBTAG00000013483	TAF1A	-1.014528774	0.011506868	0.040719646	up
1245	ENSBTAG00000025775	INSL3	-3.897580123	0.011519562	0.040744816	up
1246	ENSBTAG00000030543	SLC6A16	6.409390936	0.011551645	0.040816134	down
1247	ENSBTAG00000020250	SLC28A1	-2.349149564	0.011762341	0.041438877	up
1248	ENSBTAG00000019772	OXTR	2.035355276	0.01179858	0.041549161	down
1249	ENSBTAG00000018151	HELQ	-1.327840788	0.011822708	0.041612524	up
1250	ENSBTAG00000006223	THEMIS2	1.378795786	0.011845579	0.041662397	down
1251	ENSBTAG00000009289	MS4A8	1.614306462	0.011916981	0.041834887	down
1252	ENSBTAG00000020233	CCDC186	-1.03167222	0.012015732	0.042111326	up
1253	ENSBTAG00000006107	SLF1	-1.168413247	0.012092598	0.042336663	up
1254	MSTRG.7839	--	-1.040220082	0.012159364	0.042517377	up
1255	ENSBTAG00000014111	NPP4B	-1.07978551	0.012473958	0.043410094	up
1256	ENSBTAG00000050709	DIAPH2	-1.597069104	0.012596061	0.043717572	up
1257	ENSBTAG00000049215	SHISA3	-1.023963243	0.012601556	0.043718624	up
1258	ENSBTAG00000020654	BAZ2B	-1.213793665	0.012611718	0.043729039	up
1259	ENSBTAG00000018524	AMHR2	-2.682259702	0.012855884	0.044422118	up
1260	ENSBTAG00000013429	CLEC4G	1.111950794	0.012886783	0.044497318	down
1261	ENSBTAG00000006546	GSTA2	2.214257108	0.013142553	0.045174987	down
1262	ENSBTAG00000005062	TEC	-1.643983015	0.013269133	0.045500346	up
1263	ENSBTAG00000000998	SLC39A6	-1.051825723	0.013293659	0.045553146	up
1264	ENSBTAG00000046257	GIMAP4	1.205759033	0.013501274	0.0461273	down
1265	ENSBTAG00000053134	Pol	-1.682894289	0.013542691	0.046212547	up
1266	ENSBTAG00000005496	RP2	-1.610221729	0.013664607	0.046543453	up
1267	ENSBTAG00000052813	Pol	-1.254726381	0.013667303	0.046543453	up
1268	MSTRG.1196	cs	-2.012655301	0.013923525	0.047234519	up
1269	ENSBTAG00000017670	GBP3	-1.015818541	0.013942538	0.047279973	up
1270	ENSBTAG00000006388	FBXO45	-1.050744141	0.01406985	0.047598286	up
1271	ENSBTAG00000052005	ERVPABLB-1	7.442943496	0.014102481	0.047697486	down
1272	ENSBTAG00000020739	NXT2	-1.12072026	0.014110338	0.047714477	up
1273	ENSBTAG00000013231	C8H9orf40	-1.080323383	0.014206508	0.047991492	up

1274	ENSBTAG00000050420	SIGLEC14	1.506475705	0.014301023	0.048210084	down
1275	ENSBTAG00000020283	DUSP5	-1.027318837	0.014353046	0.048320515	up
1276	ENSBTAG00000054341	--	-1.323488609	0.014396287	0.048408615	up
1277	ENSBTAG00000000621	CATSPERG	-1.574424141	0.01441921	0.048457292	up
1278	ENSBTAG00000012099	TMEM87B	-1.063872183	0.014482922	0.048632582	up
1279	ENSBTAG00000014484	TMEM26	-2.193210878	0.014490655	0.048639153	up
1280	MSTRG.2347	--	-1.732716121	0.014583885	0.048874155	up
1281	ENSBTAG00000047083	FJX1	1.07840157	0.014727441	0.04919368	down
1282	ENSBTAG00000003444	SYDE2	1.316966112	0.014747589	0.049230371	up
1283	ENSBTAG00000054660	CFDP2	-1.17445154	0.014793413	0.049350452	up
1284	ENSBTAG00000011197	IPMK	-1.519034791	0.014841554	0.049481642	up
