# Supplementary material for: Molecular regulatory mechanisms of dietary supplementation with Allium mongolicum Regel powder to improve muscle development and meat quality in Angus calves
Source: Anim Biosci. 2025 Feb 27;38(8):1798–816. doi: 10.5713/ab.24.0809 (PMC12229934; doi:10.5713/ab.24.0809)
Supplement: Supplementary file 3 [file ab-24-0809-Supplementary-3.pdf]

Supplement 3. The top 20 enriched terms in GO analysis of DEGs between CON and HAMR group

ID	class	Description	*P*-value	Q-value
GO:0005488	Molecular Function	binding	2.31E-13	2.99E-10
GO:0044424	Cellular Component	intracellular part	9.66E-13	4.00E-10
GO:0005622	Cellular Component	intracellular	9.95E-13	4.00E-10
GO:0043231	Cellular Component	intracellular membrane- bounded organelle	3.85E-12	8.72E-10
GO:0043227	Cellular Component	membrane- bounded organelle	4.34E-12	8.72E-10
GO:0005634	Cellular Component	nucleus	5.30E-11	8.52E-09
GO:0043226	Cellular Component	organelle	3.27E-10	3.95E-08
GO:0043229	Cellular Component	intracellular organelle	3.44E-10	3.95E-08
GO:0044422	Cellular Component	organelle part	1.11E-09	1.12E-07
GO:0044428	Cellular Component	nuclear part	1.58E-09	1.41E-07
GO:0044446	Cellular Component	intracellular organelle part	2.22E-09	1.78E-07
GO:0031981	Cellular Component	nuclear lumen	3.32E-09	2.36E-07
GO:0043233	Cellular Component	organelle lumen	3.53E-09	236E-07
GO:0070013	Cellular Component	intracellular organelle lumen	4.88E-09	3.02E-07
GO:0031974	Cellular Component	membrane- enclosed human	8.20E-09	4.71E-07
GO:0005654	Cellular Component	nucleoplasm	4.10E-08	2.20E-06
GO:0005515	Molecular Function	protein binding	3.33E-08	2.15E-05
GO:0005623	Cellular Component	cell	2.24E-06	0.000105799
GO:0044464	Cellular Component	cell part	2.24E-06	0.000105799
GO:0015630	Cellular Component	microtubule cytoskeleton	3.79E-06	0.000169104
