# Supplementary material for: Molecular regulatory mechanisms of dietary supplementation with Allium mongolicum Regel powder to improve muscle development and meat quality in Angus calves
Source: Anim Biosci. 2025 Feb 27;38(8):1798–816. doi: 10.5713/ab.24.0809 (PMC12229934; doi:10.5713/ab.24.0809)
Supplement: Supplementary file 4 [file ab-24-0809-Supplementary-4.pdf]

Supplement 4. The enriched terms in KEGG pathway analysis of DEGs between CON and HAMP group

Pathway	Pathway _ID	KEGG_A_ class	KEGG_B_ class	*P*- va1ue
Hypertrophic cardiomyopathy	ko05410	Human Diseases	Cardiovascular disease	0.00024515
Osteoclast differentiation	ko04380	Organismal Systems	Development and regeneration	0.00050221
Adrenergic signaling in cardiomyocytes	ko04261	Organismal Systems	Circulatory system	0.00058519
Toxoplasmosis	ko05145	Human Diseases	Infectious disease: parasitic	0.00064762
Herpes simplex virus 1 infection	ko05168	Human Diseases	Infectious disease: viral	0.00067012
Sphingolipid signaling pathway	ko04071	Environmental Information Processing	Signal transduction	0.00199174
Fc epsilon RI signaling pathway	ko04664	Organismal Systems	Immune system	0.00530965
Focal adhesion	ko04510	Cellular Processes	Cellular community- eukaryotes	0.00544123
Adherents junction	ko04520	Cellular Processes	Cellular community- eukaryotes	0.00757335
Homologous recombination	ko03440	Genetic Information Processing	Replication and repair	0.00761541
Signaling pathways regulating pluripotency of stem cells	ko04550	Cellular Processes	Cellular community- eukaryotes	0.00829481
MAPK signaling pathway	ko04010	Environmental Information Processing	Signal transduction	0.00966688
B cell receptor signaling pathway	ko04662	Organismal Systems	Immune system	0.01195894
Circadian rhythm	ko04710	Organismal Systems	Environmental adaptation	0.01201675
Endocytosis	ko04144	Cellular Processes	Transport and catabolism	0.01219538
TNF signaling pathway	ko04668	Environmental Information Processing	Signal transduction	0.01351705
Phospholipase D signaling pathway	ko04072	Environmental Information Processing	Signal transduction	0.02227523
NOD- like receptor signaling pathway	ko04621	Organismal Systems	Immune system	0.02627251
Cholinergic synapse	ko04725	Organismal Systems	Nervous system	0.03768373
Phosphatidylinositol signaling system	ko04070	Environmental Information Processing	Signal transduction	0.03980669
PI3K- Ak t signaling pathway	ko04151	Environmental Information Processing	Signal transduction	0.0727284
ECM- receptor interaction	ko04512	Environmental Information Processing	Signaling molecules and interaction	0.09450136
