# Supplementary material for: Molecular regulatory mechanisms of dietary supplementation with Allium mongolicum Regel powder to improve muscle development and meat quality in Angus calves
Source: Anim Biosci. 2025 Feb 27;38(8):1798–816. doi: 10.5713/ab.24.0809 (PMC12229934; doi:10.5713/ab.24.0809)
Supplement: Supplementary file 5 [file ab-24-0809-Supplementary-5.pdf]

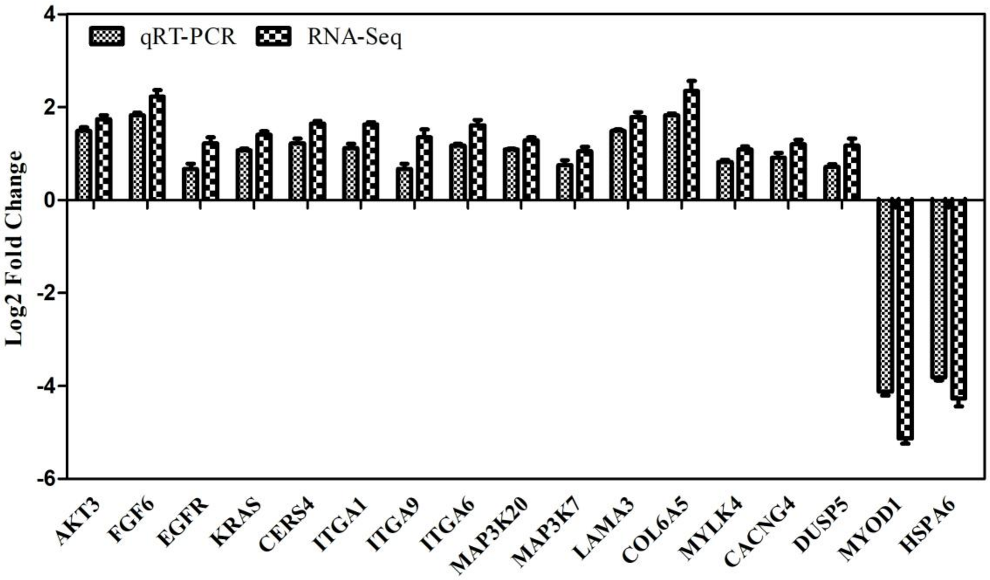

Supplement 5. Expression levels of 17 randomly selected differentially expressed genes by qRT–PCR. The X-axis indicates the seventeen DEGs, and the Y-axis indicates the relative fold change. The values are shown as the mean ± SD (n=5)
